# Synthesis and Strong Solvatochromism of Push-Pull Thienylthiazole Boron Complexes

**DOI:** 10.3390/molecules27175510

**Published:** 2022-08-27

**Authors:** Martijn J. Wildervanck, Reinhard Hecht, Agnieszka Nowak-Król

**Affiliations:** 1 Institut für Anorganische Chemie and Institute for Sustainable Chemistry & Catalysis with Boron, Universität Würzburg, 97074 Würzburg, Germany; 2 Institut für Organische Chemie and Center for Nanosystems Chemistry, Universität Würzburg, 97074 Würzburg, Germany

**Keywords:** solvatochromism, donor–acceptor, fluorescence, hydrazone, Lippert–Mataga plot, push–pull thienylthiazole, tetracoordinated boron

## Abstract

The solvatochromic behavior of two donor-π bridge-acceptor (D-π-A) compounds based on the 2-(3-boryl-2-thienyl)thiazole π-linker and indandione acceptor moiety are investigated. DFT/TD-DFT calculations were performed in combination with steady-state absorption and emission measurements, along with electrochemical studies, to elucidate the effect of two different strongly electron-donating hydrazonyl units on the solvatochromic and fluorescence behavior of these compounds. The Lippert–Mataga equation was used to estimate the change in dipole moments (Δ*µ*) between ground and excited states based on the measured spectroscopic properties in solvents of varying polarity with the data being supported by theoretical studies. The two asymmetrical D-π-A molecules feature strong solvatochromic shifts in fluorescence of up to ~4300 cm^−^^1^ and a concomitant change of the emission color from yellow to red. These changes were accompanied by an increase in Stokes shift to reach values as large as ~5700–5800 cm^−^^1^. Quantum yields of ca. 0.75 could be observed for the *N*,*N*-dimethylhydrazonyl derivative in nonpolar solvents, which gradually decreased along with increasing solvent polarity, as opposed to the consistently reduced values obtained for the *N*,*N*-diphenylhydrazonyl derivative of up to ca. 0.20 in nonpolar solvents. These two push–pull molecules are contrasted with a structurally similar acceptor-π bridge-acceptor (A-π-A) compound.

## 1. Introduction

Accurately describing the solvatochromic and fluorescence behavior of donor–acceptor (D–A) small organic molecules is fundamental to their application in various research fields from bioimaging and biosensors to photovoltaics and solar cells [1,2,3]. Advances in the detection of spectroscopic properties, together with an ever increasing accuracy of quantum chemical calculations, has bolstered our ability to understand their photophysical and electronic properties and given rise to intensive research over the last decade [4,5,6,7,8]. Spatially separated D–A systems are typically appended to a π-conjugated framework that provides an effective pathway for intramolecular charge transfer (ICT) to occur and, simultaneously, creates a significant difference between ground (*µ_g_*) and excited (*µ_e_*) state electric dipole moments [9]. Due to the polarization effect in push–pull molecules, changes in solvent polarity (polarizability and dielectric constant) play an increased role in affecting either the *µ_g_* or *µ_e_* yielding the characteristic solvatochromic behavior observed upon photoexcitation [10]. The extent to which polarity affects the emissive state of push–pull chromophores depends predominantly on the strength of electron-releasing/accepting groups as well as the size and composition (heteroaromatic/aromatic rings) of the π-linking unit [9]. This polarization between ground an excited states affords researchers the ability to individually fine-tune either HOMO or LUMO energies by increasing, or decreasing, the strength of donor or acceptor building blocks. Consequently, increasing the donor strength can efficiently destabilize the HOMO level while leaving the LUMO unperturbed. Similarly, an increase in the electron-withdrawing character of acceptor moieties stabilizes the LUMO leaving the HOMO relatively unchanged [11,12].

Push–pull molecules are of relevance in the field of second-order nonlinear optics [13,14,15]. Moreover, they show promising applications as piezochromic materials [16], sensors [17] and solvatochromic probes [18]. Additional fine-tuning can be achieved via the incorporation of a boron atom into the push–pull arrangement, a well-established strategy for manipulating the molecular electronic behavior. As one could expect, three-coordinate organoboron compounds typically show substantial solvatochromic response [19,20,21,22], while in four-coordinate boron complexes the influence of solvent polarity is, with some exceptions, much weaker [23,24,25,26,27,28].

**Figure 1:**
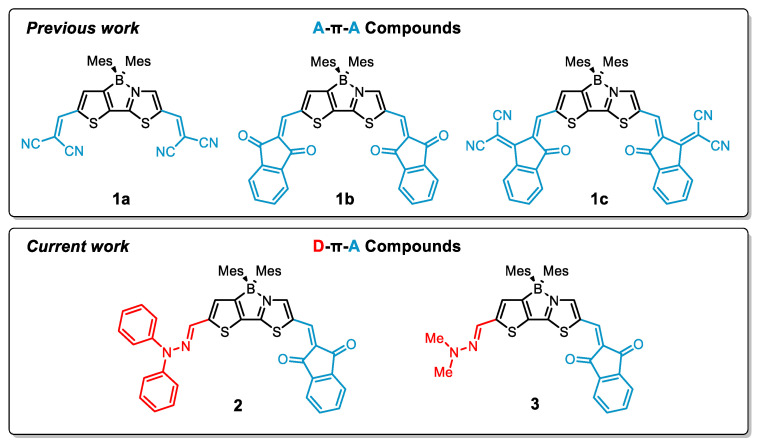
Structural comparison between previous (compounds **1a**–**c**) and current (compounds **2** and **3**) work.

Previously, our group investigated A-π-A systems (Figure 1, top) [29] derived from 2-(3-boryl-2-thienyl)thiazole, in which a four-coordinate boron motif, substituted with two sterically demanding mesityl (Mes) groups, bridges two subunits of a thienylthiazole framework [29]. We converted this scaffold, originally introduced by Yamaguchi and co-workers [22], into a dialdehyde component, which was further functionalized via Knoevenagel condensation with three selected acceptor moieties, namely malononitrile, indandione (IND) and 1,1-dicyanomethylene-3-indanone. The incorporation of a boron atom and additional electron-deficient moieties produced scaffolds with extended π-conjugation and low-lying LUMO levels, which were successfully applied as n-channel semiconductors in organic thin film transistors.

Indandione, as opposed to the two other electron-deficient CH-acids, reacted with the dialdehyde in a sluggish fashion preferentially favoring the thiazole unit of the boryl-thienylthiazole core. This allowed us to isolate a mono-reacted product, in addition to the symmetric A-π-A system, whereby the second formyl group on the thienyl side remained available for further functionalization. The isolation of this product prompted us to design D-π-A systems consisting of hydrazonyl moieties. Owing to their attractive physicochemical properties, hydrazones have been investigated for use as molecular switches [30], ultrafast photonics [31] or probes for sensing [32], to name a few. Additionally, use of a hydrazonyl moiety has a number of attractive features, i.e., it displays a certain degree of rigidity, forms a π-conjugated system and exhibits a strongly electron-donating character. Nonetheless, inclusion of hydrazonyl functionality into π-conjugated scaffolds is much less explored in comparison to the substitution of analogous compounds with amino groups. Since previous observations have indicated that the hydrazone moiety appended to the thienyl ring provides efficient second- or third-order non-linear optical materials [33,34,35], we envisaged that this group will also contribute to a strong charge transfer in our D-π-A molecules. To functionalize our dialdehyde, we selected two hydrazines, i.e., *N*,*N*-diphenylhydrazine and *N*,*N*-dimethylhydrazine as the reactive components generating the D-π-A systems (Figure 1, bottom), whereas the voluminous Mes groups provide satisfactory kinetic stabilization of the boron atom.

In this study, we found that the incorporation of the strongly electron-releasing hydrazonyl functions to the boryl-substituted thienylthiazole core can produce D-π-A systems highly sensitive to environmental polarity with significant solvatochromic shifts in emission covering a broad spectral range. As we demonstrate by combined experimental and theoretical studies, this behavior originates from significant differences between the ground and excited state dipole moments.

## 2. Results and Discussion

### 2.1. Synthesis

**Scheme 1:**
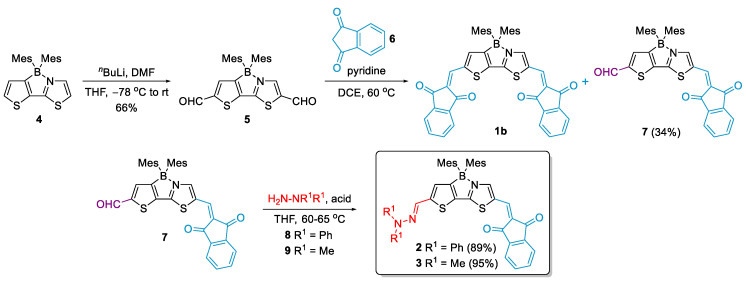
Synthesis of D-π-A compounds **2** and **3**.

The synthesis of the target D-π-A is shown in Scheme 1. Dimesitylboryl complex **4** was prepared according to the modified method published by Yamaguchi and co-workers [29,36]. Formylation thereof with *n*BuLi and DMF afforded dialdehyde **5**. Subsequent Knoevenagel condensation of **5** with indandione (**6**) as an CH-acid produced A-π-A molecule **1b**, which was studied as an electron-transport material in OTFT devices [29], in addition to the singly-condensated product **7** in 34% yield. The structure of the latter compound was assigned based on the 2D NMR studies. Firstly, the NOESY experiment (Figure S4) showed a cross-peak between an aldehyde proton at 9.97 ppm and an aromatic proton, whose signal is overlapping with the signal corresponding to indandione protons to give a multiplet at 7.91–7.85 ppm. Secondly, in the ^1^H,^15^N HMBC spectrum (Figure S5) we found a correlation between the thiazole nitrogen and an aromatic proton signal at 8.39 ppm with ^2^*J*_H-N_ = 5.1 Hz. Thus, the correlations of the aldehyde proton and nitrogen were observed for two different aromatic protons of the 2-(3-boryl-2-thienyl)thiazole. On this basis it may be inferred that, as expected, the reaction of dialdehyde **5** with **6** involves a more reactive thiazole formyl group. Compound **7** offers the possibility to synthesize a large variety of asymmetrically substituted boron complexes of thienylthiazoles. In this study, we used this molecule to construct two D-π-A systems with strongly electron-donating hydrazonyl groups. To this end, **7** was reacted with two hydrazine derivatives, i.e., *N*,*N*-diphenylhydrazine hydrochloride, and *N*,*N*-dimethylhydrazine in the presence of *p*-toluenesulfonic acid. In both cases, the reaction proceeded smoothly affording the desired push–pull molecules in excellent yields (89% and 95%, respectively). NOESY spectra provided invaluable evidence for the structural arrangement of the D-π-A molecules. Here, thiazole and thiophene protons of **3** can be identified by the cross-peaks (Figure S14) between the aromatic (singlets at 8.21 and 7.07 ppm) and CH_3_ mesityl protons (singlet at 2.17 ppm). Moreover, a correlation between the hydrazonyl methyl protons of **3** (singlet at 3.04) and CH=N (singlet at 7.24 ppm) was observed. The latter protons showed coupling with the proton at 7.07 ppm, unambiguously assigned as the thiophene proton. An analogous conclusion could be drawn from the NOESY spectrum of compound **2** (Figure S9).

### 2.2. Photophysical and Solvatochromic Properties

**Figure 2:**
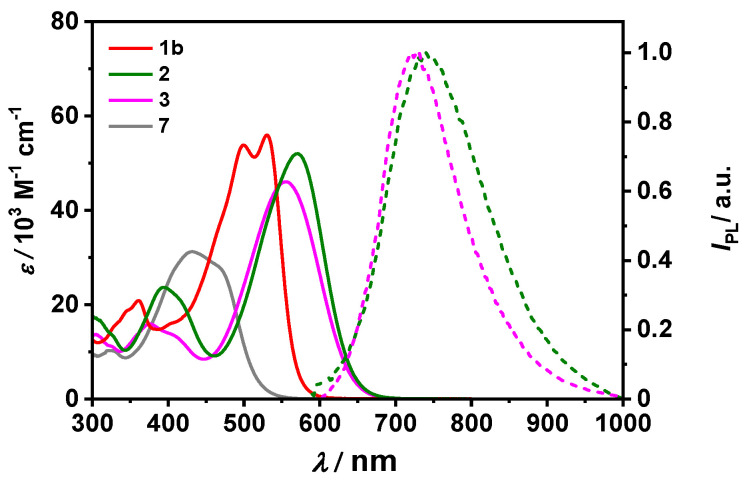
UV/Vis absorption spectra of dyes **2**, **3**, **7**, and **1b** (solid lines) measured at *c ~* 10^−5^ M in CH_2_Cl_2_ and fluorescence spectra of **2** and **3** (dashed lines) determined in CH_2_Cl_2_ by optical dilution method (OD < 0.05).

**Figure 3:**
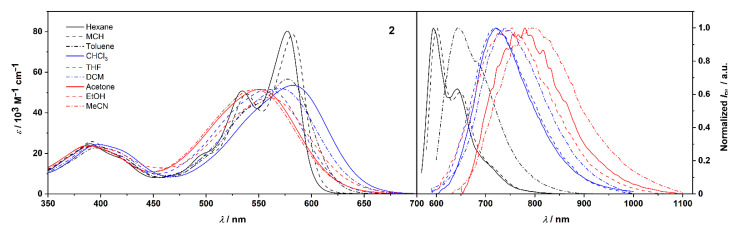
UV/Vis absorption spectra of **2** in solvents of varied polarity measured at *c* = 10^−5^ M–10^−6^ M (**left**). Fluorescence spectra in solvents of varied polarity (colors according to relative permittivity *ε*_r_: black lines for *ε*_r_ < 3; blue lines for 3 < *ε*_r_ < 10; red lines for *ε*_r_ > 10) determined by the optical dilution method (OD < 0.05) (**right**).

**Figure 4:**
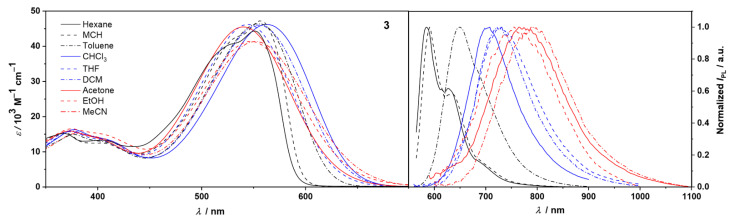
UV/Vis absorption spectra of **3** in solvents of varied polarity measured at *c* = 10^−5^ M–10^−6^ M (**left**). Fluorescence spectra in solvents of varied polarity (colors according to relative permittivity *ε*_r_: black lines for *ε*_r_ < 3; blue lines for 3 < *ε*_r_ < 10; red lines for *ε*_r_ > 10) determined by the optical dilution method (OD < 0.05) (**right**).

Optical properties of D-π-A systems **2** and **3** were investigated by steady-state absorption and emission spectroscopy in methylene chloride. Their absorption profiles were contrasted with those of the singly-condensated derivative **7** and structurally similar A-π-A **1b** (see Figure 2 and Table 1). The lowest-energy absorption band of compound **7** bearing a formyl group on the thiophene ring is positioned at 432 nm. The introduction of the hydrazonyl groups to parent molecule **7** has a tremendous impact on the optical properties. In absorption spectra, a significant red-shift was observed for both compounds, being more pronounced than upon attachment of the second indandione unit in **1b** (*λ*_abs_ = 530 nm) [29]. The lowest-energy absorption bands of **2** and **3** are bathochromically shifted by ca. 1400 and 900 cm^−1^ vs. that of structurally related A-π-A dye **1b**. Square transition dipole moments of 91, and 88 D^2^ are lower than for the latter compound. Furthermore, both D-π-A dyes are weakly emissive in DCM with *Φ*_fl_ of 0.01 and 0.07, respectively. The corresponding emission spectra are presented in Figure 2, Figure 3 and Figure 4. The fluorescence peak maxima are observed at 740 nm and 726 nm, corresponding to the large Stokes shifts of ca. 4000 cm^−1^ and 4200 cm^−1^, respectively (Table 1, Figure 2). On the other hand, the emission of **1b** was effectively quenched. These results may suggest charge transfer interactions as one of the possible deactivation pathways for all three A-π-A systems, as well as D-π-A molecules.

We investigated the solvent effect on the absorption and emission properties of compounds **2** and **3**, for a set of nine solvents of varied polarity, both protic and aprotic (Table 2). These studies were made feasible due to a satisfactory solubility of these D-π-A systems in a wide range of solvents. The corresponding spectra for both compounds are depicted in Figure 3 and Figure 4. The colors of **2** and **3** in solutions of the studied solvents are shown in Figures S16 and S17.

Absorption spectra of D-π-A molecules in nonpolar solvents (Figure 3 and Figure 4 left) show vibronic fine structure. The maxima of the lowest-energy bands of molecule **2** are centered at 577 and 582 in *n*-hexane and MCH, respectively. These correspond to S_0,0_-S_1,0_ transitions and are followed by distinct S_0,0_-S_1,1_ vibronic progressions at 535 and 539.

Additionally, much weaker transitions are observed at around 500 nm in these solvents. In toluene, the vibronic manifold is strongly affected, although still visible, while in more polar solvents absorption bands are structureless. As suggested by DFT calculations (below), the loss of vibronic fine structure can be attributed to the intramolecular charge transfer from donor to acceptor moieties. An increase in the solvent polarity entails a significant broadening and a concomitant drop of molar absorption coefficients *ε*_max_ of S_0_ → S_1_ transitions as a consequence of increased solute–solvent interactions. Nevertheless, the square transition dipole moments remain roughly constant (90 ± 3 D^2^) independent of the solvent polarity (Table 2). The absorption profiles of **3** in *n*-hexane and MCH are broader than they are for **2** with the vibronic structure being preserved in both solvents (Figure 4). Note that when compared to the corresponding spectra of **2**, the ratio of 0–1 to 0–0 transitions is significantly increased in favor of the 0–1 transition. Moreover, lowest-energy transition bands in the spectra of **3** are described by the slightly lower square transition dipole moments than the corresponding transitions in spectra of **2**, as well as a hypsochromic shift due to a smaller π-conjugated system.

A more profound solvent effect was observed in fluorescence spectra (Figure 3 and Figure 4 right and Figures S16b and S17b). Similarly to the absorption spectra, the fluorescence spectra of **2** and **3** in *n*-hexane and MCH display vibronic fine structures, although with a distorted image relationship between absorption and emission. This loss of mirror symmetry may already suggest a modification of the geometry in the excited state.

The sharp emission bands of **2** are positioned at 595 and 601 nm with distinct vibronic progressions at 642 and 648 nm in *n*-hexane and MCH, respectively, in addition to, weak vibronic bands beyond 700 nm in both solvents. These correspond to S_1,0_-S_0,0_, S_1,0_-S_0,1_, and S_1,0_-S_0,2_ transitions from the equilibrated excited state to the Franck–Condon ground state. An exchange of phenyl for methyl groups results in a slight hypsochromic shift of the corresponding emission peak maxima to 585 and 591 nm in *n*-hexane and MCH, respectively. As the solvent polarity increases, considerable broadening of the line shape and the loss of the vibronic fine structure are observed. Noteworthy, these changes are accompanied by a dramatic displacement of fluorescence maxima. The emission peaks in MeCN are centered at 800 and 774 nm for **2** and **3**, respectively. Accordingly, overall red-shifts of 4310 cm^−^^1^ (**2**) and 4170 cm^−^^1^ (**3**) can be observed on going from *n*-hexane to MeCN, which for **3** is comparable to environmentally sensitive pyrimidine-bisboron complexes reported by Kubota and Matsui (up to 4230 cm^−^^1^) [25,37] and a keto-isoindolinyl pyridyl-containing boron-complex published by Lu and Shen (4410 cm^−^^1^) [38]. Thus, both chromophores show strong positive solvatochromism in emission, which implies that their dipole moments in the excited state are much larger than in the ground state [39]. In addition, a more effective stabilization of the excited state accounts for the successive increase in Stokes shifts ($\Delta \tilde{\nu }$) with increasing solvent polarity. The large $\Delta \tilde{\nu }$ values in polar solvents, i.e., 5750 and 5700 cm^−1^ in MeCN for **2** and **3**, respectively, vs. their respective values in *n*-hexane of 520 and 1060 cm^−1^ indicate a strong CT character of the emissive state. Furthermore, the fluorescence quantum yields gradually decreased with increasing relative permittivity of the solvent. This trend correlates well with the gradual increase in Stokes shifts and can be attributed to the accelerated internal conversion from the excited state due to a reduced energy gap between excited and ground states. Accordingly, compound **3** showed remarkably high *Φ*_fl_ of 0.75 and 0.73 in nonpolar solvents, such as *n*-hexane and MCH, respectively, while *Φ*_fl_ of **2** was significantly reduced to 0.11 and 0.19 in these solvents. This most likely results from conformational relaxation associated with the phenyl groups of the hydrazonyl moiety. As the solvent polarity increased, a drop in the *Φ*_fl_ values for **2** and **3** was observed. For instance, *Φ*_fl_ of **3** in toluene was ca. 1.8-fold lower than in both *n*-hexane and MCH. In the most polar solvents, fluorescence was almost entirely diminished (*Φ*_fl_ = ~0.01).

Large solvatochromic shifts accompanied by a significant quenching of fluorescence might infer formation of a twisted intramolecular charge transfer (TICT) state [40], a phenomenon observed in push–pull systems and molecular rotors [41]. This type of behavior has been observed for donor–acceptor systems resulting from the rotational motion of amino donor groups appended to the π-bridge through a C-C single bond [42,43]. To verify the possibility of TICT formation for D-π-A systems **2** and **3**, we performed fluorescence measurements in solvents of high viscosity, i.e., ethylene glycol (EG) (*η* = 19.8 cP at 20 °C) and glycerol (*η* = 1410 cP at 20 °C). The intramolecular motions in these media are slower which, in the case of TICT, is accompanied by a decrease in the contribution from internal conversion resulting in a more intense emission from a conformationally non-equilibrated yet excited state. Both experiments indicated the absence of a TICT state as the fluorescence intensity in these highly viscous solvents showed no observable enhancement. The *Φ*_fl_ of **2** measured in EG decreased to 1.7%, whereas it was completely quenched for **3** in accordance with increasing solvent permittivity. The latter compound proved insoluble in glycerol. Therefore, the effect of this medium could only be verified for phenyl derivative **2**. Interestingly, the fluorescence quantum yield measured in glycerol was 1.9%, which is comparable to the value obtained in EG even though the solvent permittivity of glycerol (*ε*_r_ = 47) is higher than of EG (*ε*_r_ = 37). We believe that a partial suppression of the nonradiative process can be assigned to a somewhat inhibited rotation of the hydrazonyl phenyl rings around a single C-C bond and the NPh_2_ unit around an N-N bond in this highly viscous medium. Nevertheless, the evidence clearly indicates that a decrease in the radiative deactivation in D-π-A systems is not due to formation of a TICT state.

The excited state dynamics were further studied with time-resolved fluorescent measurements and revealed short lifetimes for both compounds (residual plots are provided in the Supplementary Materials). D-π-A **2** displayed biexponential decay profiles in different solvents, apart from MCH, with *τ*_1_ values < 1.0 ns and *τ*_2_ in the 1.0–2.3 ns range, whereas the fluorescence decay lifetimes for MeCN and highly polar EtOH could not be determined. These *τ* values do not show any considerable solvent polarity-dependent changes. In each case, the shorter *τ*_1_ component showed significantly larger contribution (relative amplitudes of 78.1–96.4%), while the contribution of the longer lifetime component *τ*_2_ was much smaller (3.6–21.9%). In addition, the relative amplitudes reveal a trend where the contribution of *τ*_1_ decrease while, conversely, those of *τ*_2_ increase concomitantly with solvent polarity. The *τ*_1_ component corresponds, therefore, to the emission from the locally excited state, while *τ*_2_ can be assigned to the solvent–solute interactions, which increase with solvent polarity, and the ICT state. These results mirror the trend observed in the fluorescence quantum yields with decreasing values and are similarly assigned to the geometry relaxation associated with the hydrozonyl phenyl rings. In contrast, monoexponential decays were observed for **3**, except in THF and acetone, with the comparably higher *τ*_1_ values of 2.7, 2.8 and 2.9 ns for non-polar solvents MCH, *n*-hexane and toluene, respectively. A general lowering of the *τ*_1_ values is observed for the solvents of increasing polarity, with the exception of MeCN, similarly to compound **2**.

Solvent-dependence was also investigated for A-π-A molecule **1b**, although the studies were limited to the narrower range of solvents due to lower solubility of **1b** when compared to the push–pull compounds. The data collected for **1b** are listed in Table S1 and the corresponding absorption and emission spectra are depicted in Figures S33 and S34. The change of the solvent had a small effect on the positions and the line shapes of the absorption bands of indandione derivative (Figure S33). Conversely, the increase in solvent permittivity led to a substantial shift of its emission maxima from 612 nm in MCH to 688 nm in CHCl_3_ (Figure S34). Moreover, the fluorescence quantum yields were largely reduced in this set of solvents, or entirely quenched in media of higher polarity. Unlike absorption bands, emission bands of **1b** were devoid of vibronic fine structure. These features along with the increasing values of Stokes shifts (up to ~4500 cm^−1^ in CHCl_3_) can be attributed to the efficient intramolecular charge transfer and a dissipation of energy due to the excited state geometry alteration.

### 2.3. Calculation of Ground and Excited State Dipole Moments

To estimate the change in dipole moment (Δ*μ*) between excited (*μ*_e_) and ground (*μ*_g_) states of compounds **2** and **3**, the Lippert–Mataga [44,45] equation (Equation (1)) was used:

(1) $$\Delta \tilde{\nu }={\tilde{\nu }}_{a}-{\tilde{\nu }}_{\mathrm{em}}=\frac{1}{4\pi \varepsilon_{0}}\cdot \frac{2\Delta \mu^{2}}{hca^{3}}\;\Delta f+\mathrm{const}$$

where $\Delta \tilde{\nu }$ denotes the Stokes shift in wavenumber; ${\tilde{\nu }}_{a}$ *and* ${\tilde{\nu }}_{\mathrm{em}}$ are the absorption and emission maxima in wavenumbers, *ε*_0_ is the vacuum permittivity; *h* is the Plank constant, *c* is the light velocity, *a* is the radius of the Onsager cavity, and Δ*f* is the orientational polarizability of the solvent, which is the function of the relative permittivity *ε* and refractive index *n* of the solvent and can be calculated as follows (Equation (2)):

(2) $$\Delta f=\frac{\varepsilon_{r}-1}{2\varepsilon_{r}+1}-\frac{n^{2}-1}{2n^{2}+1}$$

**Figure 5:**
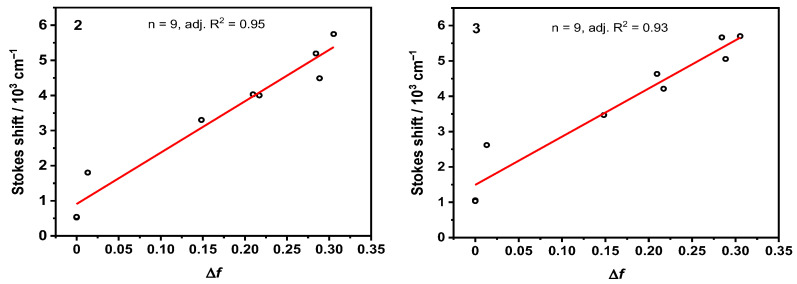
Lippert–Mataga plots of compounds **2** (**left**) and **3** (**right**).

The Onsager cavity radii [46] and the ground state dipole moments were calculated using density functional theory (DFT) at the B3LYP-D3(BJ)/def2-SVP level of theory in a vacuum. Here, the corresponding values of the Onsager cavity radii are 6.23 and 5.85 Å, while ground state dipole moments are 4.6 and 6.8 D for **2**, and **3**, respectively. The corresponding Lippert–Mataga plots for these compounds are presented in Figure 5.

In both cases, the regression analysis yielded only minor variance with an adj. *R*^2^ of 0.95 (**2**) and 0.93 (**3**). The Δ*μ* values were obtained from the slope *m* of the plots calculated at 18.5 and 16.6 D for compounds **2**, and **3**, respectively, allowing their respective *μ*_e_ values of 23.3 and 23.3 D to be determined. The large difference in permanent dipole moments between the *μ*_e_ and *μ*_g_ values accounts for the strong solvatochromism observed.

### 2.4. Quantum Chemical Calculations

**Figure 6:**
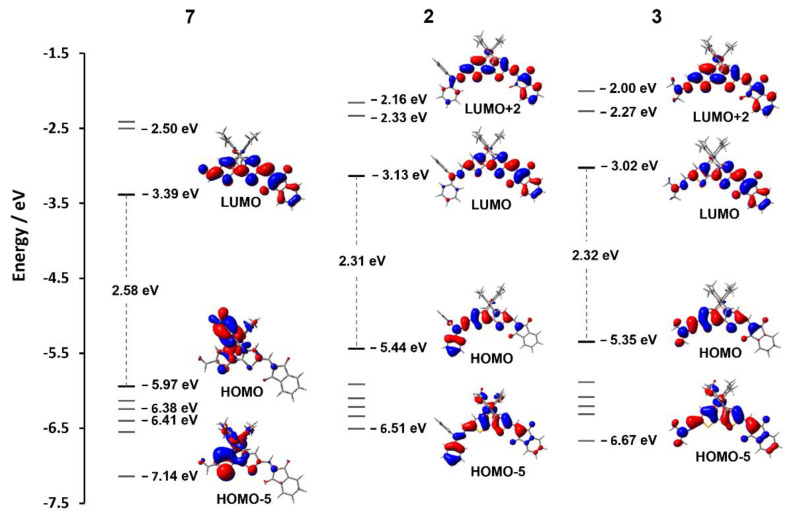
Kohn–Sham orbital energy level diagram (B3LYP-D3(BJ)/def2-SVP, CH_2_Cl_2_, PCM) for compounds **7**, **2** and **3**.

The effect of incorporating hydrazonyl functionality into the BMes_2_-thienylthiazole scaffold was elucidated using DFT calculations at the B3LYP [47,48,49] -D3(BJ) [50]/def2-SVP [51] level of theory with the inclusion of solvent (CH_2_Cl_2_, PCM model). The calculated geometries of **2** and **3** revealed an essentially planar structure when comparing the ground and excited states. Kohn–Sham orbitals of the two D-π-A systems displayed HOMO levels predominantly spread across the hydrazonyl-boryl-thienylthiazole core (Figure 6), while the LUMO levels are distributed more towards the indandione moiety. These are strikingly different to the distributions of **1a–c [29],** having a symmetric A-π-A configuration, and monoaldehyde precursor **7** where the HOMOs are primarily localized on one mesityl ring of the BMes_2_ group and the LUMOs are distributed across the acceptor-thienylthiazole-acceptor framework. Attachment of the hydrazonyl moieties to **7** causes visible destabilization of the HOMO level increasing from −5.97 eV for **7** to −5.44 and −5.35 eV for **2** and **3**, respectively.

According to TD-DFT calculations (CAM-B3LYP [52] -D3(BJ)/def2-SVP, CH_2_Cl_2_, PCM model) the lowest-energy absorption bands of **2** and **3** are predominantly attributed to HOMO → LUMO transitions (80–87%) with minor contributions from HOMO-5 → LUMO (4–5%) and HOMO → LUMO+2 (5%), (Figure 6). The spatial separation between HOMO and LUMO density while simultaneously having the highest excitation probability, reinforces the charge transfer nature from donor π-bridge to acceptor. Indeed, TD-DFT analysis of the emission energies calculated in solvents with increasing polarity show a progressive stabilization of the LUMO levels qualitatively reflecting the experimental fluorescence spectra.

The efficiency of the energy transfer depends on the conformational flexibility within each molecule. Our studies revealed that optimized geometries of both **2** and **3** display an essentially planar arrangement of the π-bridge indicating an efficient electron transport between D and A units. Energetically preferable conformations of **2** are achieved when one phenyl ring lies in plane with the boryl-thienylthiazole backbone, hence extending the conjugated system, while **3**, bearing methyl groups at the hydrazonyl moiety, has an essentially planar structure overall.

A change in dipole moment and polarizability between ground and excited state is another clear and well understood indication for the occurrence of intramolecular charge transfer. An accurate description of both ground and excited state dipole moments is therefore necessary to understand the transfer process. Calculated *μ*_g_ are centered around ~5 D in the case of **2** and ~8–10 D for **3**, remaining relatively unperturbed with solvent variation (Table 3). As expected, optimization of the S_1_ geometry shows a dramatic shift in the *μ*_e_ typical of push–pull chromophores indicating the molecules are much more polar in their respective S_1_ states. Values of *μ*_e_ ranging between ~12.5–15 D and ~13.5–18 D were calculated for the dominant transitions of **2** and **3**, respectively, confirming the ICT character of the structures. This suggests the observed increase in Stokes shifts results from a stabilization of the LUMO rather than a destabilization of the HOMO. This is also intuitive as the absorption *λ*_abs_ (S_0_ → S_1_) for each compound remains relatively unshifted. Although these values differ somewhat from the experimental, readily accurate dipole moments are often difficult to obtain due to the compounded errors associated with both methods for obtaining the respective theoretical and experimental data [53,54]. A comparison between these dipole moments is an ongoing area of investigation [55,56,57,58].

### 2.5. Electrochemistry

**Figure 7:**
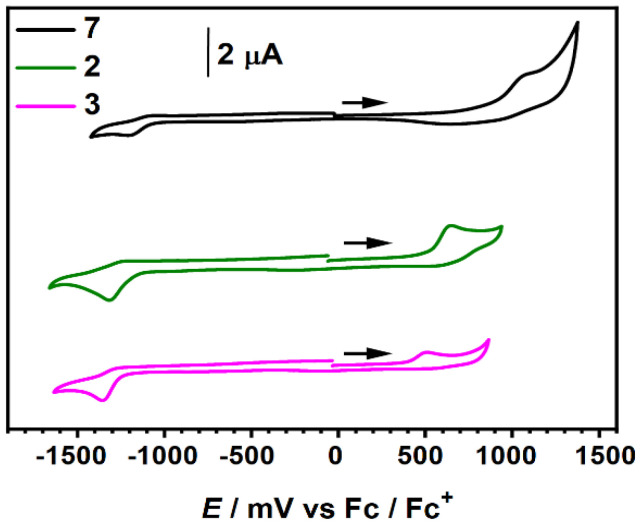
Cyclic voltammogram of **2**, **3** and **7** calibrated vs. ferrocene/ferrocenium (Fc/Fc^+^) (*c* ~ 10^−4^–10^−5^ M in dry CH_2_Cl_2_; scan rate = 100 mV s^−1^, 0.1 M Bu_4_NPF_6_ as a supporting electrolyte, 50% iR compensated).

The electrochemical behavior of the target push–pull molecules and their precursor was studied by cyclic voltammetry (CV) in CH_2_Cl_2_ (Table 1 and Figure 7) in the presence of Bu_4_NPF_6_ as a supporting electrolyte and calibrated versus ferrocenium/ferrocene (Fc^+^/Fc). A voltammogram of molecule **7** bearing two different electron-withdrawing groups reveals single reduction and oxidation events at −1.21 and +1.08 V, respectively. Inclusion of the NNPh_2_ and NNMe_2_ subunits to the 2-(3-boryl-2-thienyl)thiazole π-system resulted in distinct alterations of the redox properties. Both D-π-A molecules **2** and **3** are readily oxidized at such low potentials as +0.65 and +0.51 V, respectively, due to the presence of strongly electron-donating hydrazonyl substituents. Thus, the oxidation potentials were shifted by over 0.4 V, whereas reduction potentials by only 0.11–0.14 V compared to parent compound **7**. Redox potentials of **3** displayed even lower values when compared with **2** reflecting a stronger electron-releasing character of the dimethyl- compared to the diphenyl-hydrazonyl moiety. The energy levels of FMOs were determined from the CV measurements assuming the energy level of Fc/Fc^+^ to be at −5.15 vs. vacuum for comparison with the structurally similar A-π-A molecule **1b** [29]. Attachment of hydrazonyl substituents substantially increased the FMO energies, while maintaining band gaps below 2.0 eV. Accordingly, HOMO levels of **2** and **3** were lifted to −5.80, and −5.66 eV, respectively while energy levels associated with the LUMO were less affected. As demonstrated, we were able to significantly tune the electrochemical behavior of the parent boryl-substituted thienylthiazole by either substitution of the core by electron-withdrawing groups, such as formyl, or formation of D-π-A systems where the core serves as a π-bridge between the indandione attached via an ethylene linker and strongly electron-releasing hydrazonyl groups.

## 3. Materials and Methods

### 3.1. General Information

All reagents were purchased from commercial sources and used without further purification, unless otherwise stated. Reagent grade solvents were distilled prior to use. Column chromatography was performed on silica (silica gel, 230–400 mesh). ^1^H, ^13^C and ^11^B NMR, and 2D NMR spectra were recorded on a Bruker Avance 400 spectrometer. ^1^H and ^13^C NMR spectra were calibrated to the residual solvent signals. ^11^B NMR spectra were calibrated to the signal of boron trifluoride diethyl etherate (BF_3_·Et_2_O) as external standard. *J* values are given in Hz. The following abbreviations were used to designate multiplicities: s = singlet, d = doublet, t = triplet, m = multiplet. High resolution mass spectra were obtained by electrospray ionization (ESI) and were recorded on an ESI microOTOF Focus spectrometer from Bruker Daltonics. Low resolution mass spectra were obtained by matrix-assisted laser desorption/ionization (MALDI) and were recorded on an Autoflex II MALDI-TOF mass spectrometer (Bruker Daltonics GmbH). 1,3-indandione was commercially available. Compounds **4** [29,36] and **5** [29] were prepared according to reported procedures.

### 3.2. Synthetic Procedures

**Synthesis of 7.** To dialdehyde **5** (100 mg, 0.21 mmol) and compound **6** (124 mg, 0.85 mmol) in dichloroethane (8 mL) dry pyridine (0.17 mL, 2.11 mmol) was added and the reaction mixture was stirred at 60 °C for 2.5 h. Then the solution was loaded on a column and purified (silica, CH_2_Cl_2_) to give products **7** (43 mg, 34%) and **1b**. The latter was crystallized (CH_2_Cl_2_/pentane) to give pure **1b** (81 mg, 53%) as a dark red solid. Compound **1b**: ^1^H NMR (400 MHz, CD_2_Cl_2_, 25 °C) δ 8.41 (s, 1H), 8.07–7.95 (m, 6H), 7.89–7.81 (m, 5H), 6.68 (s, 4H), 2.17 (s, 6H), 1.90 (s, 12H). The analytical data are in accordance with the literature [29]. Compound **7**: UV/vis (CH_2_Cl_2_, *c* = 9.63 × 10^−6^ M): *λ*_max_/nm 432 (ε/M^−1^ cm^−1^ 31 200) with a shoulder at ~466 nm, 323 (10 300), 271 (26 000). ^1^H NMR (400 MHz, CD_2_Cl_2_, 25 °C) δ 9.97 (s, 1H), 8.39 (s, 1H), 8.06–7.96 (m, 2H), 7.91–7.85 (m, 3H), 7.83 (s, 1H), 6.67 (s, 4H), 2.17 (s, 6H), 1.87 (s, 12H). ^13^C NMR (101 MHz, CD_2_Cl_2_, 25 °C) δ 190.3, 188.7, 184.9, 171.4, 153.1, 148.8, 142.4, 141.4, 140.4, 138.7, 136.6, 136.5, 136.1, 135.3, 132.8, 131.4, 130.4, 128.4, 124.0, 124.0, 24.9, 20.9 (two carbon signals corresponding to the C atoms bound to the B atom are not visible due to the quadrupolar relaxation). MS HR (ESI) *m*/*z* calcd for C_36_H_31_BNO_3_S_2_ [M+H]^+^ 600.1833, found 600.1821.

**Synthesis of 2.** A flask was charged with compound **7** (15 mg, 0.025 mmol), and *N*,*N*-diphenylhydrazine hydrochloride (**8**; 5.5 mg, 0.25 mmol). Then THF (0.6 mL) was added, and the reaction mixture was stirred at 65 °C for 50 min. Afterwards, the solvent was evaporated and the crude product was purified by column chromatography (silica, CH_2_Cl_2_/pentane 7:3). Crystallization (CH_2_Cl_2_/pentane) afforded pure **2** (17 mg, 89%) as a black solid. UV/vis (CH_2_Cl_2_, *c* = 1.18 × 10^−5^ M): *λ*_max_/nm 571 (ε/M^−1^ cm^−1^ 52 000), 394 (23 700), 267 (32 200) with a shoulder at ~305. ^1^H NMR (400 MHz, CD_2_Cl_2_) δ 8.24 (s, 1H), 8.04–7.92 (m, 2H), 7.88–7.81 (m, 2H), 7.79 (m, 1H), 7.49–7.38 (m, 4H), 7.30–7.21 (m, 3H), 7.21–7.14 (m, 4H), 7.01 (s, 1H), 6.63 (s, 4H), 2.15 (s, 6H), 1.86 (s, 12H). ^13^C NMR (101 MHz, CD_2_Cl_2_) δ 190.4, 189.2, 171.9, 155.5, 149.3, 143.3, 142.3, 141.3, 140.4, 136.2, 136.1, 134.8, 132.4, 130.5, 130.4, 130.3, 130.2, 130.0, 129.8, 126.3, 125.9, 123.7, 123.7, 122.9, 24.7, 20.9 (two carbon signals corresponding to the C atoms bound to the B atom are not visible due to the quadrupolar relaxation). ^11^B NMR (128 MHz, CD_2_Cl_2_, 25 °C) δ 3.5 ppm; MS HR (ESI) *m*/*z* calcd for C_48_H_41_BN_3_O_2_S_2_ [M+H]^+^ 766.2728, found 766.2717.

**Synthesis of 3.** A Schlenk tube was charged with compound **7** (17.5 mg, 0.029 mmol), and *p*-toluenesulfonic acid monohydrate (6.5 mg, 0.034 mmol). Then THF (0.6 mL) was added, followed by *N*,*N*-dimethylhydrazine (**9**; 2.3 μL, 0.30 mmol) and the reaction mixture was stirred at 60 °C for 2 h. Afterwards, the solvent was evaporated and the crude product was purified by column chromatography (silica, CH_2_Cl_2_/pentane 4:1). The pure compound was recrystallized (CH_2_Cl_2_/pentane) to give **3** (18 mg, 95%) as a black solid. UV/vis (CH_2_Cl_2_, *c* = 1.66 × 10^−5^ M): *λ*_max_/nm 556 (ε/M^−1^ cm^−1^ 46 000), 376 (15 900) with a shoulder at ~405 nm, 265 (27 200) with shoulders. ^1^H NMR (400 MHz, CD_2_Cl_2_) δ 8.21 (s, 1H), 8.01–7.91 (m, 2H), 7.87–7.79 (m, 2H), 7.77 (m, 1H), 7.24 (s, 1H), 7.07 (s, 1H), 6.65 (s, 4H), 3.04 (s, 6H), 2.17 (s, 6H), 1.89 (s, 12H). ^13^C NMR (101 MHz, CD_2_Cl_2_) δ 190.5, 189.3, 172.0, 157.7, 149.5, 142.3, 141.3, 140.4, 136.0, 135.9, 134.8, 132.7, 130.2, 129.6, 128.7, 127.3, 125.8, 124.9, 123.6, 123.6, 43.0, 24.7, 21.0 (two carbon signals corresponding to the C atoms bound to the B atom are not visible due to the quadrupolar relaxation). ^11^B NMR (128 MHz, CD_2_Cl_2_, 25 °C) δ 4.7 ppm; MS HR (ESI) *m*/*z* calcd for C_38_H_37_BN_3_O_2_S_2_ [M+H]^+^ 642.2415, found 642.2417.

### 3.3. Computational Details

DFT calculations were performed using the Gaussian 16 [59] program package at the B3LYP [47,48,49]-D3(BJ) [50]/def2-SVP [51] level of theory for ground state geometry optimizations of compounds **2**, **3** and **7**, while those of the electronic S_1_ state employed the CAM-B3LYP [52]-D3(BJ) functional with the same basis set. In the latter case, the Coulomb attenuated parameter case provides a long-range correction for studying compounds where there is probability for charge transfer effects in the electronic excited state, as in the case for D-π-A compounds. The optimizations were followed by frequency calculations which confirmed the presence of minima, the absence of imaginary frequencies. The various solvents investigated were included in all calculations using the PCM solvent model.

Time dependent (TD)-DFT calculations were carried out on the optimized ground state geometries using the CAM-B3LYP functional and def2-SVP basis set with the same solvent model (PCM).

The details regarding the calculations of Onsager cavity are given in the Supplementary Materials.

### 3.4. Absorption Spectroscopy

UV/vis absorption measurements were recorded using either a Lambda 950 (PerkinElmer) or a Jasco V-670. The spectra were measured in spectroscopic grade solvents from ACROS.

### 3.5. Fluorescence Spectroscopy

Steady state-fluorescent measurements for **2** and **3** were performed with an Edinburgh FLS980 spectrophotometer equipped with an NIR PMT detector for recording emission beyond 850 nm. Fluorescence lifetimes were measured by time correlated single-photon counting (TCSPC) using an EPL picosecond pulsed laser diode (506 nm). The instrument response function was collected using a scatterer (Ludox AS40 colloidal silica). Good fit data were obtained when a χ^2^ value was obtained in the 1.0–1.3 range.

Quantum yields were recorded on a QM-4/2003 (PTI) by optical dilution method (OD < 0.05) [60] and were determined as the average for four or five different excitation wavelengths using Oxazine 1 (*Φ*_fl_ (EtOH) = 0.11) [61], *N*,*N*-bis-(2,6-diisopropylphenyl)perylene 3,4:9,10-tetracarboxylic acid bisimide (*Φ*_fl_ (CHCl_3_) = 1.00) [62] or *N*,*N*-bis-(2,6-di-isopropylphenyl)-1,6,7,12-tetraphenoxy-perylene-3,4:9,10-tetracarboxylic acid bisimide (*Φ*_fl_ (CHCl_3_) = 0.96) [62].

### 3.6. Electrochemical Analysis

The CV measurements were performed on a standard, commercial electrochemical analyzer (EC Epsilon; BAS Instruments, UK) in a three-electrode single-compartment cell under an argon atmosphere. Dichloromethane (HPLC grade) was dried over calcium hydride under an argon atmosphere, distilled, and degassed prior to use. The supporting electrolyte NBu_4_PF_6_ was synthesized according to the literature [62], recrystallized from ethanol/water, and dried in a high vacuum. The measurements were carried out under the exclusion of air and moisture at a concentration of ca. 2.5 × 10^−4^ M with ferrocene as an internal standard for the calibration of the potential. Working electrode: Pt disc; reference electrode: Ag/AgCl; auxiliary electrode: Pt wire.

## 4. Conclusions

Introduction of diphenyl and dimethyl hydrazonyl moieties to the boryl-substituted thienylthiazole architecture afforded D-π-A molecules **2** and **3** with exceptional stability under ambient conditions and destabilized LUMO levels when compared to their formyl-substituted precursor **7**. These push–pull molecules display strong positive solvatochromism in emission, with large Stokes shifts up to ca. 5800 cm^−^^1^. The values of solvatochromic shifts as high as ~4200–4300 cm^−^^1^ along with solvent-dependent emission intensity situate these push–pull molecules among four-coordinate boron complexes of remarkably high environmental sensitivity. For instance, **3** showed a dramatic red-shift of its fluorescence maxima values from 585 nm in *n*-hexane (yellow emitter) to 774 and 776 nm in acetonitrile and acetone, respectively (red emitter). In addition, this compound displays high fluorescence quantum yields (up to 0.75) in nonpolar solvents, which gradually decrease on going to more polar solvents. A similar trend in fluorescence and a bathochromic shift is observed for **2**. However, the values for fluorescence quantum yields were dramatically decreased owing to the presence of diphenyl rings on the hydrazone moiety and associated geometry relaxation in the excited state. The difference between these substituents is also evident in the analysis of their fluorescence lifetimes. Accordingly, bi- and mono-exponential decay curves were observed for **2** and **3**, respectively, revealing an additional deactivation pathway for the *N*,*N*-diphenylhydrazonyl derivative. Both compounds displayed short lifetimes with the *τ*_1_ values below 1 ns for compound **2**, while those obtained for **3** ranged between 1 and 2.9 ns, with the exception of more polar solvents. Theoretical studies evaluating the distribution of FMOs and dipole moments in both ground and excited states confirmed the behavior is attributed to intramolecular charge transfer. We envisage that our studies on boron-substituted thienylthiazolyl push–pull molecules could facilitate the future design of nonlinear optics molecules or polarity probes.

## Figures and Tables

**Table 1:** Optical and electrochemical properties of dyes **2**, **3**, **7** and reference compound **1b**.

Dye	*λ*_abs_*^a^* [nm]	*ε*_max_*^b^* [10^3^ M^−1^ cm^−1^]	*μ*^2^_eg_*^c^* [D^2^]	$\mu_{ag}^{2}{M^{-1}\;}^{d}$ [D^2^ mol g^−^^1^]	${E_{1/2}^{red}\;}^{e}$ [V]	${E_{1/2}^{ox}}^{e}$ [V]
**2**	571	52.0	91	0.12	−1.32 *^f^*	+0.65*^f^*
**3**	556	46.0	88	0.14	−1.36 *^f^*	+0.51 *^f^*
**7**	432	31.2	79	0.13	−1.21 *^f^*	+1.08 *^f^*
**1b** [29]	530	55.9	118	0.16	−1.12, −1.49 *^f^*	+0.89 *^f^*, +1.01 *^f^*, +1.17 *^f^*
	499	53.8				

*^a^* Absorption maximum and vibronic progression in CH_2_Cl_2_. *^b^* Molar absorption coefficient in CH_2_Cl_2_. *^c^* Square transition dipole moment calculated for the lowest-energy transition of the UV/Vis absorption spectrum in CH_2_Cl_2_. *^d^* Tinctorial strength. *^e^* Redox potentials were measured in dry CH_2_Cl_2_ (*c* ~ 10^−4^–10^−5^ M) at a scan rate of 100 mV s^−1^ and with 50% of iR compensation; supporting electrolyte Bu_4_NPF_6_ (*c* = 0.1 M). Measurements were calibrated vs. the ferrocenium/ferrocene (Fc^+^/Fc) redox couple as an internal standard. *^f^* Peak potential.

**Table 2:** Optical properties of D-π-A compounds **2** and **3** in solvents of varied polarity.

Cpd	Solvent	*ε*_r_ *^a^*	*λ*_abs_*^b^* [nm]	*ε*_max_*^c^* [10^3^ M^−1^cm^−1^]	*µ*^2^_eg_*^d^* [D^2^]	*λ*_em_*^e^* [nm]	${\Delta \tilde{\nu \;}}^{f}$ [cm^−1^]	*τ*_1_*^g^* [ns] (% amp)	*τ*_2_*^h^* [ns] (% amp)	*Φ*_fl_*^i^* [%]
**2**	*n*-hexane	1.9	577	80.3	89	595	520	0.5 (96.4)	2.12 (3.6)	11 ± 1
	MCH	2.02	582	78.6	89	601	540	0.91	-	19 ± 2
	toluene	2.38	577	56.7	86	644	1800	0.52 (96.3)	2.31 (3.7)	8 ± 1
	CHCl_3_	4.8	583	53.6	91	722	3300	0.44 (93.8)	1.61 (6.2)	3 ± 1
	THF	7.6	558	51.6	90	720	4030	0.3 (90.5)	1.38 (9.5)	2 ± 1
	CH_2_Cl_2_	9.1	571	52.0	91	740	4000	0.19 (81.6)	1.32 (18.4)	1 ± 1
	acetone	20.6	551	51.6	93	772	5200	0.23 (78.1)	1.02 (21.9)	<1
	EtOH	22.4	560	47.4	89	748	4490	-	-	5 ± 1
	MeCN	37.5	548	49.8	93	800	5750	-	-	1 ± 1
**3**	*n*-hexane	1.9	551	44.4	78	585	1060	2.73	-	75 ± 1
	MCH	2.02	557	47.2	78	591	1030	2.77	-	73 ± 1
	toluene	2.38	554	46.4	82	648	2620	2.88	-	41 ± 1
	CHCl_3_	4.8	562	46.2	87	698	3470	1.57	-	15 ± 1
	THF	7.6	545	46.2	88	729	4630	0.69 (93.3)	2.21 (6.7)	5 ± 1
	CH_2_Cl_2_	9.1	556	46.0	88	726	4210	1.02	-	7 ± 1
	acetone	20.6	539	45.5	90	776	5670	0.31 (75.6)	1.18 (24.4)	<1
	EtOH	22.4	547	41.4	84	756	5050	1.57	-	1± 1
	MeCN	37.5	537	44.0	89	774	5700	-	-	<1

*^a^* Relative permittivity at 20 °C. *^b^* Absorption maximum. *^c^* Molar absorption coefficient. *^d^* Square transition dipole moment calculated from the measured data for the lowest-energy absorption band. *^e^* Fluorescence maximum. *^f^* Stokes shift. *^g,h^* Fluorescence lifetimes (Relative % amplitude). *^i^* Relative fluorescence quantum yield determined by optical dilution method.

**Table 3:** Calculated ground (*μ*_g_) and excited (*μ*_e_) states of D-π-A compounds **2** and **3** in solvents of varied polarity.

Cpd	Solvent	*μ*_g_	*μ*_e_ ^a^	Δ*μ*_eg_
**2**	*n*-hexane	5.08	12.57	7.49
	MCH	5.11	12.71	7.6
	toluene	5.18	13.00	7.82
	CHCl_3_	5.36	13.81	8.45
	THF	5.41	14.14	8.73
	CH_2_Cl_2_	5.43	14.23	8.8
	acetone	5.45	14.59	9.14
	EtOH	5.46	14.54	9.08
	MeCN	5.46	14.60	9.14
**3**	*n*-hexane	8.05	13.57	5.52
	MCH	8.16	13.81	5.65
	toluene	8.39	14.30	5.91
	CHCl_3_	9.16	15.97	6.81
	THF	9.49	16.72	7.23
	CH_2_Cl_2_	9.59	16.96	7.37
	acetone	9.89	17.87	7.98
	EtOH	9.92	17.76	7.84
	MeCN	9.98	17.91	7.93

^a^ Excited state dipole moments were calculated via optimization of the first excited state.

## Data Availability

Not applicable.

## Supplementary Materials

The following supporting information can be downloaded at: https://www.mdpi.com/article/10.3390/molecules27175510/s1, Figure S1: ^1^H NMR of compound **7** (400 MHz, CD_2_Cl_2_, 25 °C). Figure S2: ^13^C NMR of compound **7** (101 MHz, CD_2_Cl_2_, 25 °C). Figure S3: HRMS (ESI) spectrum of compound **7**. Figure S4: NOESY spectrum of **7**. Figure S5: ^1^H,^15^N HMBC spectrum **7**. Figure S6: ^1^H NMR of compound **2** (400 MHz, CD_2_Cl_2_, 25 °C). Figure S7: ^13^C NMR of compound **2** (101 MHz, CD_2_Cl_2_, 25 °C). Figure S8: ^11^B NMR of compound **2** (128 MHz, CD_2_Cl_2_, 25 °C). Figure S9: NOESY spectrum of **2**. Figure S10: HRMS (ESI) spectrum of **2**. Figure S11: ^1^H NMR of compound **3** (400 MHz, CD_2_Cl_2_, 25 °C). Figure S12: ^13^C NMR of compound **3** (101 MHz, CD_2_Cl_2_, 25 °C). Figure S13: ^11^B NMR of compound **3** (128 MHz, CD_2_Cl_2_, 25 °C). Figure S14: NOESY spectrum of **3**. Figure S15: HRMS (ESI) spectrum of compound **3**. Figure S16: Images of **2** in solvents of varied polarity under visible light (top), and UV light (bottom). Figure S17: Images of **3** in solvents of varied polarity under visible light (top), and UV light (bottom). Figure S18: Fluorescence decay of **2** in *n*-hexane at 298 K in air. Figure S19: Fluorescence decay of **2** in MCH at 298 K in air. Figure S20: Fluorescence decay of **2** in toluene at 298 K in air. Figure S21: Fluorescence decay of **2** in CHCl_3_ at 298 K in air. Figure S22: Fluorescence decay of **2** in THF at 298 K in air. Figure S23: Fluorescence decay of **2** in CH_2_Cl_2_ at 298 K in air. Figure S24: Fluorescence decay of **2** in acetone at 298 K in air. Figure S25: Fluorescence decay of **3** in *n*-hexane at 298 K in air. Figure S26: Fluorescence decay of **3** in MCH at 298 K in air. Figure S27: Fluorescence decay of **3** in toluene at 298 K in air. Figure S28: Fluorescence decay of **3** in CHCl_3_ at 298 K in air. Figure S29: Fluorescence decay of **3** in THF at 298 K in air. Figure S30: Fluorescence decay of **3** in CH_2_Cl_2_ at 298 K in air. Figure S31: Fluorescence decay of **3** in acetone at 298 K in air. Figure S32: Fluorescence decay of **3** in EtOH at 298 K in air. Figure S33. UV/Vis absorption spectra of **1b** in solvents of varied polarity (colors according to relative permittivity *ε*_r_: black lines for *ε*_r_ < 3; blue lines for 3 < *ε*_r_ < 10; red lines for *ε*_r_ > 10) measured at *c* = 10^˗5^ M–10^˗6^ M. Figure S34: Photoluminescence spectra of **1b** in solvents of varied polarity determined by optical dilution method (OD < 0.05). Figure S35: TD-DFT-calculated UV/Vis absorption spectrum of **7** at the CAM-B3LYP-D3(BJ)/def2-SVP (solvent CH_2_Cl_2_, PCM model) level of theory. Figure S36: TD-DFT-calculated UV/Vis absorption spectrum of **2** at the CAM-B3LYP(BJ)/def2-SVP (solvent CH_2_Cl_2_, PCM model) level of theory, Figure S37: TD-DFT-calculated UV/Vis absorption spectrum of **3** at the CAM-B3LYP-D3(BJ)/def2-SVP (solvent CH_2_Cl_2_, PCM model) level of theory. Table S1: Optical properties of A-π-A compound **1b** in solvents of varied polarity. Table S2: Optical and electrochemical properties of **2**, **3** and **7** in CH_2_Cl_2_. Table S3: TD-DFT calculated UV/vis absorption data for **7** at the CAM-B3LYP-D3(BJ)/def2-SVP (solvent CH_2_Cl_2_, PCM model) level (H = HOMO, L = LUMO, L+1 = LUMO+1, etc.), Table S4: TD-DFT-calculated UV/Vis absorption data for **2** at the CAM-B3LYP-D3(BJ)/def2-SVP (solvent CH_2_Cl_2_, PCM model) level (H = HOMO, L = LUMO, L+1 = LUMO+1, etc.). Table S5: TD-DFT-calculated UV/Vis absorption data for **3** at the CAM-B3LYP-D3(BJ)/def2-SVP (solvent CH_2_Cl_2_, PCM model) level ((H = HOMO, L = LUMO, L+1 = LUMO+1, etc.). Table S6: Onsager cavity radii. References [63,64,65,66] are cited in the Supplementary Materials.
