# Supplementary material for: Synthesis and Strong Solvatochromism of Push-Pull Thienylthiazole Boron Complexes
Source: Molecules. 2022 Aug 27;27(17):5510. doi: 10.3390/molecules27175510 (PMC9457742; doi:10.3390/molecules27175510)
Supplement: Supplementary file 1 [file molecules-27-05510-s001.zip › molecules-1869771-supplementary.pdf]

Supporting Information

Synthesis and Strong Solvatochromism of Push-Pull Thienylthiazole Boron Complexes

Martijn J. Wildervanck,[a,b] Reinhard Hecht,[b] Agnieszka Nowak-Król,[a,b]

[a] *Institut für Anorganische Chemie and Institute for Sustainable Chemistry & Catalysis with Boron, Universität Würzburg, 97074 Würzburg, Germany*
[b] *Institut für Organische Chemie and Center for Nanosystems Chemistry, Universität Würzburg, 97074 Würzburg, Germany*

E-mail: agnieszka.nowak-krol@uni-wuerzburg.de

Table of contents

1. NMR and MS Spectra 2
2. Solvent effect 9
3. Calculation of HOMO/LUMO levels and band gaps 16
4. Computations 17
5. References 99

1. NMR and MS Spectra

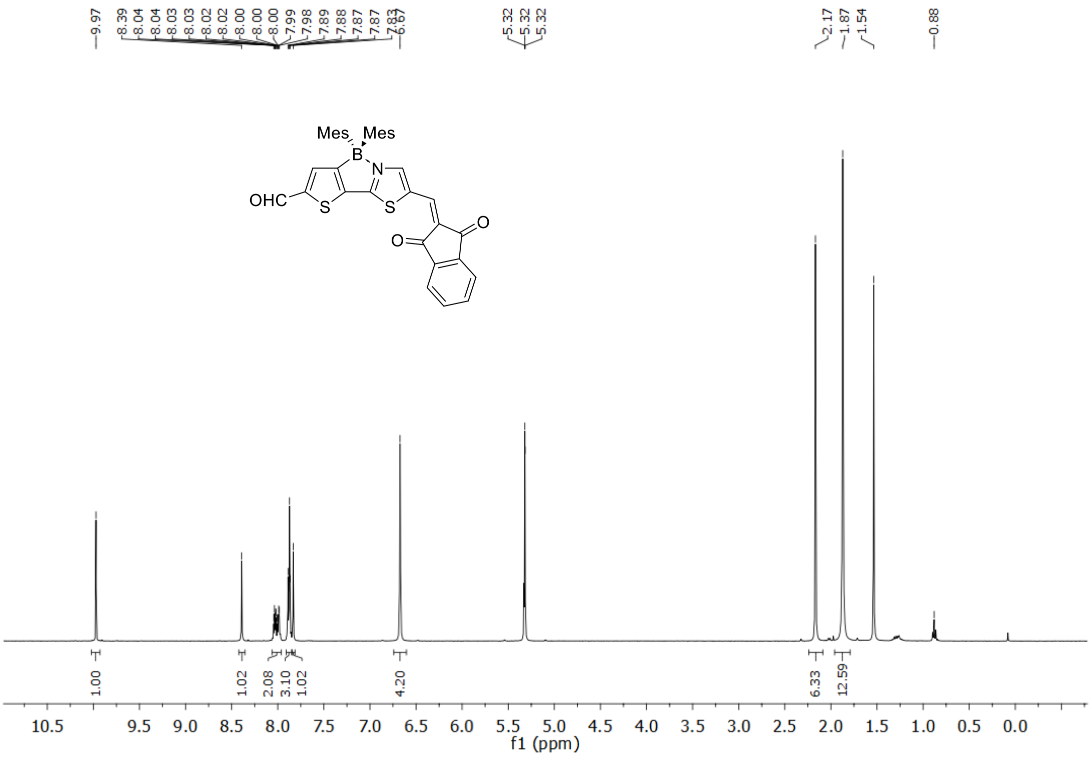

Figure S1. ^{1}H NMR of compound **7** (400 MHz, CD_2Cl_2, 25 °C).

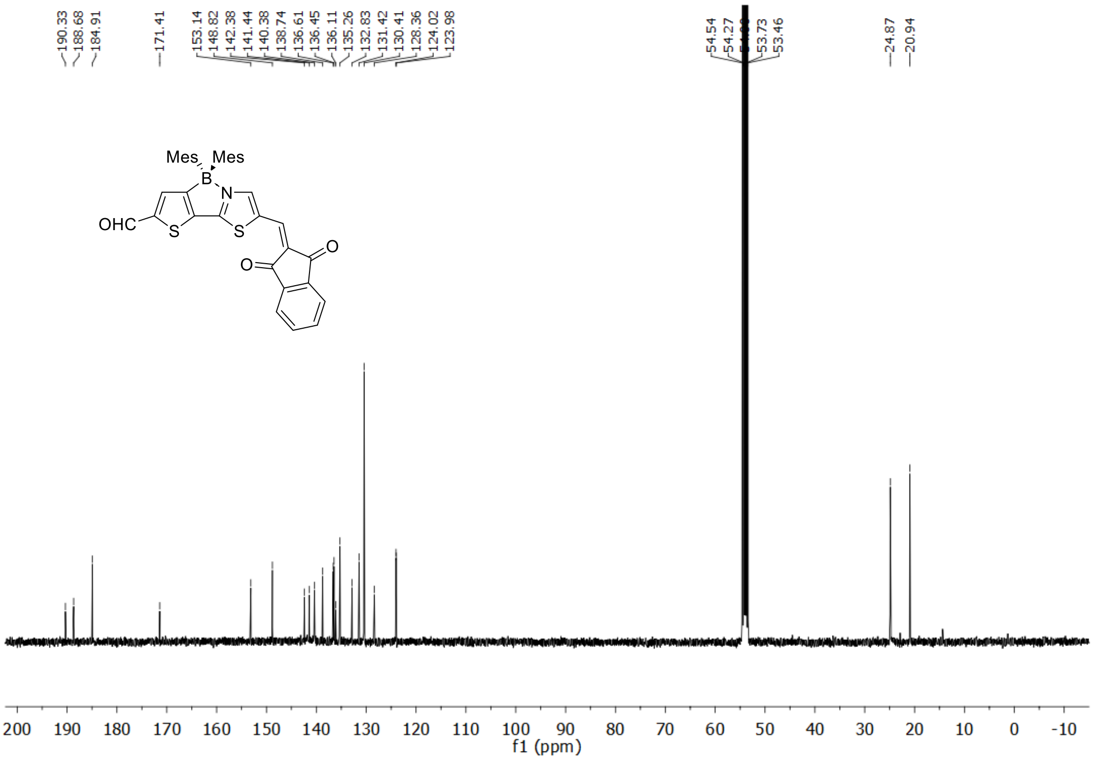

Figure S2. ^{13}C NMR of compound **7** (101 MHz, CD_2Cl_2, 25 °C).

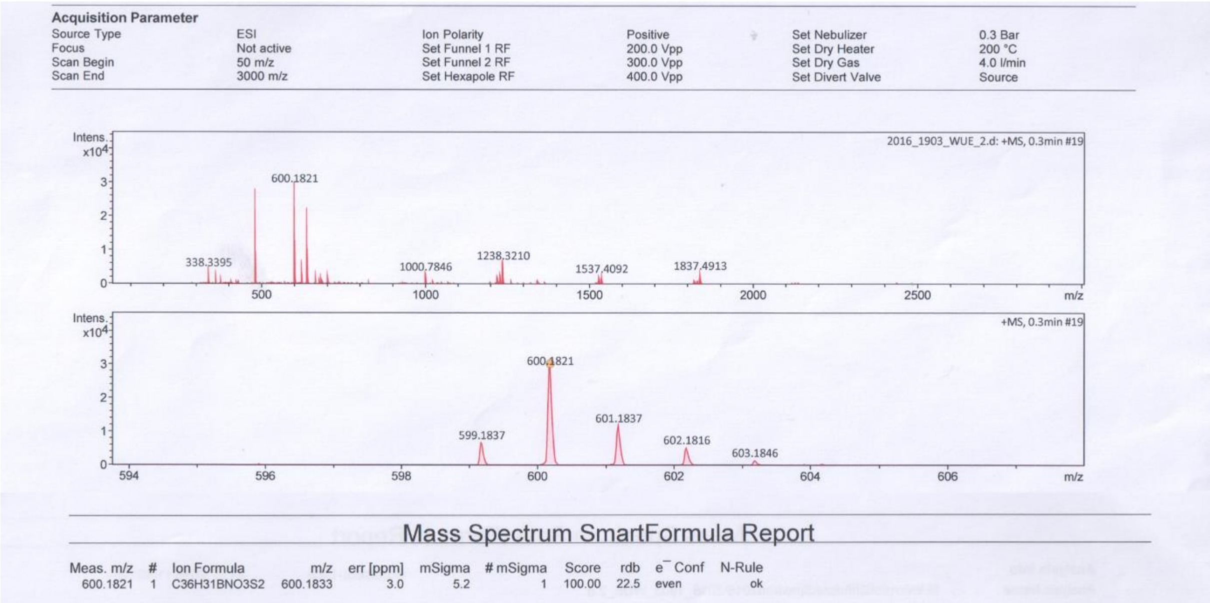

Figure S3. HRMS (ESI) spectrum of compound **7**.

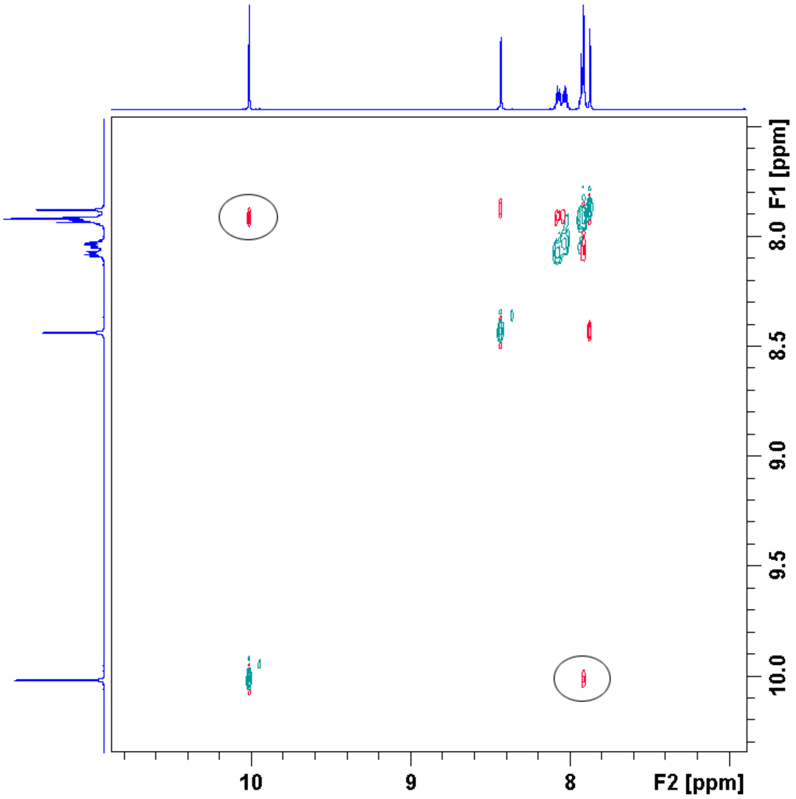

Figure S4. NOESY spectrum of **7**.

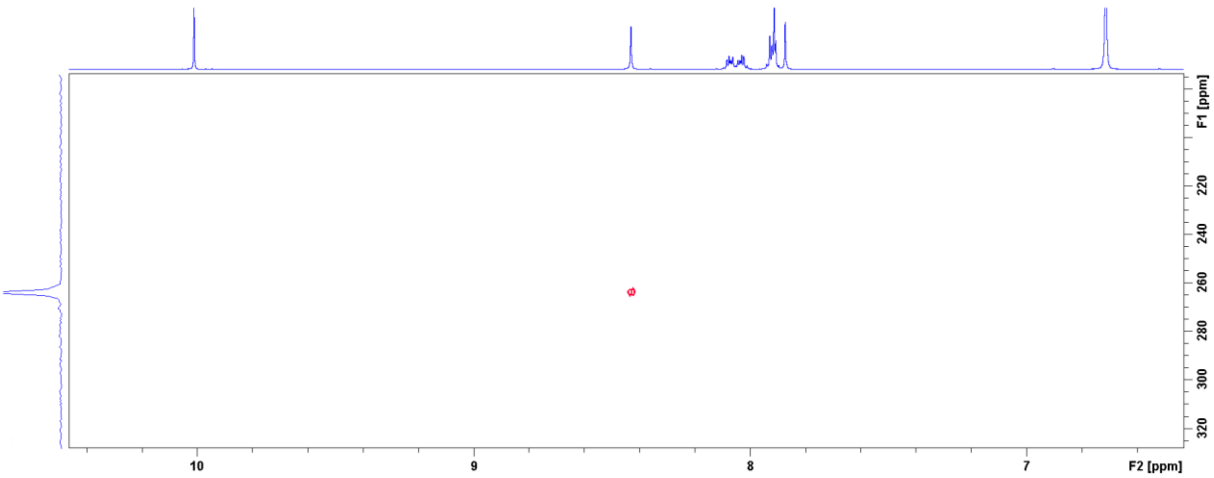

Figure S5. ^{1}H,^{15}N HMBC spectrum **7**.

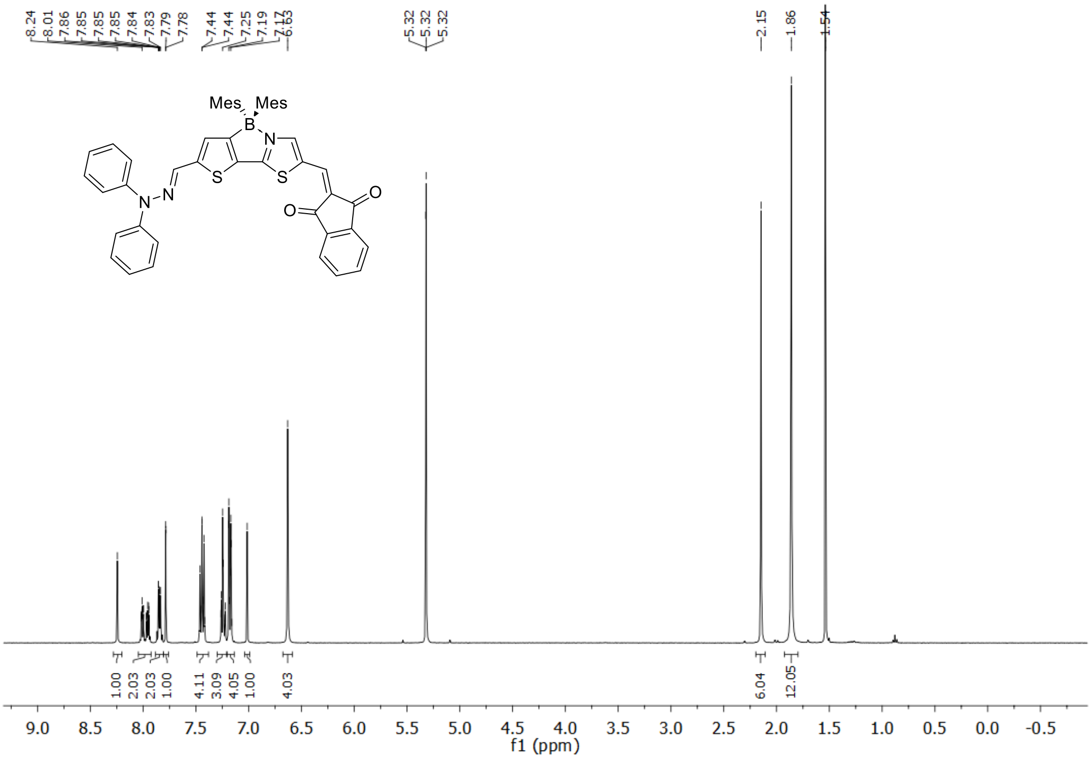

Figure S6. ^{1}H NMR of compound **2** (400 MHz, CD_2Cl_2, 25 °C).

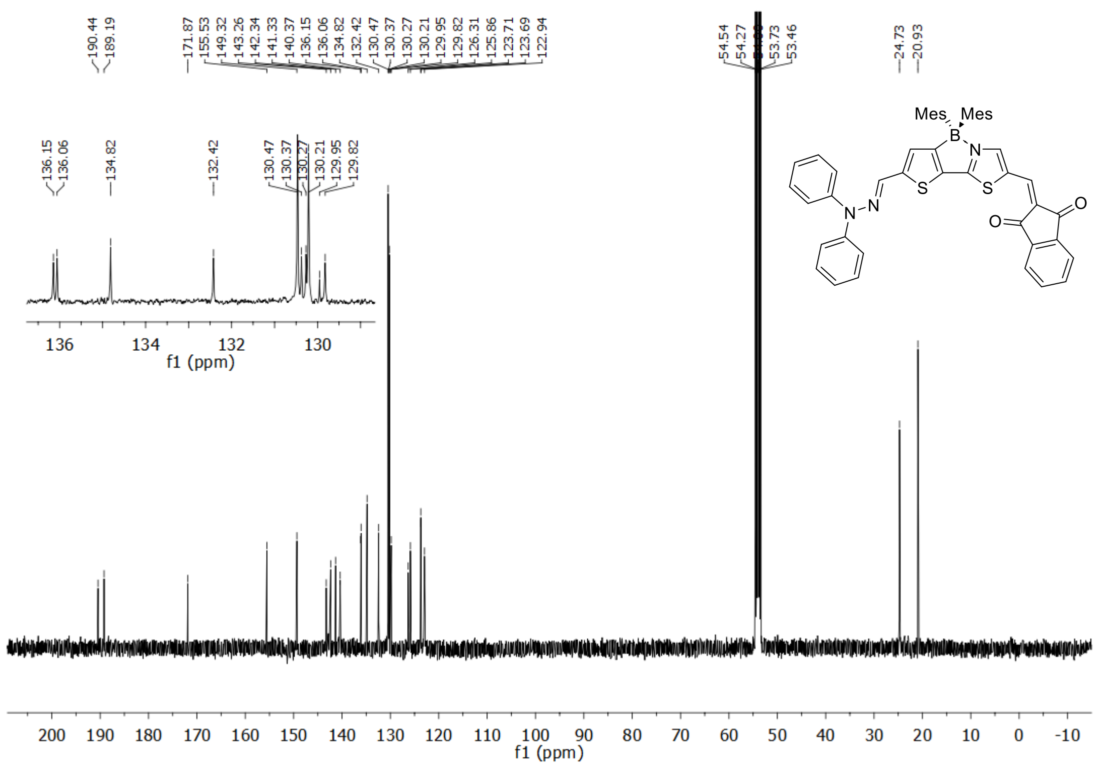

Figure S7. ^{13}C NMR of compound **2** (101 MHz, CD_2Cl_2, 25 °C).

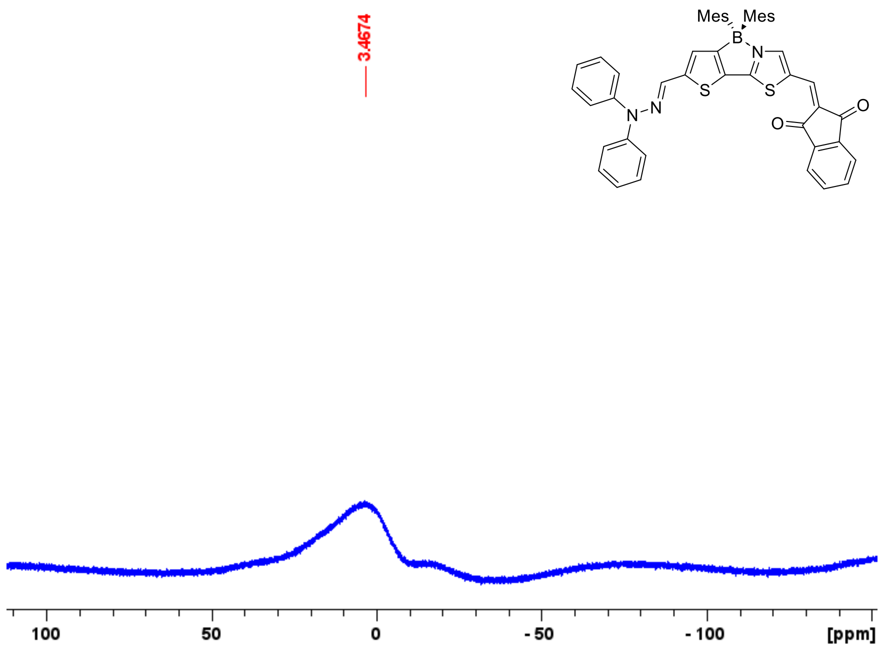

Figure S8. ^{11}B NMR of compound **2** (128 MHz, CD_2Cl_2, 25 °C).

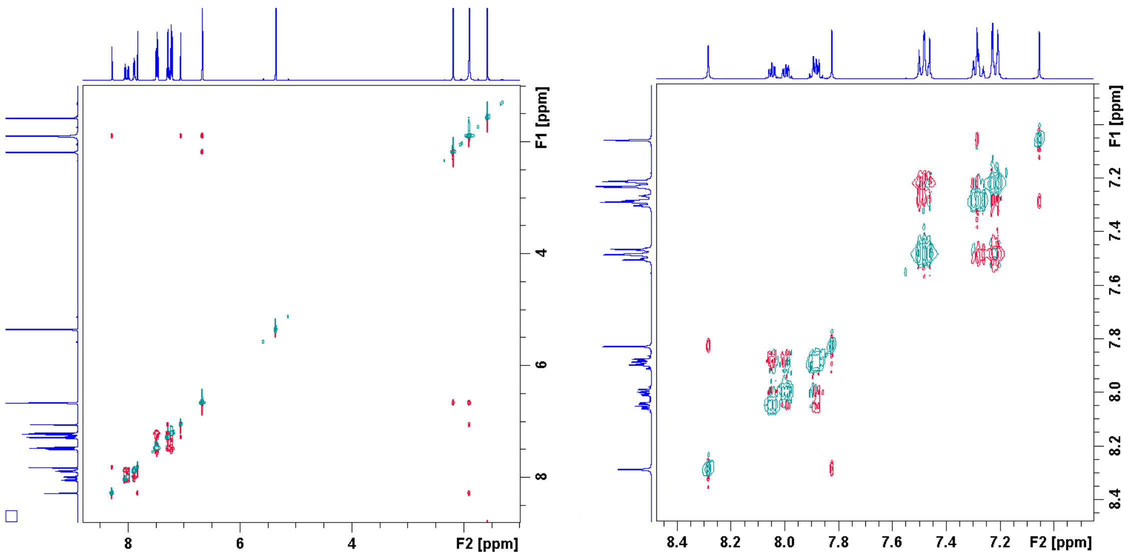

Figure S9. NOESY spectrum of **2**.

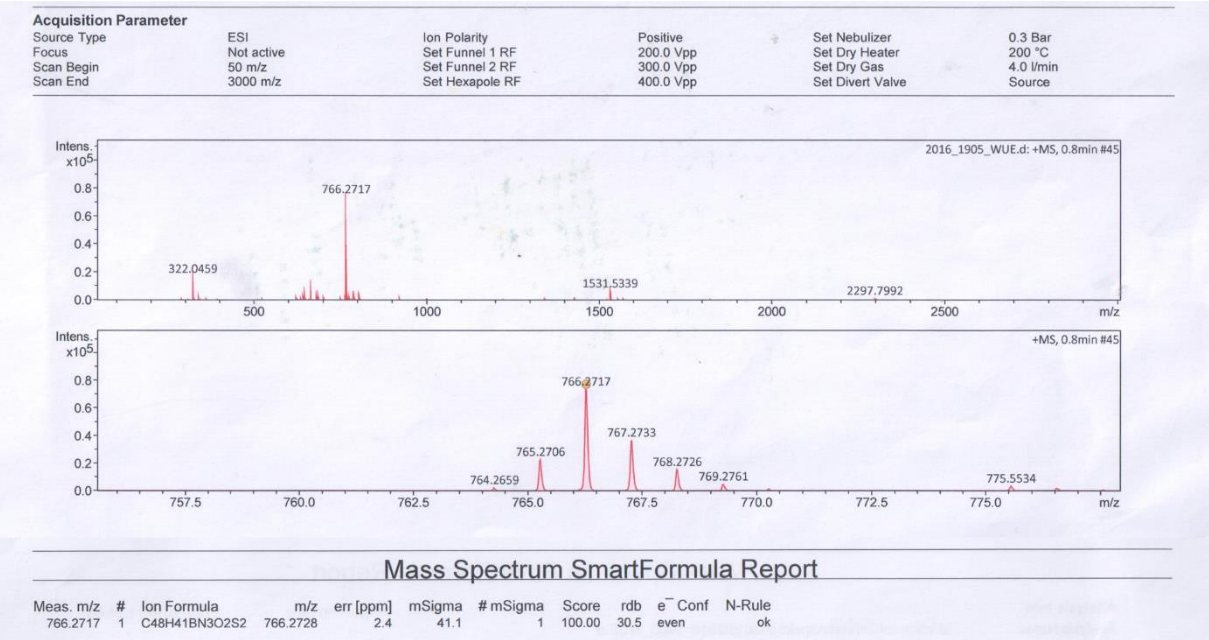

Figure S10. HRMS (ESI) spectrum of **2**.

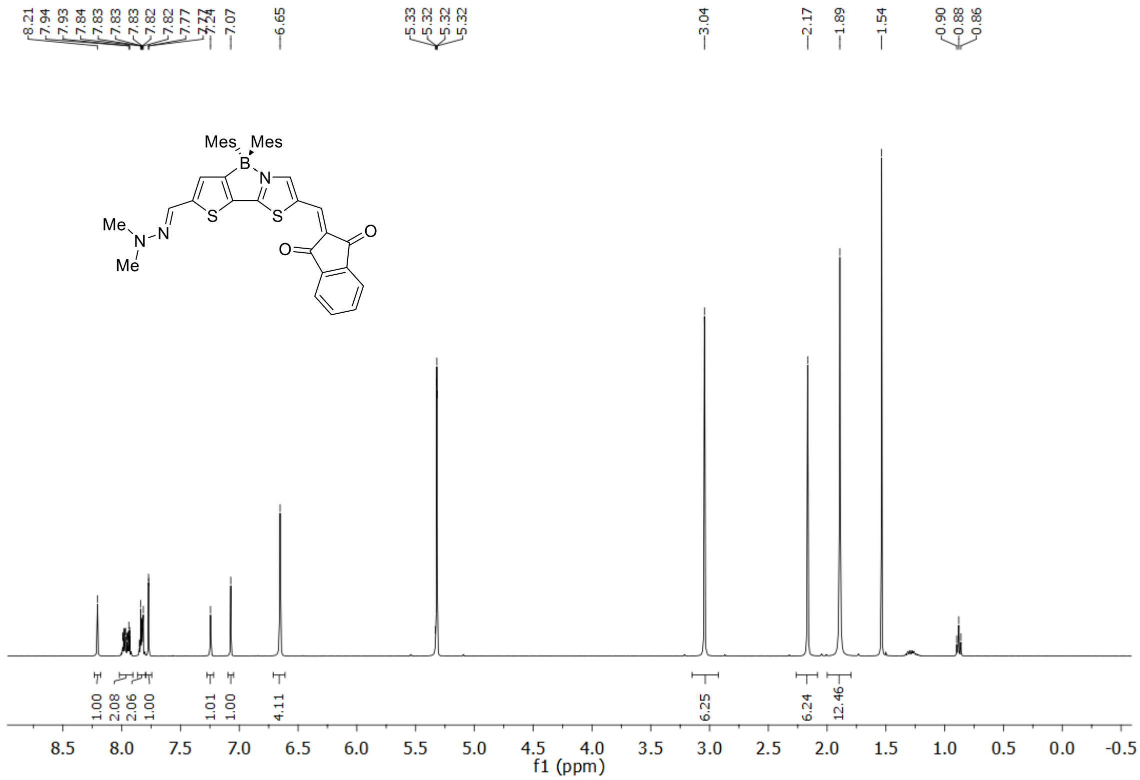

Figure S11. ^{1}H NMR of compound **3** (400 MHz, CD_2Cl_2, 25 °C).

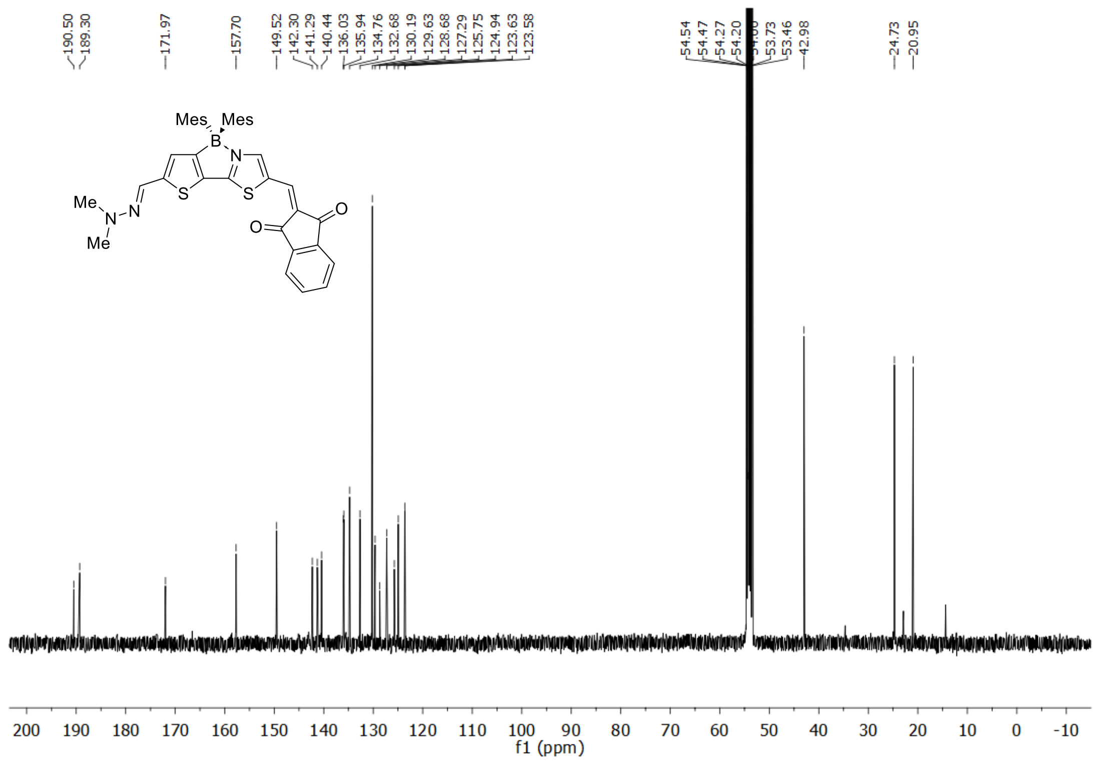

Figure S12. ^{13}C NMR of compound **3** (101 MHz, CD_2Cl_2, 25 °C).

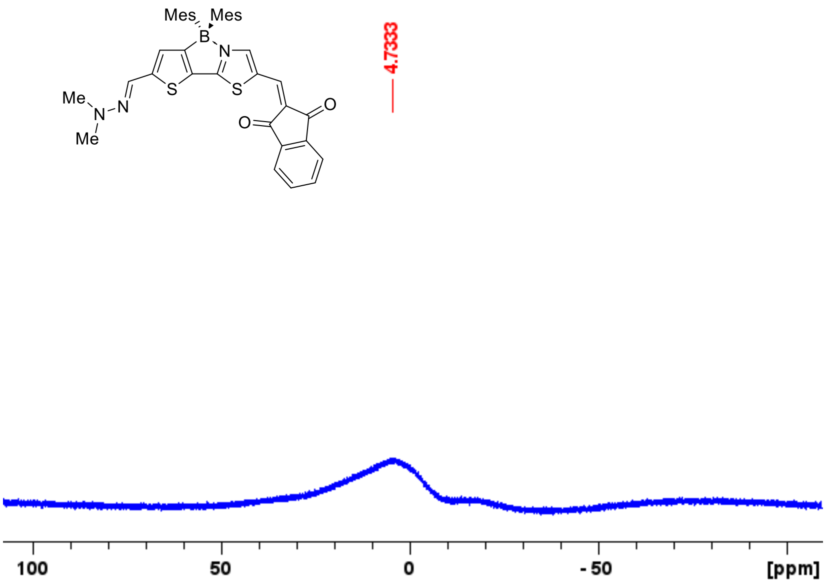

Figure S13. ^{11}B NMR of compound **3** (128 MHz, CD_2Cl_2, 25 °C).

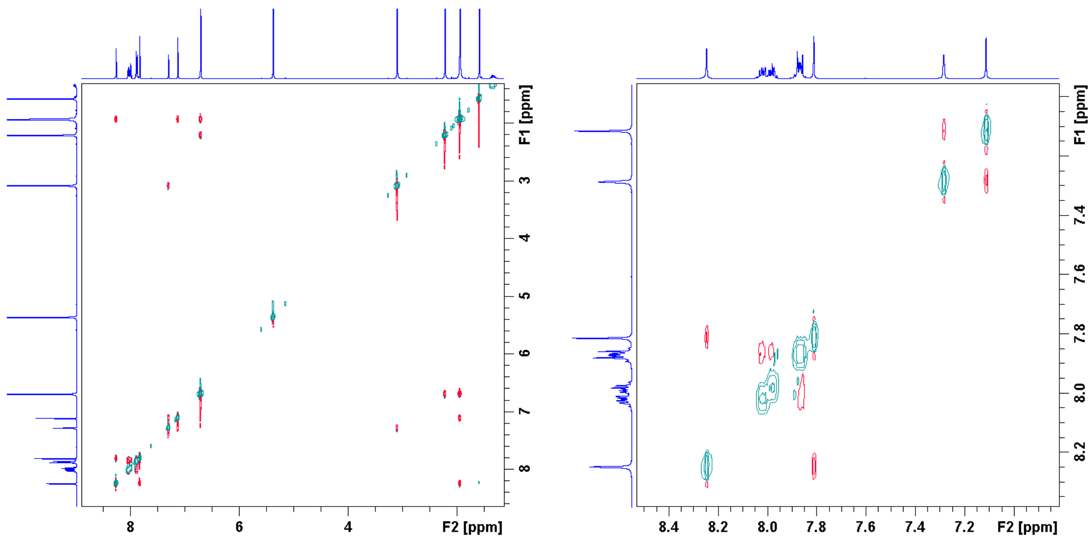

Figure S14. NOESY spectrum of **3**.

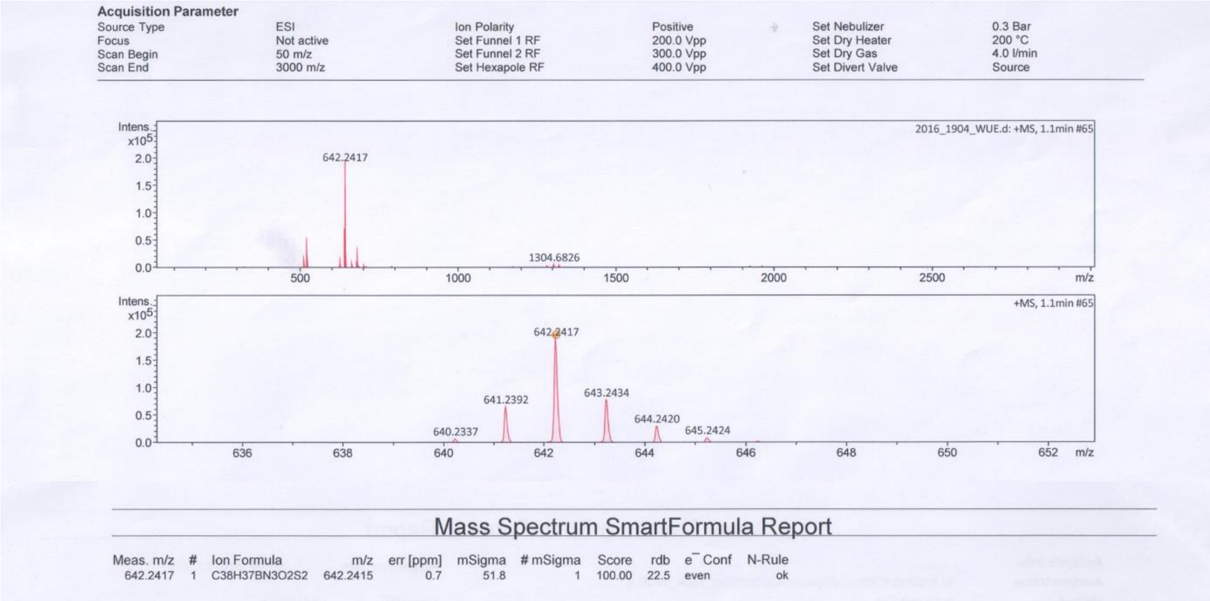

Figure S15. HRMS (ESI) spectrum of compound **3**.

2. Solvent effect

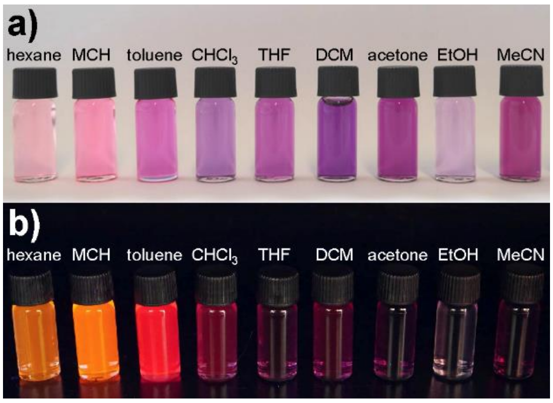

Figure S16. Images of **2** in solvents of varied polarity under visible light (top), and UV light (bottom).

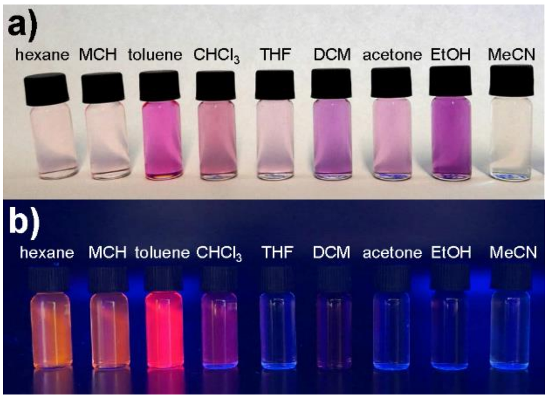

Figure S17. Images of **3** in solvents of varied polarity under visible light (top), and UV light (bottom).

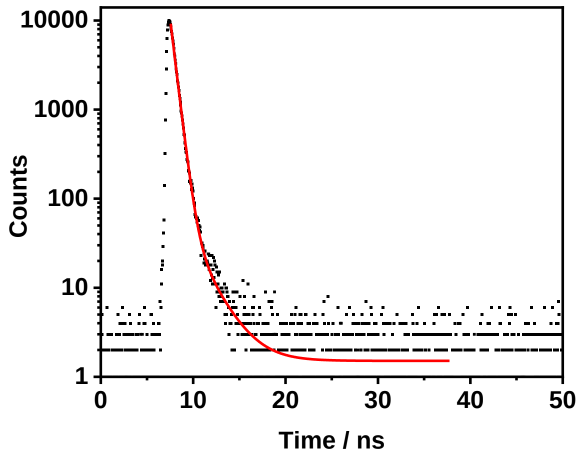

Figure S18. Fluorescence decay of **2** in *n*-hexane at 298 K in air.

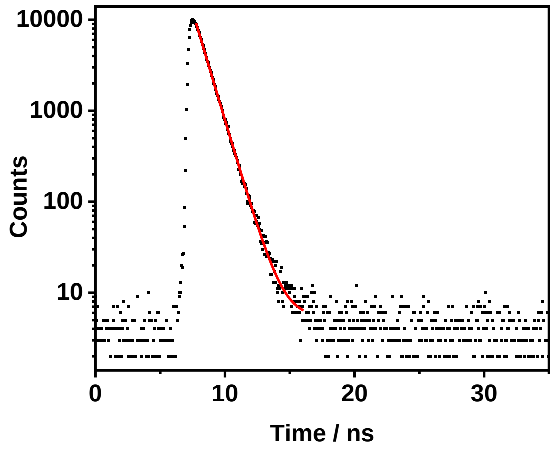

Figure S19. Fluorescence decay of **2** in MCH at 298 K in air.

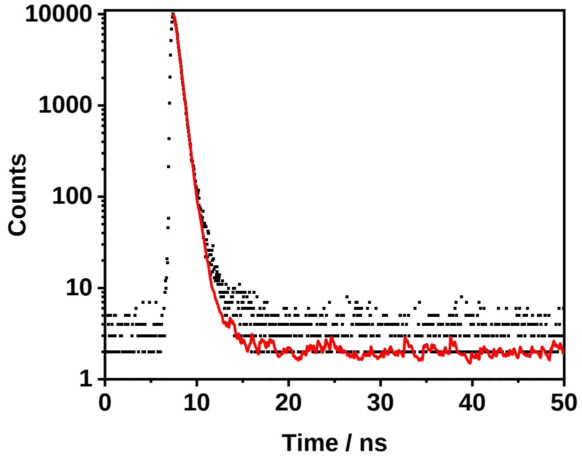

Figure S20. Fluorescence decay of **2** in toluene at 298 K in air.

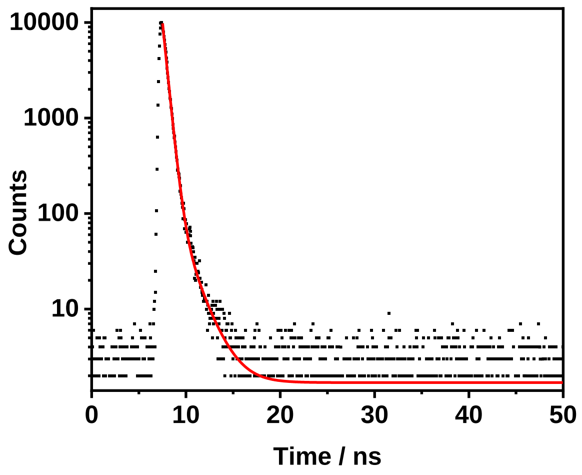

Figure S21. Fluorescence decay of **2** in $CHCl_3$ at 298 K in air.

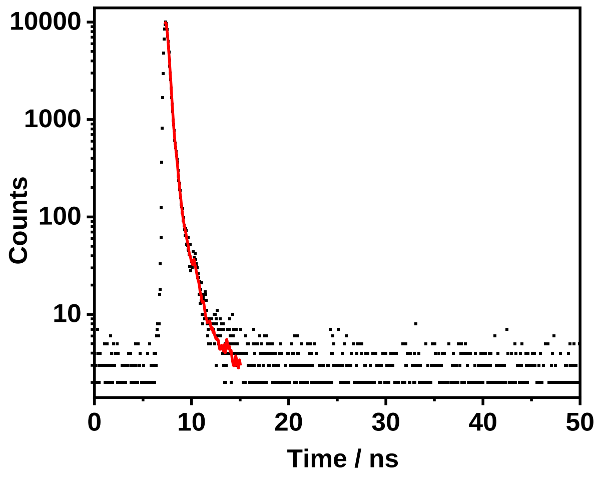

Figure S22. Fluorescence decay of **2** in THF at 298 K in air.

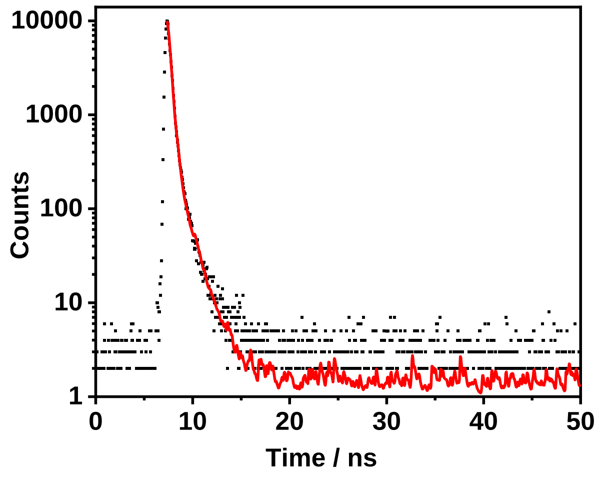

Figure S23. Fluorescence decay of **2** in CH_2Cl_2 at 298 K in air.

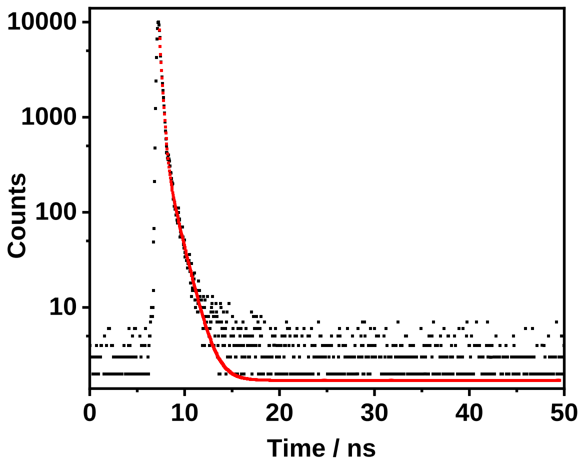

Figure S24. Fluorescence decay of **2** in acetone at 298 K in air.

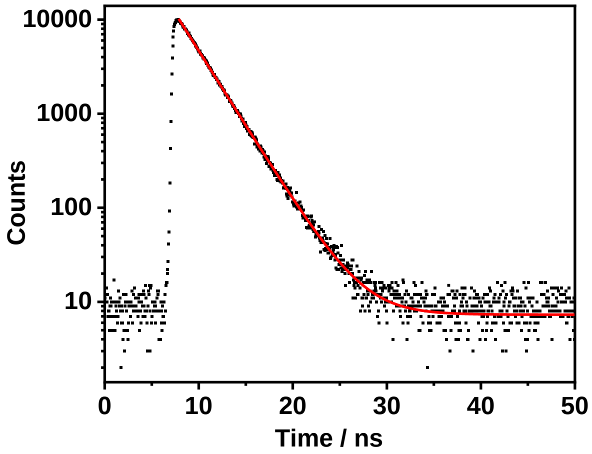

Figure S25. Fluorescence decay of **3** in *n*-hexane at 298 K in air.

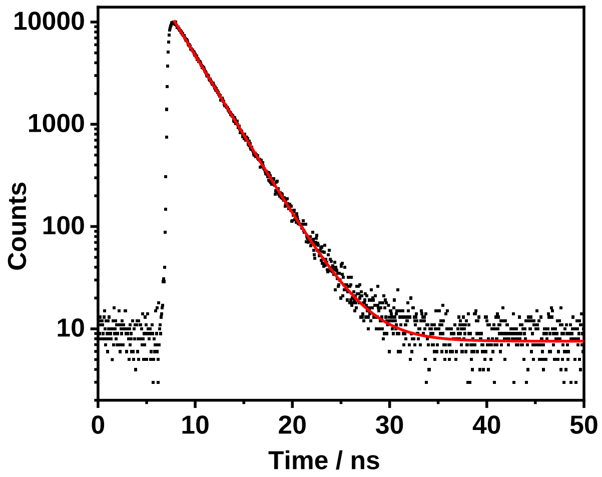

Figure S26. Fluorescence decay of **3** in MCH at 298 K in air.

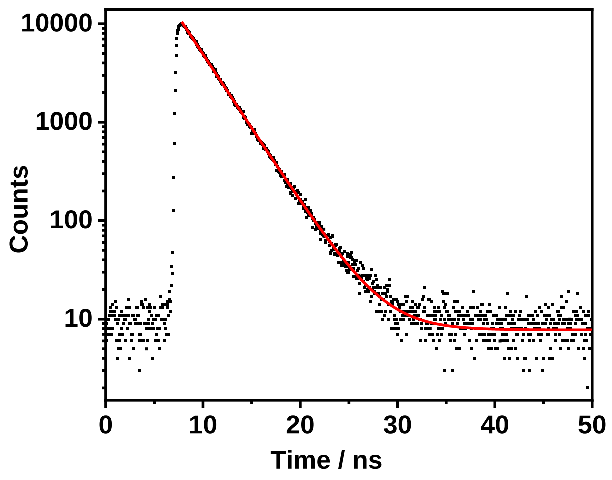

Figure S27. Fluorescence decay of **3** in toluene at 298 K in air.

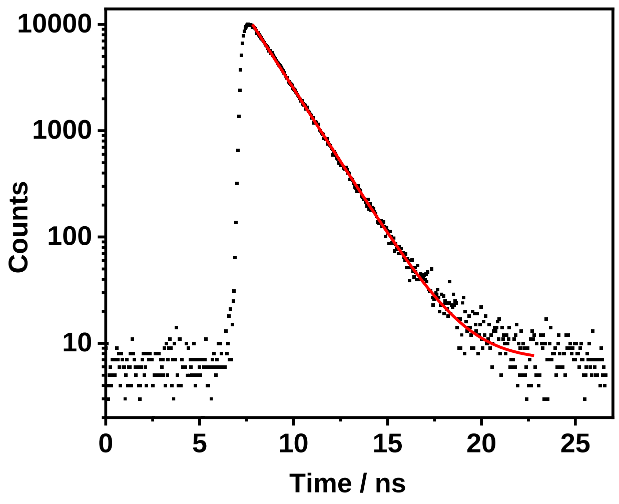

Figure S28. Fluorescence decay of **3** in $CHCl_3$ at 298 K in air.

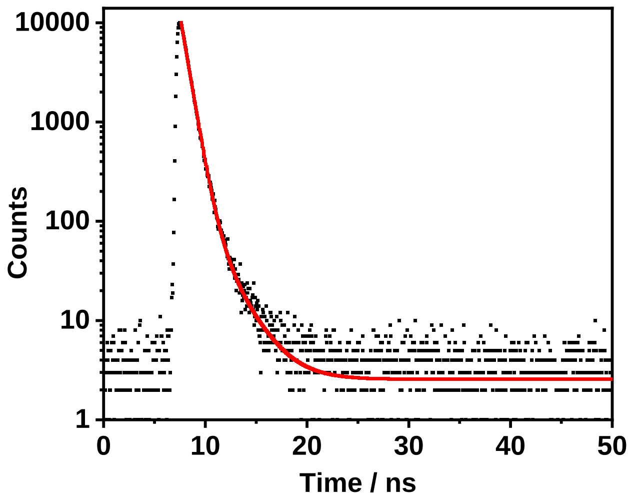

Figure S29. Fluorescence decay of **3** in THF at 298 K in air.

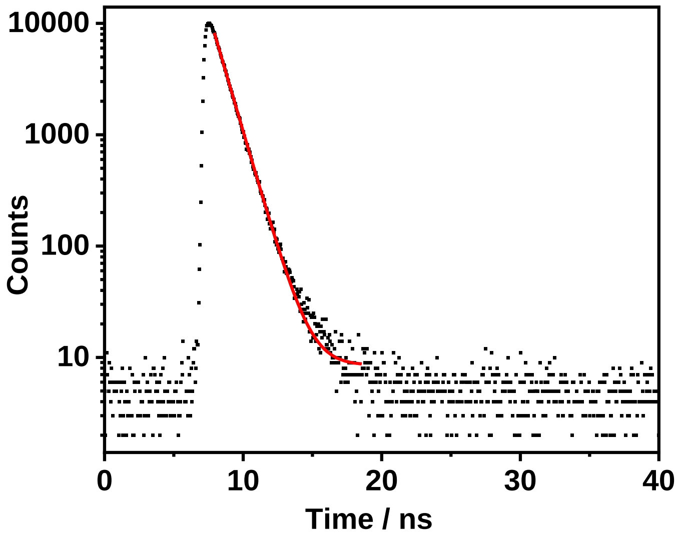

Figure S30. Fluorescence decay of **3** in CH_2Cl_2 at 298 K in air.

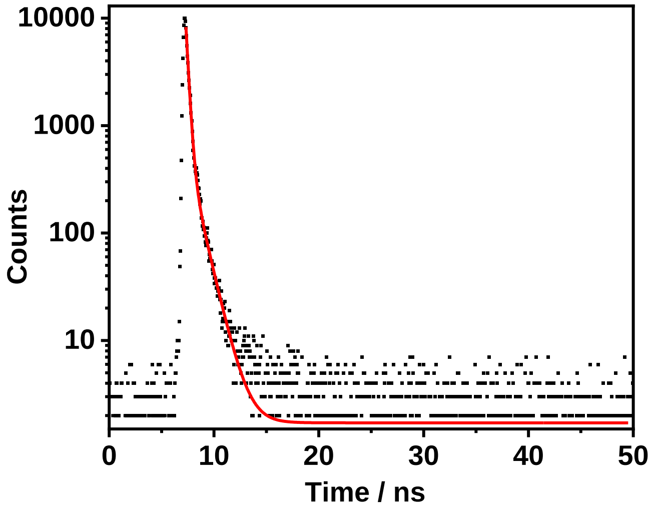

Figure S31. Fluorescence decay of **3** in acetone at 298 K in air.

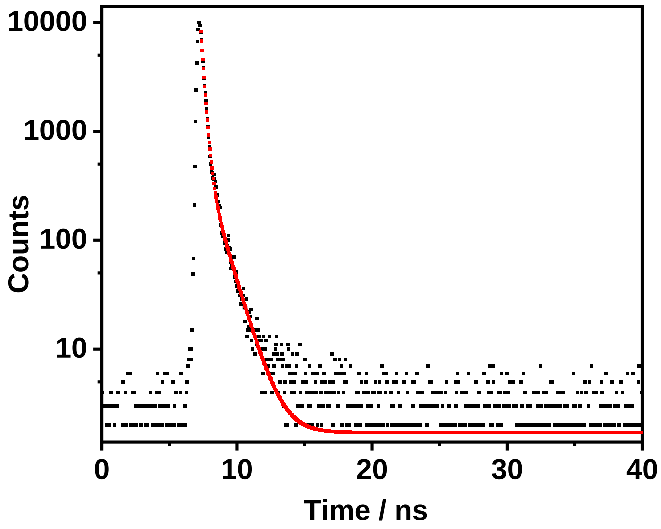

Figure S32. Fluorescence decay of **3** in EtOH at 298 K in air.

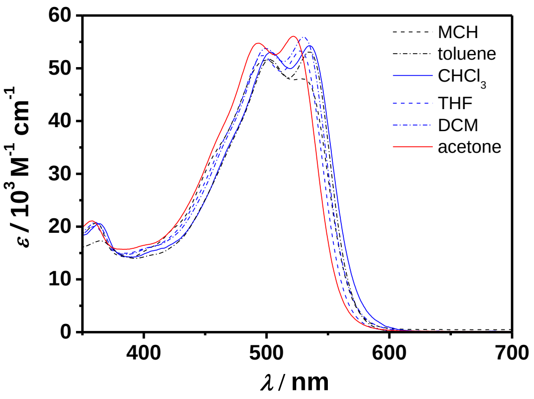

Figure S33. UV/Vis absorption of **1b** in solvents of varied polarity (colors according to relative permittivity ε_r: black lines for $\varepsilon_r < 3$; blue lines for $3 < \varepsilon_r < 10$; red lines for $\varepsilon_r > 10$) measured at $c = 10^{-5}$ M - 10^{-6} M.

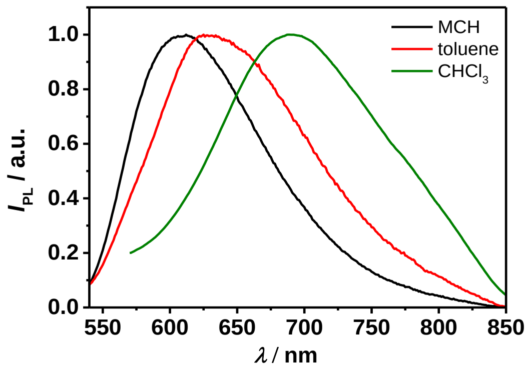

Figure S34. Photoluminescence spectra of **1b** in solvents of varied polarity determined by optical dilution method (OD < 0.05) [1].

Table S1. Optical properties of A-π-A compound **1b** in solvents of varied polarity.

Cpd	solvent	ε_r[a]	λ_{abs}[b] [nm]	ε_{max}[c] [10^3 M^{-1} cm^{-1}]	λ_{em}[d] [nm]	$\Delta\tilde{\nu}$[e] [cm^{-1}]	Φ_{fl}[f] [%]
**1b**	MCH	2.02	531<br>500	48.0<br>51.6	612	2740	6 ± 0.4
	toluene	2.38	535<br>503	53.2<br>51.7	625	3240	4 ± 0.3
	$CHCl_3$	4.8	535<br>504	54.3<br>53.0	688	4470	< 1
	THF	7.6	528<br>497	53.3<br>52.4	–[g]	–	–
	DCM	9.1	530<br>499	55.9<br>53.8	–[g]	–	–
	acetone	20.6	522<br>494	56.0<br>54.8	–[g]	–	–

[a]Relative permittivity at 20°C. [b]Absorption maximum and a vibronic progression. [c]Molar absorption coefficient. [d]Fluorescence maximum. [e]Stokes shift. [f]Relative fluorescence quantum yield determined by optical dilution method. [g]Non-fluorescent.

3. Calculation of HOMO/LUMO levels and band gaps

The HOMO/LUMO energy levels were calculated according to the following equations:

$$E_{HOMO} = -e\, E_{1/2}^{ox} - 5.15 \text{ eV.} \quad \text{(eq. S1) [2]}$$

$$E_{LUMO} = -e\, E_{1/2}^{red} - 5.15 \text{ eV} \quad \text{(eq. S2)}$$

The electrochemical band gaps were calculated according to the following equation:

$$E_g^{CV} = E_{1/2}^{ox} - E_{1/2}^{red} \quad \text{(eq. S3)}$$

Table S2. Optical and electrochemical properties of **2**, **3**, and **7** in CH_2Cl_2.

Dye	λ_{abs}[a] [nm]	$E_{1/2}^{red}$[b] [V]	$E_{1/2}^{ox}$[b] [V]	E_g^{CV}[d] [eV]	E_{HOMO} [eV]	E_{LUMO} [eV]
**2**	571	–1.32[c]	+0.65[c]	1.97	–5.80	–3.83
**3**	556	–1.36[c]	+0.51[c]	1.87	–5.66	–3.79
**7**	432	–1.21[c]	+1.08[c]	2.29	–6.23	–3.94

[a]Absorption maximum. [b]Redox potentials *vs.* Fc^+/Fc in CH_2Cl_2 (*c* ~ 2.5); scan rate: 100 mV s^{-1}; supporting electrolyte: Bu_4NPF_6 (0.1 M). [c]Peak potential. [d]Electrochemical band gap.

4. Computations

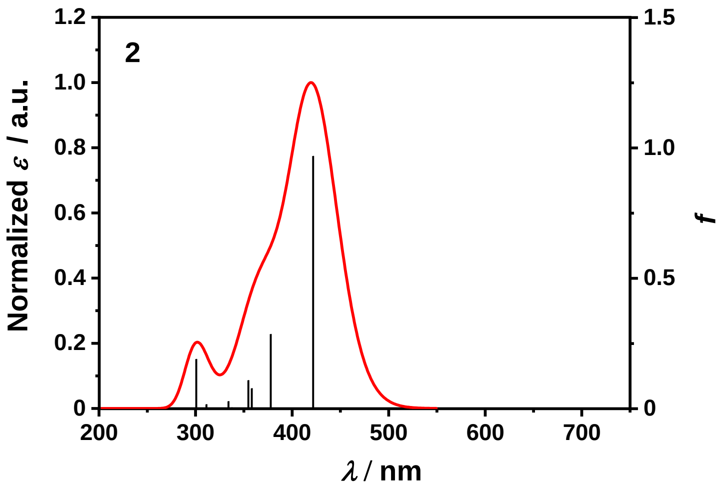

Figure S35. TD-DFT-calculated UV/Vis absorption spectrum of **7** at the CAM-B3LYP-D3(BJ)/def2-SVP (solvent CH_2Cl_2, PCM model) level of theory.

Table S3. TD-DFT calculated UV/vis absorption data for **7** at the CAM-B3LYP-D3(BJ)/def2-SVP (solvent CH_2Cl_2, PCM model) level (H = HOMO, L= LUMO, L+1 = LUMO+1, etc.).

	Calcd λ [nm]	Oscillator strength (*f*)	Composition	Coefficient	Contribution
1	421.7	0.9692	H-5 -> L	-0.24933	12%
			H-3 -> L	0.16464	5%
			H-2 -> L	-0.1921	7%
			H -> L	0.5774	67%
			H -> L+1	0.1047	2%
2	377.77	0.2864	H-5 -> L	0.30309	18%
			H-3 -> L	-0.21855	10%
			H-2 -> L	0.43906	39%
			H -> L	0.32444	21%
			H -> L+1	0.12708	3%
3	358.22	0.0782	H-13 -> L+1	0.11274	3%
			H-13 -> L+2	-0.16638	6%
			H-7 -> L	0.45041	41%
			H-7 -> L+1	-0.23923	11%
			H-5 -> L	0.22596	10%
			H-3 -> L	-0.16944	6%
			H-2 -> L	-0.27476	15%
4	354.61	0.1093	H-13 -> L+2	-0.12905	3%
			H-7 -> L	0.3253	21%
			H-7 -> L+1	-0.15703	5%
			H-5 -> L	-0.27427	15%
			H-3 -> L	0.22851	10%
			H-2 -> L	0.40628	33%
5	337.41	0.0047	H-11 -> L	0.10801	2%
			H-10 -> L	0.42366	36%

			H-10	-> L+1	0.33016	22%
			H-10	-> L+2	0.18517	7%
			H-10	-> L+3	-0.26033	14%
			H-10	-> L+4	-0.10274	2%
			H-10	-> L+5	-0.10056	2%
			H-3	-> L	0.11606	3%
6	336.37	0.0001	H-13	-> L	-0.29983	18%
			H-13	-> L+1	0.15856	5%
			H-13	-> L+2	0.1125	3%
			H-7	-> L+1	-0.24059	12%
			H-7	-> L+2	0.50504	51%
			H-7	-> L+12	-0.13446	4%
7	334.05	0.0288	H-5	-> L	0.39468	31%
			H-3	-> L	0.54202	59%
8	321.04	0.0022	H-5	-> L	0.1612	5%
			H-4	-> L	0.66933	90%
9	311.19	0.017	H-6	-> L	0.44342	39%
			H-6	-> L+1	0.11114	2%
			H-5	-> L+1	0.21375	9%
			H-3	-> L+1	-0.1514	5%
			H-2	-> L+1	0.14266	4%
			H	-> L	0.1183	3%
			H	-> L+1	-0.34022	23%
			H	-> L+2	-0.11195	3%
10	300.68	0.1904	H-8	-> L	0.15153	5%
			H-6	-> L	0.45766	42%
			H-6	-> L+1	0.17264	6%
			H-5	-> L+1	-0.1999	8%
			H-3	-> L+1	0.15616	5%
			H-2	-> L+1	-0.11377	3%
			H	-> L+1	0.33111	22%

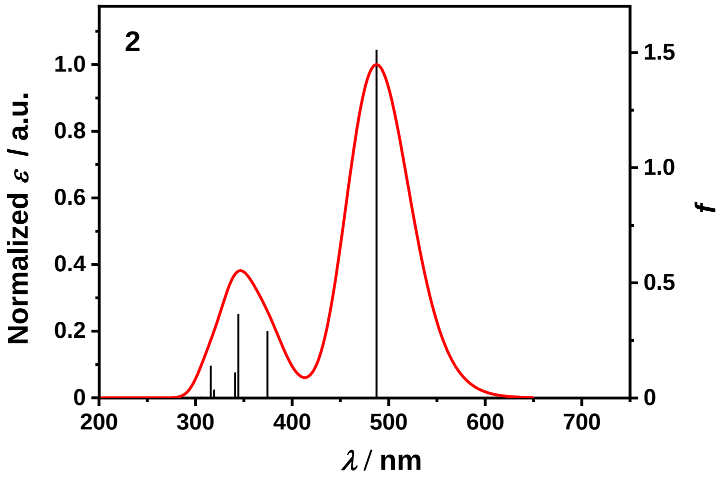

Figure S36. TD-DFT-calculated UV/Vis absorption spectrum of **2** at the CAM-B3LYP(BJ)/def2-SVP (solvent CH_2Cl_2, PCM model) level of theory.

Table S4. TD-DFT-calculated UV/Vis absorption data for **2** at the CAM-B3LYP-D3(BJ)/def2-SVP (solvent CH_2Cl_2, PCM model) level (H = HOMO, L= LUMO, L+1 = LUMO+1, etc.).

	Calcd λ (nm)	Oscillator strength (*f*)	Composition	Coefficient	Contribution
1	487.32	1.5114	H-5 -> L	0.1586	5%
			H -> L	0.63379	80%
			H -> L+2	0.165	5%
2	374.34	0.2907	H-1 -> L	0.64636	84%
			H-1 -> L+2	0.15569	5%
			H -> L+2	0.10823	2%
3	352.12	0.0039	H-15 -> L+1	-0.23165	11%
			H-15 -> L+2	0.10904	2%
			H-9 -> L	0.54728	60%
			H-9 -> L+1	-0.20403	8%
			H-9 -> L+2	-0.17908	6%
			H-2 -> L	-0.1452	4%
4	344.16	0.3648	H-5 -> L	-0.19312	7%
			H-3 -> L	0.18095	7%
			H-2 -> L	-0.31889	20%
			H -> L+1	0.31442	20%
			H -> L+2	0.36544	27%
			H -> L+3	-0.10482	2%
5	340.89	0.1108	H-5 -> L	-0.19329	7%
			H-2 -> L	0.56569	64%
			H -> L+1	0.16783	6%
			H -> L+2	0.17454	6%
6	335.31	0.0008	H-15 -> L	0.29519	17%
			H-15 -> L+2	-0.16418	5%
			H-9 -> L+1	-0.46724	44%
			H-9 -> L+2	0.31435	20%
			H-9 -> L+3	-0.13284	4%
			H-2 -> L	0.12911	3%
7	319.15	0.0366	H-5 -> L	-0.10978	2%

			H-3 -> L	0.58992	70%
			H -> L	0.14226	4%
			H -> L+1	-0.21444	9%
8	315.75	0.1406	H-5 -> L	0.48189	46%
			H-5 -> L+1	0.12823	3%
			H-4 -> L	-0.26049	14%
			H-3 -> L	0.26805	14%
			H -> L	-0.15962	5%
			H -> L+1	0.17727	6%
9	308.77	0.0001	H-11 -> L+1	0.10121	2%
			H-8 -> L+1	0.17171	6%
			H-5 -> L+1	-0.20246	8%
			H-5 -> L+2	0.18042	7%
			H -> L+1	-0.32871	22%
			H -> L+2	0.40258	32%
10	305.12	0.0056	H-5 -> L	0.29278	17%
			H-4 -> L	0.6212	77%

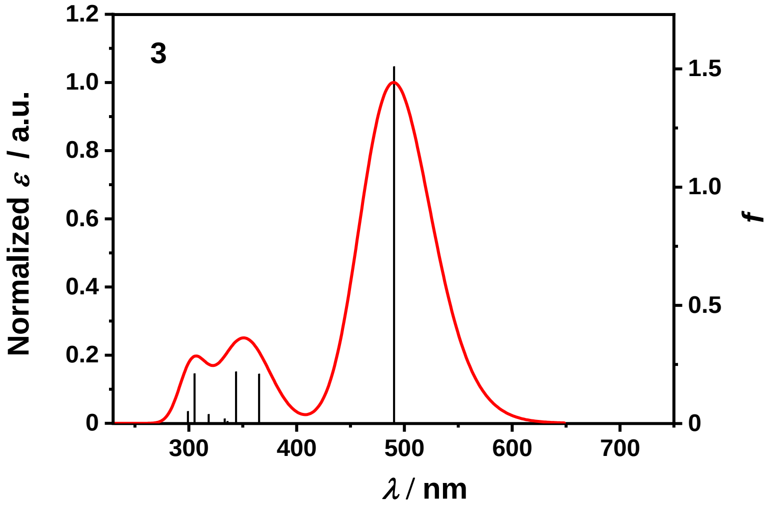

Figure S37. TD-DFT-calculated UV/Vis absorption spectrum of **3** at the CAM-B3LYP-D3(BJ)/def2-SVP (solvent CH_2Cl_2, PCM model) level of theory.

Table S5. TD-DFT-calculated UV/Vis absorption data for **3** at the CAM-B3LYP-D3(BJ)/def2-SVP (solvent CH_2Cl_2, PCM model) level (H = HOMO, L= LUMO, L+1 = LUMO+1, etc.).

	Calcd *λ* (nm)	Oscillator strength (*f*)	Composition	Coefficient	Contribution
1	490.38	1.5108	H-5 -> L	-0.14046	4%
			H -> L	0.65857	87%
			H -> L+2	-0.15895	5%
2	365.11	0.2108	H-1 -> L	0.64594	83%
			H-1 -> L+2	-0.18699	7%
			H -> L+2	-0.11932	3%
3	349.38	0.0029	H-12 -> L+1	0.26208	14%
			H-7 -> L	0.5576	62%
			H-7 -> L+1	-0.16435	5%
			H-7 -> L+2	0.20091	8%
4	343.76	0.2205	H-5 -> L	-0.17166	6%

			H-5 -> L+1	0.10394	2%
			H-1 -> L	0.10053	2%
			H -> L	0.10771	2%
			H -> L+1	-0.32839	22%
			H -> L+2	0.52313	55%
5	335.85	0.0104	H-12 -> L	0.25686	13%
			H-12 -> L+2	0.14476	4%
			H-7 -> L	0.1324	4%
			H-7 -> L+1	0.43467	38%
			H-7 -> L+2	0.18285	7%
			H-7 -> L+3	0.1158	3%
			H-2 -> L	0.36248	26%
6	333.31	0.0218	H-12 -> L	-0.15871	5%
			H-7 -> L+1	-0.31745	20%
			H-3 -> L	-0.10053	2%
			H-2 -> L	0.55844	62%
7	318.34	0.0399	H-8 -> L+1	-0.13369	4%
			H-5 -> L	-0.10322	2%
			H-5 -> L+1	-0.28033	16%
			H -> L+1	0.49212	48%
			H -> L+2	0.297	18%
8	311.77	0.004	H-6 -> L	-0.11367	3%
			H-3 -> L	0.6553	86%
			H-3 -> L+2	-0.11226	3%
			H-2 -> L	0.10944	2%
			H -> L+2	0.11316	3%
9	305.25	0.2125	H-6 -> L	-0.12111	3%
			H-5 -> L	0.52958	56%
			H-4 -> L	0.31319	20%
			H -> L	0.16667	6%
			H -> L+2	0.16081	5%
10	299.1	0.0528	H-6 -> L	0.58665	69%
			H-6 -> L+2	-0.20539	8%
			H-4 -> L	0.19647	8%
			H-3 -> L	0.11683	3%
			H-1 -> L	-0.11952	3%

The gas-phase molecular volumes V_M, defined as the space occupied by the 0.001-au electron density envelope [3], was obtained by DFT calculations on gas-phase optimized geometries using B3LYP [4–6] as a functional and def2-SVP [7] as a basis set. Assuming the solute cavity to be a sphere, the Onsager cavity radii *a* were estimated from the following relationship:

$$a = \sqrt[3]{\frac{3V_{\mathrm{M}}}{4\pi}}$$

The estimated Onsager cavity radii are presented in Table S6.

Table S6. Onsager cavity radii.

Compound	*a* [Å]
**2**	6.23
**3**	5.85

Cartesian coordinates of 2 (B3LYP-D3(BJ)/def2-SVP, *n*-hexane, PCM model) in S_0 state

E = -2987.6218 Hartrees

Symbol	X	Y	Z
C	0.73994	-1.07703	-0.18631
N	-1.664468	-0.98082	0.069095
S	-2.337327	1.515586	0.057942
O	-4.290013	3.461249	0.119988
B	-0.498261	-2.1302	-0.0305
C	-5.415146	2.986371	0.131397
S	1.502386	1.463178	-0.05613
O	-7.960064	0.455207	0.148687
C	-6.693562	3.76143	0.155793
C	-6.881937	5.141211	0.169514
H	-6.023874	5.816257	0.1625
C	-8.196611	5.621673	0.192203
H	-8.379836	6.698621	0.203396
C	-9.289333	4.737983	0.200788
H	-10.30392	5.142747	0.218482
C	-9.09496	3.351541	0.186918
H	-9.935035	2.654157	0.19321
C	-7.785769	2.878114	0.164371
C	-7.286437	1.468174	0.145913
C	-5.798843	1.559381	0.124124
C	-3.008872	-0.98975	0.062571
H	-3.550898	-1.93362	0.048657
C	-3.606072	0.264283	0.076814
C	-5.017059	0.442332	0.100544
H	-5.580067	-0.50008	0.099347
C	-1.142613	0.271585	0.026459
C	0.263252	0.240372	-0.06902
C	2.153227	-1.07524	-0.22623
H	2.775177	-1.96862	-0.28866
C	2.714554	0.198545	-0.18226
C	4.116015	0.524741	-0.22527
H	4.819252	-0.31642	-0.28792
C	-1.034513	-3.07556	-1.25113
C	-0.710262	-2.82803	-2.61201
C	-1.225738	-3.64325	-3.6283
H	-0.950524	-3.42944	-4.6659
C	-2.090858	-4.70873	-3.36245
C	-2.446695	-4.92248	-2.03094

H	-3.143342	-5.73201	-1.79165
C	-1.942871	-4.13362	-0.98303
C	0.182527	-1.68599	-3.04315
C	-2.611782	-5.58551	-4.47297
C	-2.412641	-4.5055	0.409911
C	-0.112714	-2.89488	1.369624
C	0.779791	-3.99804	1.265259
C	1.202496	-4.68424	2.411221
H	1.884506	-5.53267	2.29406
C	0.784727	-4.32129	3.694174
C	-0.054656	-3.2114	3.797364
H	-0.374985	-2.88029	4.790027
C	-0.493809	-2.4914	2.674661
C	-1.364858	-1.286	2.953995
C	1.299473	-4.51783	-0.05959
C	1.218857	-5.10064	4.909668
N	4.491066	1.7625	-0.18575
N	5.778511	2.110296	-0.21448
C	6.814021	1.118825	-0.28264
C	8.786748	-0.84538	-0.41576
C	7.323627	0.56813	0.897532
C	7.28087	0.689674	-1.52904
C	8.271125	-0.29341	-1.59278
C	8.313034	-0.41531	0.827866
C	6.093633	3.486655	-0.17775
C	6.735838	6.225292	-0.10495
C	7.437102	3.901602	-0.18786
C	5.072218	4.453192	-0.13064
C	5.400434	5.806951	-0.09458
C	7.74649	5.26244	-0.1518
H	8.238472	3.164298	-0.22327
H	8.79551	5.568321	-0.16012
H	6.983923	7.288309	-0.07683
H	4.596522	6.546407	-0.05817
H	4.03438	4.1262	-0.12285
H	6.942475	0.917768	1.858846
H	8.716147	-0.84574	1.74716
H	9.561255	-1.61387	-0.46778
H	8.641731	-0.62854	-2.56401
H	6.867176	1.133505	-2.43653
H	0.238366	-1.63171	-4.14017
H	1.20862	-1.79469	-2.6617
H	-0.180992	-0.71408	-2.67886
H	1.506887	-3.72348	-0.78627
H	0.567911	-5.18285	-0.54511
H	-3.342452	-5.09136	0.354421

H	-2.594833	-3.63976	1.058722
H	-3.463547	-6.19697	-4.13945
H	-1.830107	-6.27587	-4.83502
H	-2.937491	-4.98811	-5.33965
H	-1.664636	-5.11566	0.939682
H	2.224887	-5.09367	0.092041
H	0.601547	-6.00631	5.044336
H	2.26479	-5.43504	4.822785
H	1.125686	-4.50182	5.82837
H	-1.467404	-1.13204	4.037514
H	-2.381487	-1.39488	2.546363
H	-0.942885	-0.36171	2.532517

Cartesian coordinates of 2 (B3LYP-D3(BJ)/def2-SVP, MCH, PCM model) in S_0 state

E = -2987.622426 Hartrees

Symbol	X	Y	Z
C	0.741421	-1.07786	-0.17454
N	-1.665175	-0.98124	0.071049
S	-2.337054	1.513946	0.060019
O	-4.297841	3.464716	0.114076
B	-0.49949	-2.1299	-0.02423
C	-5.423169	2.986297	0.124314
S	1.504903	1.460967	-0.04848
O	-7.958759	0.447156	0.140155
C	-6.70235	3.757805	0.144727
C	-6.896071	5.136877	0.155383
H	-6.043453	5.818662	0.14889
C	-8.21304	5.612819	0.174428
H	-8.399855	6.688879	0.183134
C	-9.302616	4.725337	0.18249
H	-10.318471	5.126207	0.197324
C	-9.103025	3.339131	0.171719
H	-9.94343	2.642311	0.17772
C	-7.792077	2.870205	0.152809
C	-7.286844	1.464363	0.137797
C	-5.80107	1.5583	0.119089
C	-3.009869	-0.99044	0.063761
H	-3.554755	-1.93257	0.053555
C	-3.606176	0.264167	0.076591
C	-5.016619	0.44188	0.09891
H	-5.575552	-0.50235	0.099341
C	-1.142152	0.271335	0.031271
C	0.264267	0.239366	-0.05885
C	2.154933	-1.07822	-0.21547
H	2.77847	-1.97003	-0.28349
C	2.717696	0.195579	-0.17329

C	4.118686	0.522376	-0.21951
H	4.823509	-0.31644	-0.28678
C	-1.023341	-3.0746	-1.2526
C	-0.687801	-2.82269	-2.61092
C	-1.188688	-3.63975	-3.63401
H	-0.904509	-3.4227	-4.66854
C	-2.050768	-4.71137	-3.37863
C	-2.418567	-4.92884	-2.05044
H	-3.113641	-5.74198	-1.819
C	-1.928846	-4.13815	-0.99616
C	0.20186	-1.67478	-3.03355
C	-2.557883	-5.58719	-4.49661
C	-2.411261	-4.51509	0.390904
C	-0.123556	-2.89739	1.378447
C	0.776521	-3.99621	1.281139
C	1.196911	-4.67997	2.429864
H	1.885717	-5.52352	2.317189
C	0.768194	-4.31972	3.711059
C	-0.081706	-3.21674	3.807853
H	-0.413073	-2.88938	4.798131
C	-0.518235	-2.49847	2.681333
C	-1.404328	-1.3029	2.956977
C	1.307341	-4.51443	-0.03993
C	1.206985	-5.09314	4.929005
N	4.489517	1.762239	-0.17871
N	5.775472	2.112822	-0.21194
C	6.814881	1.124052	-0.2891
C	8.796495	-0.82828	-0.43862
C	7.341819	0.5826	0.88771
C	7.267044	0.692692	-1.54013
C	8.262182	-0.2852	-1.61185
C	8.336502	-0.39522	0.809492
C	6.086298	3.489016	-0.16861
C	6.723306	6.23084	-0.08256
C	7.428926	3.908383	-0.19312
C	5.063525	4.454055	-0.1006
C	5.388684	5.809077	-0.05833
C	7.735717	5.269905	-0.15032
H	8.231731	3.173809	-0.2454
H	8.783951	5.577799	-0.17018
H	6.969436	7.294172	-0.04927
H	4.583479	6.54619	-0.00573
H	4.025827	4.126878	-0.08201
H	6.971758	0.933453	1.852837
H	8.754123	-0.81865	1.725436
H	9.57499	-1.59215	-0.49725

H	8.621804	-0.62279	-2.58622
H	6.839535	1.128387	-2.44507
H	0.261691	-1.61598	-4.13009
H	1.226695	-1.7817	-2.64842
H	-0.167838	-0.70663	-2.66574
H	1.509367	-3.72011	-0.76773
H	0.583326	-5.18951	-0.52333
H	-3.340089	-5.10093	0.324759
H	-2.600862	-3.65121	1.039501
H	-3.385755	-6.23	-4.16234
H	-1.759359	-6.24583	-4.87985
H	-2.911808	-4.98682	-5.35016
H	-1.666795	-5.12821	0.922617
H	2.237741	-5.08037	0.116743
H	0.671993	-6.05589	5.004566
H	2.282622	-5.32876	4.891229
H	1.010383	-4.5332	5.855507
H	-1.513374	-1.15083	4.040096
H	-2.417442	-1.42475	2.544836
H	-0.992947	-0.37332	2.537029

Cartesian coordinates of 2 (B3LYP-D3(BJ)/def2-SVP, toluene, PCM model) in S_0 state

E = -2987.623726 Hartrees

Symbol	X	Y	Z
C	0.740218	-1.07706	-0.18511
N	-1.664538	-0.98093	0.068945
S	-2.33729	1.515322	0.058879
O	-4.29158	3.461775	0.119503
B	-0.498383	-2.1301	-0.03026
C	-5.416708	2.986191	0.131118
S	1.502713	1.462989	-0.05561
O	-7.959748	0.453382	0.149629
C	-6.695323	3.76052	0.154996
C	-6.884677	5.140168	0.167856
H	-6.027553	5.816366	0.160458
C	-8.199761	5.619792	0.190189
H	-8.383648	6.696588	0.200697
C	-9.29188	4.735383	0.199272
H	-10.306716	5.139406	0.216665
C	-9.096559	3.34898	0.18626
H	-9.936695	2.651702	0.192943
C	-7.78704	2.876381	0.164061
C	-7.286544	1.467188	0.146388
C	-5.799293	1.558993	0.124588
C	-3.008944	-0.98991	0.062774
H	-3.551489	-1.93347	0.049321

C	-3.606067	0.264209	0.077363
C	-5.016927	0.442109	0.101304
H	-5.579109	-0.5007	0.10046
C	-1.142481	0.271562	0.026957
C	0.263373	0.240322	-0.06803
C	2.153489	-1.07555	-0.22514
H	2.775758	-1.96867	-0.28821
C	2.715014	0.198276	-0.18141
C	4.116365	0.524566	-0.22453
H	4.819912	-0.31622	-0.28702
C	-1.03259	-3.07517	-1.25235
C	-0.707148	-2.8261	-2.61279
C	-1.220211	-3.64139	-3.63044
H	-0.944114	-3.4263	-4.66755
C	-2.084028	-4.70854	-3.36653
C	-2.44126	-4.9237	-2.03551
H	-3.137166	-5.7343	-1.79765
C	-1.939885	-4.13474	-0.98638
C	0.184208	-1.68231	-3.04237
C	-2.602037	-5.58558	-4.47826
C	-2.411335	-4.50806	0.405574
C	-0.114313	-2.89536	1.37019
C	0.779426	-3.99775	1.266851
C	1.201768	-4.68368	2.413192
H	1.884863	-5.53134	2.296634
C	0.782303	-4.3213	3.69589
C	-0.058638	-3.21241	3.798172
H	-0.38055	-2.88184	4.790509
C	-0.497495	-2.49268	2.674949
C	-1.370764	-1.28874	2.953873
C	1.300927	-4.51709	-0.05746
C	1.216701	-5.10003	4.911743
N	4.490722	1.762709	-0.18524
N	5.7779	2.11084	-0.21396
C	6.81408	1.119644	-0.28154
C	8.78817	-0.84293	-0.41334
C	7.323968	0.570344	0.899156
C	7.280993	0.690151	-1.52779
C	8.272044	-0.29223	-1.59081
C	8.314188	-0.41238	0.83008
C	6.092383	3.487199	-0.17752
C	6.733886	6.226375	-0.10533
C	7.435806	3.902665	-0.18764
C	5.070733	4.453686	-0.13075
C	5.398518	5.807663	-0.09499
C	7.74484	5.263637	-0.15188

H	8.237395	3.165612	-0.22284
H	8.793794	5.569726	-0.1602
H	6.981703	7.289466	-0.07745
H	4.594377	6.546891	-0.05884
H	4.032858	4.126787	-0.123
H	6.942693	0.920173	1.860341
H	8.717678	-0.8418	1.749658
H	9.563316	-1.61079	-0.46485
H	8.642841	-0.62776	-2.56181
H	6.866999	1.132794	-2.43572
H	0.240291	-1.62679	-4.13931
H	1.210292	-1.7901	-2.66068
H	-0.18085	-0.7114	-2.677
H	1.507601	-3.72258	-0.7841
H	0.570538	-5.18348	-0.54291
H	-3.340393	-5.09488	0.348286
H	-2.595633	-3.6428	1.05435
H	-3.456104	-6.1953	-4.14747
H	-1.820082	-6.27767	-4.8364
H	-2.922994	-4.98855	-5.34694
H	-1.663328	-5.11788	0.935772
H	2.227072	-5.0915	0.094934
H	0.606871	-6.01157	5.040531
H	2.265985	-5.42505	4.829581
H	1.113215	-4.50449	5.831426
H	-1.474257	-1.13519	4.037357
H	-2.386881	-1.39946	2.54555
H	-0.95027	-0.36364	2.532767

Cartesian coordinates of 2 (B3LYP-D3(BJ)/def2-SVP, $CHCl_3$, PCM model) in S_0 state

E = -2987.628178 Hartrees

Symbol	X	Y	Z
C	0.740706	-1.07728	-0.18163
N	-1.664875	-0.98127	0.068899
S	-2.337453	1.514518	0.06015
O	-4.295143	3.462785	0.118138
B	-0.498827	-2.13003	-0.02885
C	-5.420286	2.98567	0.129639
S	1.503316	1.46237	-0.0538
O	-7.959332	0.449399	0.148654
C	-6.699291	3.758452	0.152333
C	-6.890834	5.137803	0.163952
H	-6.03586	5.816653	0.156381
C	-8.206843	5.615559	0.185297
H	-8.39221	6.692001	0.194802
C	-9.297644	4.72957	0.194649

H	-10.313015	5.131972	0.211254
C	-9.100189	3.343266	0.182885
H	-9.940432	2.646184	0.18981
C	-7.789947	2.872522	0.161655
C	-7.286955	1.464984	0.145214
C	-5.800448	1.558039	0.124042
C	-3.009345	-0.99045	0.062946
H	-3.552948	-1.93338	0.050718
C	-3.606275	0.263845	0.077685
C	-5.016889	0.441477	0.101594
H	-5.577352	-0.50212	0.101343
C	-1.142436	0.271375	0.028219
C	0.263523	0.240025	-0.06516
C	2.154014	-1.07643	-0.22194
H	2.776998	-1.96891	-0.28682
C	2.71596	0.197485	-0.17888
C	4.117091	0.524053	-0.22271
H	4.821313	-0.31585	-0.28564
C	-1.028182	-3.07484	-1.254
C	-0.69934	-2.82317	-2.61346
C	-1.206796	-3.63901	-3.63391
H	-0.928084	-3.42182	-4.66989
C	-2.068364	-4.70935	-3.37414
C	-2.42936	-4.92685	-2.04428
H	-3.123994	-5.73944	-1.80948
C	-1.933524	-4.13734	-0.99259
C	0.189547	-1.67612	-3.03974
C	-2.58024	-5.58669	-4.48859
C	-2.409132	-4.51328	0.39716
C	-0.118146	-2.89609	1.37262
C	0.778992	-3.9965	1.272009
C	1.200886	-4.68112	2.419376
H	1.886874	-5.52668	2.304527
C	0.777356	-4.31968	3.701471
C	-0.068061	-3.21386	3.801251
H	-0.394175	-2.88472	4.792681
C	-0.506535	-2.49522	2.676485
C	-1.386048	-1.29539	2.954109
C	1.304654	-4.51517	-0.05091
C	1.214904	-5.09557	4.91815
N	4.489827	1.763045	-0.18371
N	5.776454	2.112082	-0.2132
C	6.814151	1.121695	-0.28125
C	8.791579	-0.83678	-0.41353
C	7.327445	0.576189	0.89972
C	7.27861	0.69092	-1.52797

C	8.271535	-0.28969	-1.59113
C	8.31964	-0.40465	0.830267
C	6.089379	3.488417	-0.17625
C	6.729078	6.228863	-0.10302
C	7.432614	3.905302	-0.18879
C	5.067171	4.454611	-0.1266
C	5.393877	5.809099	-0.0904
C	7.740744	5.266574	-0.1525
H	8.234763	3.169037	-0.22641
H	8.789493	5.573255	-0.16275
H	6.976212	7.292109	-0.07473
H	4.589205	6.547655	-0.05203
H	4.02926	4.127817	-0.11712
H	6.947935	0.926822	1.861283
H	8.726154	-0.83123	1.749788
H	9.568242	-1.60306	-0.46528
H	8.640649	-0.6265	-2.56228
H	6.861805	1.130295	-2.43616
H	0.246628	-1.6184	-4.13651
H	1.215412	-1.78231	-2.6571
H	-0.17863	-0.70713	-2.67253
H	1.508973	-3.7206	-0.778
H	0.577198	-5.18547	-0.53553
H	-3.336789	-5.10173	0.335866
H	-2.597808	-3.6489	1.045658
H	-3.433638	-6.19914	-4.16122
H	-1.795118	-6.27615	-4.84481
H	-2.899292	-4.98968	-5.35794
H	-1.661451	-5.12274	0.92835
H	2.232839	-5.08564	0.103272
H	0.647155	-6.03744	5.016291
H	2.280517	-5.36925	4.860646
H	1.0581	-4.51961	5.842565
H	-1.492104	-1.14274	4.037462
H	-2.40078	-1.41136	2.544009
H	-0.969847	-0.36806	2.53379

Cartesian coordinates of 2 (B3LYP-D3(BJ)/def2-SVP, THF, PCM model) in S_0 state

E = -2987.6302 Hartrees

Symbol	X	Y	Z
C	0.740948	-1.07743	-0.17938
N	-1.665037	-0.98136	0.069156
S	-2.337462	1.514198	0.060277
O	-4.296502	3.463344	0.116945
B	-0.499067	-2.13	-0.02771
C	-5.421684	2.985634	0.127942

S	1.503733	1.461955	-0.05207
O	-7.959183	0.448046	0.14575
C	-6.700813	3.757826	0.149821
C	-6.893272	5.137056	0.161111
H	-6.039239	5.817076	0.153931
C	-8.209678	5.614039	0.181636
H	-8.395661	6.690325	0.190862
C	-9.299948	4.727406	0.190518
H	-10.315534	5.129149	0.206491
C	-9.101596	3.341147	0.179092
H	-9.941889	2.64415	0.18568
C	-7.791053	2.871175	0.158682
C	-7.287074	1.464333	0.142709
C	-5.800869	1.557839	0.122405
C	-3.009569	-0.9906	0.062819
H	-3.553635	-1.93324	0.051156
C	-3.606343	0.263787	0.077197
C	-5.016877	0.441375	0.100609
H	-5.576675	-0.50251	0.100474
C	-1.14241	0.27131	0.029056
C	0.263682	0.239825	-0.06318
C	2.154316	-1.07696	-0.21974
H	2.777568	-1.96917	-0.28565
C	2.716502	0.196946	-0.17688
C	4.117563	0.523633	-0.22123
H	4.822058	-0.31586	-0.2851
C	-1.02613	-3.07469	-1.25425
C	-0.695019	-2.82241	-2.61324
C	-1.199792	-3.63859	-3.63489
H	-0.919255	-3.42099	-4.67029
C	-2.061032	-4.70988	-3.37701
C	-2.424365	-4.92791	-2.04779
H	-3.118811	-5.74106	-1.81443
C	-1.931094	-4.13803	-0.99495
C	0.193578	-1.6745	-3.03793
C	-2.570644	-5.58675	-4.49294
C	-2.409116	-4.51481	0.393714
C	-0.120181	-2.89656	1.374237
C	0.778346	-3.99617	1.274923
C	1.1998	-4.68033	2.422806
H	1.887041	-5.52499	2.308798
C	0.77424	-4.31939	3.704562
C	-0.073136	-3.21487	3.803163
H	-0.401312	-2.88644	4.794149
C	-0.511091	-2.49653	2.677699
C	-1.393367	-1.29851	2.9546

C	1.306081	-4.51455	-0.04729
C	1.212686	-5.09411	4.921717
N	4.48956	1.762981	-0.18188
N	5.775946	2.11253	-0.2122
C	6.814325	1.122649	-0.28209
C	8.793359	-0.83369	-0.41778
C	7.331014	0.578778	0.898146
C	7.275891	0.691475	-1.52975
C	8.269685	-0.2882	-1.59458
C	8.324158	-0.40104	0.826934
C	6.088098	3.488835	-0.17387
C	6.726833	6.229851	-0.09787
C	7.43118	3.906529	-0.18916
C	5.06563	4.454744	-0.12011
C	5.391776	5.809457	-0.0826
C	7.738828	5.267923	-0.15146
H	8.233607	3.170773	-0.23009
H	8.787438	5.574971	-0.16392
H	6.973605	7.293152	-0.0685
H	4.586863	6.547584	-0.04105
H	4.027745	4.127899	-0.10857
H	6.953703	0.929629	1.860482
H	8.733492	-0.82637	1.745764
H	9.570732	-1.59914	-0.47091
H	8.636604	-0.62544	-2.56638
H	6.856389	1.129395	-2.43739
H	0.251564	-1.61605	-4.1346
H	1.219139	-1.78054	-2.65446
H	-0.175594	-0.70609	-2.67021
H	1.509363	-3.72001	-0.77462
H	0.580061	-5.18676	-0.53149
H	-3.336715	-5.10306	0.330457
H	-2.598965	-3.65076	1.042203
H	-3.416288	-6.20889	-4.16385
H	-1.78087	-6.26657	-4.85717
H	-2.900097	-4.98853	-5.35761
H	-1.662215	-5.12499	0.925247
H	2.235217	-5.08313	0.107834
H	0.660213	-6.04599	5.009335
H	2.283253	-5.34981	4.873224
H	1.037316	-4.5255	5.847281
H	-1.500704	-1.14623	4.037872
H	-2.407419	-1.41683	2.543588
H	-0.979068	-0.37021	2.534611

Cartesian coordinates of 2 (B3LYP-D3(BJ)/def2-SVP, CH_2Cl_2, PCM model) in S_0 state

E = -2987.630842 Hartrees

Symbol	X	Y	Z
C	0.741013	-1.0775	-0.17861
N	-1.665073	-0.98136	0.069546
S	-2.337432	1.514118	0.060464
O	-4.296855	3.463559	0.116814
B	-0.499149	-2.12998	-0.02711
C	-5.422056	2.985681	0.127788
S	1.503896	1.461803	-0.05163
O	-7.959112	0.447724	0.145549
C	-6.701209	3.757709	0.149509
C	-6.89394	5.136904	0.160669
H	-6.040198	5.817285	0.153509
C	-8.210463	5.613659	0.181048
H	-8.396627	6.689898	0.19017
C	-9.300577	4.726838	0.189916
H	-10.316223	5.128388	0.205775
C	-9.101959	3.340591	0.178623
H	-9.942268	2.643623	0.185208
C	-7.791329	2.870847	0.158362
C	-7.287067	1.464215	0.142539
C	-5.800955	1.557838	0.122368
C	-3.009631	-0.99062	0.063191
H	-3.553848	-1.93316	0.051831
C	-3.606339	0.263806	0.077402
C	-5.016848	0.441396	0.100774
H	-5.576454	-0.50257	0.100793
C	-1.142386	0.271303	0.029497
C	0.26375	0.239753	-0.06248
C	2.1544	-1.07716	-0.21916
H	2.77772	-1.96929	-0.28548
C	2.71668	0.196738	-0.17648
C	4.117718	0.523455	-0.22123
H	4.822282	-0.31591	-0.28561
C	-1.025637	-3.07468	-1.25399
C	-0.693876	-2.82234	-2.61287
C	-1.197927	-3.63868	-3.6348
H	-0.916857	-3.42106	-4.67006
C	-2.059141	-4.71017	-3.37737
C	-2.423148	-4.92823	-2.04832
H	-3.117587	-5.74148	-1.8153
C	-1.930547	-4.1382	-0.99521
C	0.194701	-1.67427	-3.03721
C	-2.56821	-5.58692	-4.49366
C	-2.409244	-4.51512	0.393177

C	-0.120703	-2.89668	1.374963
C	0.778197	-3.99607	1.275994
C	1.19959	-4.6801	2.424019
H	1.887183	-5.5245	2.31023
C	0.773514	-4.31928	3.705696
C	-0.074419	-3.21512	3.803988
H	-0.403141	-2.88688	4.794857
C	-0.512264	-2.49687	2.678334
C	-1.395329	-1.29937	2.955055
C	1.30645	-4.51442	-0.04603
C	1.212236	-5.09367	4.922985
N	4.48949	1.76291	-0.18176
N	5.775796	2.112634	-0.21261
C	6.814375	1.12293	-0.28351
C	8.793899	-0.8327	-0.42109
C	7.332634	0.579586	0.896283
C	7.274527	0.691633	-1.53164
C	8.268582	-0.28773	-1.59741
C	8.326069	-0.39988	0.82411
C	6.087704	3.488925	-0.1737
C	6.726133	6.230115	-0.09652
C	7.430723	3.906898	-0.19036
C	5.065167	4.454719	-0.118
C	5.391135	5.809499	-0.07993
C	7.738219	5.268326	-0.15206
H	8.23323	3.171327	-0.23285
H	8.786774	5.575505	-0.16561
H	6.972791	7.293429	-0.06669
H	4.586158	6.547471	-0.03687
H	4.0273	4.127843	-0.10542
H	6.956408	0.930503	1.859017
H	8.736682	-0.8248	1.742552
H	9.571488	-1.59787	-0.47496
H	8.634411	-0.62511	-2.56957
H	6.853791	1.129081	-2.43893
H	0.252898	-1.61568	-4.13386
H	1.220185	-1.78034	-2.65355
H	-0.174673	-0.70599	-2.66938
H	1.509356	-3.71991	-0.77348
H	0.580824	-5.18724	-0.53
H	-3.336937	-5.10314	0.329417
H	-2.599231	-3.65113	1.041675
H	-3.411178	-6.21227	-4.16384
H	-1.776981	-6.2635	-4.8607
H	-2.901309	-4.98835	-5.35671
H	-1.662633	-5.12566	0.924727

H	2.235889	-5.08241	0.10932
H	0.66365	-6.048	5.008028
H	2.283978	-5.34487	4.876723
H	1.032258	-4.52692	5.848791
H	-1.502937	-1.14714	4.038305
H	-2.409218	-1.41841	2.543883
H	-0.981656	-0.3708	2.535066

Cartesian coordinates of 2 (B3LYP-D3(BJ)/def2-SVP, acetone, PCM model) in S_0 state

E = -2987.628178 Hartrees

Symbol	X	Y	Z
C	0.740706	-1.07728	-0.18163
N	-1.664875	-0.98127	0.068899
S	-2.337453	1.514518	0.06015
O	-4.295143	3.462785	0.118138
B	-0.498827	-2.13003	-0.02885
C	-5.420286	2.98567	0.129639
S	1.503316	1.46237	-0.0538
O	-7.959332	0.449399	0.148654
C	-6.699291	3.758452	0.152333
C	-6.890834	5.137803	0.163952
H	-6.03586	5.816653	0.156381
C	-8.206843	5.615559	0.185297
H	-8.39221	6.692001	0.194802
C	-9.297644	4.72957	0.194649
H	-10.313015	5.131972	0.211254
C	-9.100189	3.343266	0.182885
H	-9.940432	2.646184	0.18981
C	-7.789947	2.872522	0.161655
C	-7.286955	1.464984	0.145214
C	-5.800448	1.558039	0.124042
C	-3.009345	-0.99045	0.062946
H	-3.552948	-1.93338	0.050718
C	-3.606275	0.263845	0.077685
C	-5.016889	0.441477	0.101594
H	-5.577352	-0.50212	0.101343
C	-1.142436	0.271375	0.028219
C	0.263523	0.240025	-0.06516
C	2.154014	-1.07643	-0.22194
H	2.776998	-1.96891	-0.28682
C	2.71596	0.197485	-0.17888
C	4.117091	0.524053	-0.22271
H	4.821313	-0.31585	-0.28564
C	-1.028182	-3.07484	-1.254
C	-0.69934	-2.82317	-2.61346
C	-1.206796	-3.63901	-3.63391

H	-0.928084	-3.42182	-4.66989
C	-2.068364	-4.70935	-3.37414
C	-2.42936	-4.92685	-2.04428
H	-3.123994	-5.73944	-1.80948
C	-1.933524	-4.13734	-0.99259
C	0.189547	-1.67612	-3.03974
C	-2.58024	-5.58669	-4.48859
C	-2.409132	-4.51328	0.39716
C	-0.118146	-2.89609	1.37262
C	0.778992	-3.9965	1.272009
C	1.200886	-4.68112	2.419376
H	1.886874	-5.52668	2.304527
C	0.777356	-4.31968	3.701471
C	-0.068061	-3.21386	3.801251
H	-0.394175	-2.88472	4.792681
C	-0.506535	-2.49522	2.676485
C	-1.386048	-1.29539	2.954109
C	1.304654	-4.51517	-0.05091
C	1.214904	-5.09557	4.91815
N	4.489827	1.763045	-0.18371
N	5.776454	2.112082	-0.2132
C	6.814151	1.121695	-0.28125
C	8.791579	-0.83678	-0.41353
C	7.327445	0.576189	0.89972
C	7.27861	0.69092	-1.52797
C	8.271535	-0.28969	-1.59113
C	8.31964	-0.40465	0.830267
C	6.089379	3.488417	-0.17625
C	6.729078	6.228863	-0.10302
C	7.432614	3.905302	-0.18879
C	5.067171	4.454611	-0.1266
C	5.393877	5.809099	-0.0904
C	7.740744	5.266574	-0.1525
H	8.234763	3.169037	-0.22641
H	8.789493	5.573255	-0.16275
H	6.976212	7.292109	-0.07473
H	4.589205	6.547655	-0.05203
H	4.02926	4.127817	-0.11712
H	6.947935	0.926822	1.861283
H	8.726154	-0.83123	1.749788
H	9.568242	-1.60306	-0.46528
H	8.640649	-0.6265	-2.56228
H	6.861805	1.130295	-2.43616
H	0.246628	-1.6184	-4.13651
H	1.215412	-1.78231	-2.6571
H	-0.17863	-0.70713	-2.67253

H	1.508973	-3.7206	-0.778
H	0.577198	-5.18547	-0.53553
H	-3.336789	-5.10173	0.335866
H	-2.597808	-3.6489	1.045658
H	-3.433638	-6.19914	-4.16122
H	-1.795118	-6.27615	-4.84481
H	-2.899292	-4.98968	-5.35794
H	-1.661451	-5.12274	0.92835
H	2.232839	-5.08564	0.103272
H	0.647155	-6.03744	5.016291
H	2.280517	-5.36925	4.860646
H	1.0581	-4.51961	5.842565
H	-1.492104	-1.14274	4.037462
H	-2.40078	-1.41136	2.544009
H	-0.969847	-0.36806	2.53379

Cartesian coordinates of 2 (B3LYP-D3(BJ)/def2-SVP, EtOH, PCM model) in S_0 state
E = -2987.633056 Hartrees

Symbol	X	Y	Z
C	0.741348	-1.07779	-0.17526
N	-1.665168	-0.98128	0.070738
S	-2.337145	1.513952	0.060175
O	-4.297748	3.464499	0.114675
B	-0.499436	-2.12992	-0.02479
C	-5.42305	2.986146	0.125023
S	1.504708	1.46112	-0.04902
O	-7.958829	0.447152	0.141146
C	-6.702236	3.757717	0.145668
C	-6.895818	5.136805	0.156429
H	-6.043033	5.818381	0.149833
C	-8.212723	5.612859	0.175706
H	-8.399448	6.688945	0.184496
C	-9.302372	4.725467	0.183892
H	-10.318199	5.12643	0.198904
C	-9.102918	3.339256	0.173013
H	-9.943305	2.64241	0.179103
C	-7.792014	2.870218	0.153868
C	-7.286909	1.464261	0.138694
C	-5.801087	1.558169	0.119736
C	-3.009837	-0.9905	0.063633
H	-3.554612	-1.9327	0.053208
C	-3.606223	0.264074	0.076762
C	-5.016673	0.441754	0.09928
H	-5.575698	-0.50244	0.099616
C	-1.142206	0.27132	0.030964
C	0.264161	0.239437	-0.05948

C	2.154838	-1.07802	-0.21611
H	2.778349	-1.96988	-0.28385
C	2.717512	0.195796	-0.17383
C	4.118506	0.522588	-0.21977
H	4.823296	-0.31631	-0.28647
C	-1.023673	-3.0746	-1.25295
C	-0.688784	-2.82252	-2.61136
C	-1.190131	-3.63944	-3.6343
H	-0.90649	-3.42221	-4.66894
C	-2.051976	-4.71113	-3.37863
C	-2.419125	-4.9288	-2.05031
H	-3.114027	-5.74203	-1.81865
C	-1.928996	-4.13823	-0.99618
C	0.200596	-1.67448	-3.03422
C	-2.559335	-5.58709	-4.49638
C	-2.410786	-4.51526	0.391075
C	-0.12309	-2.89728	1.377792
C	0.776849	-3.99615	1.280216
C	1.197434	-4.67994	2.428826
H	1.886085	-5.52359	2.315967
C	0.769134	-4.31961	3.710101
C	-0.080498	-3.21648	3.807138
H	-0.411444	-2.88899	4.797513
C	-0.517285	-2.49824	2.680758
C	-1.402934	-1.3024	2.956591
C	1.30728	-4.51437	-0.04101
C	1.207959	-5.09317	4.92794
N	4.489482	1.762392	-0.17926
N	5.775495	2.112826	-0.21203
C	6.814781	1.123889	-0.28789
C	8.796076	-0.829	-0.43494
C	7.340014	0.582153	0.889546
C	7.268543	0.692508	-1.53834
C	8.263518	-0.28563	-1.60883
C	8.334503	-0.39595	0.812577
C	6.086492	3.489043	-0.16964
C	6.723724	6.230784	-0.08547
C	7.429199	3.908165	-0.19255
C	5.063739	4.454235	-0.10418
C	5.389025	5.809226	-0.0628
C	7.736101	5.269681	-0.1507
H	8.23197	3.173406	-0.24281
H	8.784399	5.577443	-0.16928
H	6.969937	7.29412	-0.05292
H	4.583843	6.546502	-0.01218
H	4.026001	4.127124	-0.08683

H	6.968716	0.93304	1.854189
H	8.750756	-0.81961	1.729042
H	9.574433	-1.59309	-0.49259
H	8.624387	-0.62321	-2.58274
H	6.842359	1.12847	-2.44378
H	0.260133	-1.61564	-4.13077
H	1.225554	-1.7812	-2.64935
H	-0.169122	-0.70636	-2.66634
H	1.509388	-3.72	-0.76875
H	0.583042	-5.18912	-0.5245
H	-3.339348	-5.10158	0.325285
H	-2.600588	-3.65139	1.039659
H	-3.389644	-6.22715	-4.16285
H	-1.761912	-6.2486	-4.87701
H	-2.909906	-4.98697	-5.35148
H	-1.6659	-5.12786	0.922773
H	2.237562	-5.08058	0.115444
H	0.670795	-6.0546	5.00504
H	2.282996	-5.33126	4.888777
H	1.014021	-4.53212	5.854341
H	-1.511767	-1.15033	4.039733
H	-2.416163	-1.42381	2.544588
H	-0.9912	-0.37296	2.536669

Cartesian coordinates of 2 (B3LYP-D3(BJ)/def2-SVP, MeCN, PCM model) in S_0 state
E = -2987.633465 Hartrees

Symbol	X	Y	Z
C	0.741421	-1.07786	-0.17454
N	-1.665175	-0.98124	0.071049
S	-2.337054	1.513946	0.060019
O	-4.297841	3.464716	0.114076
B	-0.49949	-2.1299	-0.02423
C	-5.423169	2.986297	0.124314
S	1.504903	1.460967	-0.04848
O	-7.958759	0.447156	0.140155
C	-6.70235	3.757805	0.144727
C	-6.896071	5.136877	0.155383
H	-6.043453	5.818662	0.14889
C	-8.21304	5.612819	0.174428
H	-8.399855	6.688879	0.183134
C	-9.302616	4.725337	0.18249
H	-10.318471	5.126207	0.197324
C	-9.103025	3.339131	0.171719
H	-9.94343	2.642311	0.17772
C	-7.792077	2.870205	0.152809
C	-7.286844	1.464363	0.137797

C	-5.80107	1.5583	0.119089
C	-3.009869	-0.99044	0.063761
H	-3.554755	-1.93257	0.053555
C	-3.606176	0.264167	0.076591
C	-5.016619	0.44188	0.09891
H	-5.575552	-0.50235	0.099341
C	-1.142152	0.271335	0.031271
C	0.264267	0.239366	-0.05885
C	2.154933	-1.07822	-0.21547
H	2.77847	-1.97003	-0.28349
C	2.717696	0.195579	-0.17329
C	4.118686	0.522376	-0.21951
H	4.823509	-0.31644	-0.28678
C	-1.023341	-3.0746	-1.2526
C	-0.687801	-2.82269	-2.61092
C	-1.188688	-3.63975	-3.63401
H	-0.904509	-3.4227	-4.66854
C	-2.050768	-4.71137	-3.37863
C	-2.418567	-4.92884	-2.05044
H	-3.113641	-5.74198	-1.819
C	-1.928846	-4.13815	-0.99616
C	0.20186	-1.67478	-3.03355
C	-2.557883	-5.58719	-4.49661
C	-2.411261	-4.51509	0.390904
C	-0.123556	-2.89739	1.378447
C	0.776521	-3.99621	1.281139
C	1.196911	-4.67997	2.429864
H	1.885717	-5.52352	2.317189
C	0.768194	-4.31972	3.711059
C	-0.081706	-3.21674	3.807853
H	-0.413073	-2.88938	4.798131
C	-0.518235	-2.49847	2.681333
C	-1.404328	-1.3029	2.956977
C	1.307341	-4.51443	-0.03993
C	1.206985	-5.09314	4.929005
N	4.489517	1.762239	-0.17871
N	5.775472	2.112822	-0.21194
C	6.814881	1.124052	-0.2891
C	8.796495	-0.82828	-0.43862
C	7.341819	0.5826	0.88771
C	7.267044	0.692692	-1.54013
C	8.262182	-0.2852	-1.61185
C	8.336502	-0.39522	0.809492
C	6.086298	3.489016	-0.16861
C	6.723306	6.23084	-0.08256
C	7.428926	3.908383	-0.19312

C	5.063525	4.454055	-0.1006
C	5.388684	5.809077	-0.05833
C	7.735717	5.269905	-0.15032
H	8.231731	3.173809	-0.2454
H	8.783951	5.577799	-0.17018
H	6.969436	7.294172	-0.04927
H	4.583479	6.54619	-0.00573
H	4.025827	4.126878	-0.08201
H	6.971758	0.933453	1.852837
H	8.754123	-0.81865	1.725436
H	9.57499	-1.59215	-0.49725
H	8.621804	-0.62279	-2.58622
H	6.839535	1.128387	-2.44507
H	0.261691	-1.61598	-4.13009
H	1.226695	-1.7817	-2.64842
H	-0.167838	-0.70663	-2.66574
H	1.509367	-3.72011	-0.76773
H	0.583326	-5.18951	-0.52333
H	-3.340089	-5.10093	0.324759
H	-2.600862	-3.65121	1.039501
H	-3.385755	-6.23	-4.16234
H	-1.759359	-6.24583	-4.87985
H	-2.911808	-4.98682	-5.35016
H	-1.666795	-5.12821	0.922617
H	2.237741	-5.08037	0.116743
H	0.671993	-6.05589	5.004566
H	2.282622	-5.32876	4.891229
H	1.010383	-4.5332	5.855507
H	-1.513374	-1.15083	4.040096
H	-2.417442	-1.42475	2.544836
H	-0.992947	-0.37332	2.537029

Cartesian coordinates of 3 (B3LYP-D3(BJ)/def2-SVP, *n*-hexane, PCM model) in S_0 state

E = -2604.367453 Hartrees

Symbol	X	Y	Z
C	2.177937	0.329197	-0.28587
N	-0.04467	-0.56881	0.051604
S	-1.69273	1.424749	-0.00661
O	-4.27837	2.387365	0.075838
B	1.490311	-1.13438	-0.05201
C	-5.10277	1.488105	0.138042
S	1.819404	2.961261	-0.26644
O	-6.36472	-1.86741	0.307712
C	-6.58752	1.663905	0.186577
C	-7.3326	2.840276	0.171538
H	-6.83376	3.810012	0.117783

C	-8.7274	2.732139	0.227231
H	-9.3418	3.635463	0.217173
C	-9.35289	1.475727	0.296207
H	-10.4433	1.423006	0.338619
C	-8.59923	0.295737	0.311178
H	-9.07209	-0.68689	0.364602
C	-7.21272	0.408154	0.255448
C	-6.17189	-0.66696	0.255776
C	-4.85899	0.032175	0.180503
C	-1.26308	-1.13235	0.092517
H	-1.36525	-2.21566	0.124766
C	-2.32779	-0.23903	0.092046
C	-3.68282	-0.66029	0.159326
H	-3.8036	-1.75058	0.202059
C	-0.089	0.786682	-0.04632
C	1.197685	1.334924	-0.19202
C	3.460107	0.913543	-0.38127
H	4.394704	0.355272	-0.44069
C	3.44513	2.30758	-0.40015
C	4.57926	3.185113	-0.5141
H	5.564469	2.705411	-0.57515
C	1.374882	-2.27122	-1.22124
C	1.537935	-1.97115	-2.60032
C	1.394766	-2.97167	-3.5707
H	1.534251	-2.70813	-4.62393
C	1.063884	-4.289	-3.23985
C	0.855329	-4.57272	-1.89034
H	0.567813	-5.58817	-1.60028
C	0.999936	-3.59953	-0.88716
C	1.85592	-0.57952	-3.09966
H	2.840813	-0.23033	-2.75612
H	1.125583	0.164081	-2.7488
H	1.856952	-0.55403	-4.19921
C	0.939163	-5.35165	-4.30225
H	1.931194	-5.65969	-4.67599
H	0.368561	-4.98773	-5.17186
H	0.437431	-6.25176	-3.9166
C	0.753186	-4.07112	0.53263
H	0.159406	-4.99756	0.528293
H	0.229419	-3.33515	1.155325
H	1.696875	-4.28117	1.059396
C	2.18347	-1.60924	1.358248
C	3.453219	-2.24418	1.259316
C	4.142519	-2.64449	2.411147
H	5.114227	-3.13625	2.297905
C	3.632543	-2.4362	3.695413

C	2.408251	-1.77424	3.791458
H	1.994769	-1.56724	4.783345
C	1.690056	-1.34912	2.661818
C	0.398079	-0.60934	2.932781
H	-0.48551	-1.1531	2.564974
H	0.383416	0.386844	2.466824
H	0.260319	-0.46527	4.013804
C	4.119955	-2.55564	-0.06517
H	5.204157	-2.68392	0.073372
H	3.961747	-1.78002	-0.8236
H	3.72694	-3.48652	-0.50354
C	4.374603	-2.91169	4.918798
H	4.016016	-2.40906	5.829744
H	5.457563	-2.72913	4.83066
H	4.243323	-3.9978	5.068482
N	4.398597	4.473294	-0.53287
N	5.400566	5.323308	-0.59938
C	6.773182	4.890518	-0.7521
H	7.428196	5.770143	-0.75901
H	6.921292	4.329055	-1.69443
H	7.073813	4.236455	0.084155
C	5.057394	6.716865	-0.80408
H	5.199915	7.025879	-1.85674
H	5.677049	7.365416	-0.16462
H	4.001759	6.847807	-0.53818

Cartesian coordinates of 3 (B3LYP-D3(BJ)/def2-SVP, MCH, PCM model) in S_0 state

E = -2604.368087 Hartrees

Symbol	X	Y	Z
C	2.178053	0.329467	-0.28532
N	-0.0447	-0.56873	0.051447
S	-1.69277	1.424846	-0.00653
O	-4.27906	2.387298	0.075762
B	1.490249	-1.13415	-0.0518
C	-5.10332	1.487761	0.137784
S	1.819395	2.961517	-0.26664
O	-6.36439	-1.86803	0.306976
C	-6.58807	1.663279	0.18616
C	-7.33348	2.839431	0.171145
H	-6.83515	3.80943	0.117531
C	-8.72829	2.730901	0.226682
H	-9.34291	3.634056	0.216634
C	-9.35344	1.474321	0.295487
H	-10.4438	1.421274	0.337782
C	-8.59943	0.294516	0.310434
H	-9.07225	-0.68813	0.363733

C	-7.21294	0.407316	0.254857
C	-6.17179	-0.66736	0.255178
C	-4.85914	0.031957	0.180119
C	-1.26303	-1.13227	0.09231
H	-1.36539	-2.21556	0.124662
C	-2.32783	-0.23897	0.091849
C	-3.68272	-0.66032	0.158972
H	-3.80314	-1.75063	0.201563
C	-0.08896	0.786916	-0.04627
C	1.197602	1.335152	-0.19172
C	3.46015	0.913705	-0.38085
H	4.39482	0.355582	-0.44042
C	3.445289	2.307883	-0.40011
C	4.57923	3.185304	-0.51429
H	5.564541	2.70596	-0.57542
C	1.375687	-2.2707	-1.22154
C	1.538976	-1.96999	-2.6005
C	1.397091	-2.97029	-3.57134
H	1.536745	-2.70623	-4.62442
C	1.067249	-4.28809	-3.24114
C	0.858258	-4.57243	-1.8918
H	0.571367	-5.58821	-1.60222
C	1.001565	-3.59944	-0.88819
C	1.855772	-0.57788	-3.09932
H	2.840451	-0.22809	-2.7558
H	1.124907	0.164945	-2.74794
H	1.856554	-0.55194	-4.19885
C	0.94397	-5.35049	-4.30397
H	1.936471	-5.65781	-4.67706
H	0.37387	-4.98661	-5.17393
H	0.442507	-6.25102	-3.91897
C	0.754221	-4.0717	0.531256
H	0.16103	-4.9985	0.5262
H	0.229512	-3.33626	1.153756
H	1.697751	-4.28134	1.058471
C	2.182778	-1.60947	1.3587
C	3.452798	-2.24401	1.260312
C	4.141614	-2.64457	2.412382
H	5.113552	-3.13596	2.299488
C	3.630853	-2.43694	3.696481
C	2.406249	-1.77543	3.792051
H	1.992112	-1.56898	4.783781
C	1.688514	-1.35011	2.66213
C	0.395996	-0.61112	2.932774
H	-0.4871	-1.15544	2.564625
H	0.380788	0.385082	2.466898

H	0.257829	-0.46728	4.013776
C	4.120381	-2.55486	-0.06389
H	5.204589	-2.68249	0.07513
H	3.962022	-1.77922	-0.82226
H	3.728116	-3.48597	-0.50244
C	4.372454	-2.91258	4.920112
H	4.012362	-2.41126	5.831184
H	5.455251	-2.72853	4.833081
H	4.24252	-3.999	5.068652
N	4.39809	4.47369	-0.53339
N	5.399487	5.323608	-0.60086
C	6.772698	4.891383	-0.75085
H	7.427026	5.771454	-0.75915
H	6.921918	4.327963	-1.69172
H	7.072705	4.239368	0.087232
C	5.056833	6.718185	-0.79994
H	5.204713	7.032229	-1.85026
H	5.673163	7.363708	-0.15422
H	3.999883	6.847433	-0.5386

Cartesian coordinates of 3 (B3LYP-D3(BJ)/def2-SVP, toluene, PCM model) in S_0 state
E = -2604.369406 Hartrees

Symbol	X	Y	Z
C	2.178319	0.329955	-0.28419
N	-0.04475	-0.56857	0.05108
S	-1.69282	1.42506	-0.00651
O	-4.28048	2.387241	0.075576
B	1.490113	-1.13375	-0.05134
C	-5.10444	1.487132	0.137239
S	1.819459	2.961977	-0.26717
O	-6.36372	-1.86922	0.305457
C	-6.58916	1.662079	0.185332
C	-7.33525	2.837787	0.170401
H	-6.83796	3.80832	0.11707
C	-8.7301	2.728462	0.225651
H	-9.34517	3.631276	0.215651
C	-9.35455	1.471542	0.294105
H	-10.4449	1.417834	0.336186
C	-8.59982	0.292109	0.308972
H	-9.07257	-0.69057	0.362011
C	-7.21339	0.405685	0.253675
C	-6.17158	-0.6681	0.253952
C	-4.85945	0.031588	0.179306
C	-1.26296	-1.1321	0.091864
H	-1.36571	-2.21534	0.124508
C	-2.32792	-0.23882	0.091376

C	-3.68253	-0.66032	0.158217
H	-3.80224	-1.75066	0.200561
C	-0.08886	0.787385	-0.04629
C	1.197475	1.335574	-0.19118
C	3.46029	0.913931	-0.37993
H	4.3951	0.356091	-0.43977
C	3.445699	2.308403	-0.40001
C	4.579271	3.185567	-0.51459
H	5.5648	2.706949	-0.57568
C	1.377318	-2.26968	-1.22214
C	1.54116	-1.96768	-2.60084
C	1.401808	-2.96755	-3.57264
H	1.541858	-2.70243	-4.6254
C	1.073947	-4.28624	-3.24375
C	0.864017	-4.57184	-1.89476
H	0.578298	-5.58824	-1.60619
C	1.004753	-3.59925	-0.89029
C	1.855704	-0.57466	-3.0986
H	2.839959	-0.22373	-2.75507
H	1.123814	0.166675	-2.74624
H	1.856062	-0.54782	-4.19811
C	0.953544	-5.34814	-4.30747
H	1.947007	-5.65437	-4.67887
H	0.384913	-4.98417	-5.17833
H	0.452165	-6.24932	-3.92391
C	0.756158	-4.07289	0.528456
H	0.16399	-5.00031	0.521933
H	0.229708	-3.33844	1.150601
H	1.699358	-4.28192	1.056517
C	2.181307	-1.60996	1.359662
C	3.451876	-2.2437	1.262438
C	4.13971	-2.64468	2.415029
H	5.112116	-3.13532	2.302887
C	3.627351	-2.4383	3.698772
C	2.402104	-1.77768	3.793317
H	1.986633	-1.57226	4.784711
C	1.6853	-1.35205	2.662789
C	0.391668	-0.61467	2.932727
H	-0.4904	-1.16017	2.5639
H	0.375331	0.381533	2.466959
H	0.252655	-0.47125	4.013675
C	4.121194	-2.55337	-0.06117
H	5.205404	-2.67973	0.078859
H	3.962577	-1.77775	-0.81944
H	3.730419	-3.48499	-0.50002
C	4.368002	-2.91412	4.922946

H	4.005154	-2.41509	5.834166
H	5.450516	-2.72749	4.837969
H	4.24042	-4.00108	5.069513
N	4.39715	4.474374	-0.53461
N	5.397394	5.324096	-0.60401
C	6.771851	4.893001	-0.74794
H	7.424791	5.773964	-0.75902
H	6.923624	4.325436	-1.68563
H	7.070279	4.245329	0.094091
C	5.055833	6.720649	-0.79193
H	5.215276	7.044781	-1.83724
H	5.664896	7.359962	-0.13324
H	3.996116	6.846558	-0.54059

Cartesian coordinates of 3 (B3LYP-D3(BJ)/def2-SVP, $CHCl_3$, PCM model) in S_0 state
E = -2604.373939 Hartrees

Symbol	X	Y	Z
C	2.179324	0.330092	-0.28244
N	-0.04508	-0.56817	0.049625
S	-1.69244	1.426121	-0.00872
O	-4.28474	2.38846	0.073649
B	1.489235	-1.13331	-0.04988
C	-5.10781	1.486555	0.135079
S	1.821035	2.962179	-0.27302
O	-6.36165	-1.87121	0.303237
C	-6.59239	1.659924	0.182551
C	-7.34047	2.834298	0.167148
H	-6.84641	3.806444	0.11384
C	-8.73543	2.722706	0.221911
H	-9.3518	3.624499	0.211508
C	-9.35784	1.464846	0.290362
H	-10.448	1.409227	0.332057
C	-8.60097	0.286482	0.305724
H	-9.07376	-0.69615	0.358808
C	-7.2147	0.402288	0.250926
C	-6.17089	-0.66871	0.251693
C	-4.86039	0.031913	0.177439
C	-1.26302	-1.13122	0.091285
H	-1.36738	-2.21421	0.126564
C	-2.32821	-0.23764	0.089919
C	-3.68197	-0.65914	0.156925
H	-3.79966	-1.74951	0.199897
C	-0.08816	0.788705	-0.04822
C	1.197607	1.336017	-0.19214
C	3.461174	0.912412	-0.37954
H	4.396145	0.35497	-0.44024

C	3.448215	2.307912	-0.40345
C	4.580909	3.18358	-0.51967
H	5.566977	2.706984	-0.58055
C	1.381237	-2.26855	-1.22273
C	1.546789	-1.96411	-2.60099
C	1.413784	-2.96343	-3.57475
H	1.555047	-2.69626	-4.62683
C	1.090661	-4.28423	-3.24852
C	0.878009	-4.5722	-1.9002
H	0.594844	-5.58989	-1.61364
C	1.012272	-3.60004	-0.89408
C	1.855959	-0.56918	-3.09702
H	2.83941	-0.21569	-2.75397
H	1.121831	0.168763	-2.74231
H	1.854916	-0.54081	-4.19647
C	0.977799	-5.34554	-4.31378
H	1.974005	-5.65285	-4.67686
H	0.417605	-4.98025	-5.18946
H	0.47226	-6.2463	-3.93475
C	0.760057	-4.0763	0.52306
H	0.169284	-5.00449	0.513233
H	0.230166	-3.34345	1.143969
H	1.702426	-4.28504	1.052837
C	2.176448	-1.61053	1.363293
C	3.44872	-2.24198	1.270356
C	4.134245	-2.64178	2.425024
H	5.1081	-3.13017	2.315711
C	3.617674	-2.43651	3.707558
C	2.39029	-1.77878	3.798208
H	1.971145	-1.57468	4.788345
C	1.675584	-1.35472	2.665258
C	0.378285	-0.62249	2.932295
H	-0.50041	-1.17255	2.562367
H	0.357809	0.373039	2.465395
H	0.23689	-0.47888	4.012903
C	4.122787	-2.55062	-0.05107
H	5.207012	-2.67292	0.091766
H	3.963153	-1.77646	-0.81045
H	3.736264	-3.48464	-0.48869
C	4.356375	-2.90986	4.933995
H	3.983825	-2.41782	5.845034
H	5.437521	-2.71297	4.855189
H	4.238667	-3.99855	5.075616
N	4.396006	4.473953	-0.54336
N	5.392668	5.322451	-0.62088
C	6.771209	4.894662	-0.74145

H	7.419645	5.778301	-0.76606
H	6.931774	4.308914	-1.6654
H	7.063701	4.265699	0.116847
C	5.056141	6.726154	-0.76319
H	5.268526	7.092323	-1.78395
H	5.629933	7.337812	-0.04856
H	3.98503	6.839756	-0.55969

Cartesian coordinates of 3 (B3LYP-D3(BJ)/def2-SVP, THF, PCM model) in S_0 state

E = -2604.37601 Hartrees

Symbol	X	Y	Z
C	2.179687	0.329128	-0.28316
N	-0.04534	-0.56809	0.049663
S	-1.6919	1.426869	-0.00958
O	-4.28628	2.389906	0.073095
B	1.488466	-1.13376	-0.04917
C	-5.10905	1.487313	0.134705
S	1.822653	2.961361	-0.27741
O	-6.36096	-1.87079	0.304549
C	-6.59355	1.660207	0.181454
C	-7.34232	2.834094	0.164923
H	-6.84951	3.806834	0.111178
C	-8.73737	2.721756	0.219129
H	-9.35418	3.623177	0.207823
C	-9.35908	1.463612	0.288144
H	-10.4492	1.407347	0.329369
C	-8.60146	0.285608	0.304665
H	-9.07447	-0.6969	0.358212
C	-7.21522	0.402157	0.250434
C	-6.17066	-0.66774	0.252339
C	-4.86075	0.03308	0.178074
C	-1.26329	-1.13061	0.092546
H	-1.36859	-2.21344	0.129777
C	-2.32835	-0.23663	0.090631
C	-3.6818	-0.65782	0.158267
H	-3.79881	-1.74815	0.202349
C	-0.08764	0.789142	-0.04911
C	1.198014	1.335572	-0.19353
C	3.461635	0.910152	-0.38223
H	4.396384	0.352486	-0.44429
C	3.449942	2.306133	-0.40809
C	4.582454	3.180656	-0.52622
H	5.568396	2.704523	-0.5895
C	1.381324	-2.26955	-1.22193
C	1.546769	-1.96516	-2.60036
C	1.414849	-2.96484	-3.57415

H	1.555998	-2.69766	-4.62624
C	1.092957	-4.28605	-3.24786
C	0.880046	-4.57394	-1.89942
H	0.597378	-5.59175	-1.61277
C	1.013198	-3.60137	-0.8934
C	1.854439	-0.57003	-3.0969
H	2.838139	-0.2161	-2.75507
H	1.120256	0.167337	-2.74115
H	1.851998	-0.54183	-4.19634
C	0.981754	-5.34784	-4.31285
H	1.978747	-5.65892	-4.67055
H	0.427631	-4.98123	-5.19178
H	0.471308	-6.24657	-3.9356
C	0.760383	-4.07754	0.523625
H	0.169126	-5.00537	0.513624
H	0.230533	-3.34438	1.144111
H	1.702794	-4.28703	1.053122
C	2.174914	-1.61011	1.364959
C	3.447885	-2.24067	1.273588
C	4.133648	-2.63807	2.429075
H	5.108077	-3.12552	2.32076
C	3.616564	-2.43127	3.711318
C	2.388158	-1.775	3.800501
H	1.968316	-1.57017	4.790195
C	1.673094	-1.35353	2.666553
C	0.374153	-0.62372	2.932585
H	-0.50309	-1.17631	2.563088
H	0.351235	0.371162	2.464472
H	0.232327	-0.47931	4.013022
C	4.122755	-2.55079	-0.04708
H	5.207111	-2.67107	0.096094
H	3.961944	-1.77838	-0.80793
H	3.737702	-3.48657	-0.4823
C	4.355988	-2.9012	4.938689
H	3.977763	-2.4134	5.849636
H	5.435856	-2.69642	4.862467
H	4.246166	-3.99096	5.078135
N	4.396913	4.471875	-0.5498
N	5.392151	5.319343	-0.63377
C	6.771957	4.892447	-0.74696
H	7.418574	5.776862	-0.78282
H	6.93296	4.295758	-1.66328
H	7.064579	4.274557	0.119447
C	5.058782	6.726806	-0.74563
H	5.300785	7.119252	-1.74934
H	5.611891	7.319106	0.00124

H	3.982378	6.83483	-0.56928

Cartesian coordinates of 3 (B3LYP-D3(BJ)/def2-SVP, CH_2Cl_2, PCM model) in S_0 state
E = -2604.376668 Hartrees

Symbol	X	Y	Z
C	2.179775	0.328665	0.28359
N	-0.04545	-0.56808	-0.04985
S	-1.69167	1.427144	0.009693
O	-4.2867	2.390489	-0.07306
B	1.488156	-1.134	0.048937
C	-5.1094	1.487701	-0.13474
S	1.823305	2.960963	0.27891
O	-6.36078	-1.87046	-0.30533
C	-6.59388	1.660481	-0.18115
C	-7.34284	2.834233	-0.16409
H	-6.85038	3.807141	-0.11016
C	-8.73792	2.721701	-0.21802
H	-9.35484	3.623018	-0.2063
C	-9.35945	1.46349	-0.28728
H	-10.4495	1.407054	-0.32828
C	-8.60161	0.285581	-0.30434
H	-9.07472	-0.69686	-0.35809
C	-7.21538	0.402324	-0.25039
C	-6.1706	-0.66725	-0.25282
C	-4.86087	0.033596	-0.17856
C	-1.26341	-1.13039	-0.09324
H	-1.36905	-2.21316	-0.13117
C	-2.3284	-0.23624	-0.09116
C	-3.68177	-0.65729	-0.15906
H	-3.7986	-1.7476	-0.2036
C	-0.08745	0.789253	0.049277
C	1.198195	1.335333	0.194002
C	3.461776	0.909195	0.383536
H	4.396404	0.351393	0.446256
C	3.450567	2.305328	0.409989
C	4.583052	3.179422	0.528959
H	5.56888	2.703359	0.593548
C	2.174568	-1.6099	-1.36547
C	3.447748	-2.24019	-1.27449
C	4.133838	-2.63654	-2.43019
H	5.108436	-3.1237	-2.3221
C	3.616862	-2.42894	-3.7124
C	2.388117	-1.77318	-3.80121
H	1.968241	-1.56791	-4.7908
C	1.672684	-1.35282	-2.66699
C	4.122618	-2.55107	0.046001

H	3.737945	-3.48751	0.480185
H	3.9613	-1.77939	0.807459
H	5.207041	-2.67069	-0.09709
C	4.356805	-2.89742	-4.94002
H	4.249737	-3.98751	-5.07897
H	5.436202	-2.68995	-4.8644
H	3.97684	-2.41091	-5.85092
C	0.373221	-0.62382	-2.93281
H	0.23138	-0.47908	-4.0132
H	0.349417	0.370819	-2.46424
H	-0.50357	-1.17729	-2.56359
C	1.381048	-2.27007	1.221519
C	1.546306	-1.96585	2.600057
C	1.414455	-2.96572	3.573742
H	1.555462	-2.69866	4.625885
C	1.092817	-4.28699	3.247252
C	0.879997	-4.57471	1.89872
H	0.597407	-5.59249	1.611903
C	1.013076	-3.60192	0.892847
C	0.760253	-4.07789	-0.52423
H	1.702749	-4.28781	-1.05344
H	0.230706	-3.34447	-1.14464
H	0.168637	-5.00548	-0.51437
C	1.853643	-0.57075	3.096931
H	1.85061	-0.54271	4.196369
H	1.119605	0.166579	2.740818
H	2.837529	-0.21681	2.75567
C	0.98177	-5.34899	4.312073
H	0.470639	-6.24737	3.934927
H	0.428535	-4.98227	5.191503
H	1.978848	-5.66066	4.669016
N	4.397417	4.470927	0.552068
N	5.392231	5.318016	0.638495
C	6.772311	4.891288	0.750377
H	7.418395	5.775859	0.790444
H	7.065626	4.27702	-0.11842
H	6.932656	4.291021	1.664295
C	5.059974	6.726646	0.739492
H	5.606503	7.311903	-0.01783
H	5.311328	7.128047	1.737149
H	3.982081	6.833181	0.571687

Cartesian coordinates of 3 (B3LYP-D3(BJ)/def2-SVP, acetone, PCM model) in S_0 state
E = -2604.376668 Hartrees

Symbol	X	Y	Z
C	2.179959	0.326617	-0.28604
N	-0.04585	-0.56815	0.050707
S	-1.69081	1.428052	-0.01052
O	-4.28774	2.392657	0.072335
B	1.487005	-1.13514	-0.0482
C	-5.11031	1.489399	0.134786
S	1.825699	2.959162	-0.28526
O	-6.36043	-1.86871	0.309751
C	-6.59469	1.662001	0.180054
C	-7.34408	2.835411	0.160673
H	-6.85259	3.808729	0.105551
C	-8.73927	2.722478	0.213833
H	-9.35645	3.623535	0.20027
C	-9.36038	1.464171	0.284618
H	-10.4504	1.407362	0.324939
C	-8.60205	0.286479	0.304051
H	-9.07558	-0.69571	0.359011
C	-7.21582	0.403627	0.250878
C	-6.1705	-0.66508	0.255593
C	-4.86119	0.035685	0.180874
C	-1.26396	-1.12964	0.096258
H	-1.37071	-2.21219	0.137019
C	-2.32856	-0.23484	0.093401
C	-3.68174	-0.65528	0.162617
H	-3.79813	-1.74548	0.209313
C	-0.08679	0.789423	-0.05022
C	1.198895	1.334168	-0.19652
C	3.462205	0.905355	-0.38943
H	4.396304	0.34691	-0.45449
C	3.452715	2.301939	-0.41804
C	4.5852	3.174557	-0.54016
H	5.570523	2.698547	-0.60989
C	1.379268	-2.27278	-1.21946
C	1.543606	-1.97002	-2.59857
C	1.411033	-2.97102	-3.57124
H	1.551345	-2.7051	-4.62375
C	1.089581	-4.29208	-3.24317
C	0.877406	-4.57831	-1.89408
H	0.594592	-5.59566	-1.60599
C	1.01122	-3.6043	-0.88933
C	1.850551	-0.57552	-3.09748
H	2.835326	-0.22191	-2.7585
H	1.117496	0.162366	-2.74052

H	1.845393	-0.54868	-4.19692
C	0.978247	-5.35545	-4.30664
H	1.975479	-5.67259	-4.65833
H	0.431222	-4.98755	-5.18939
H	0.461204	-6.25072	-3.93019
C	0.758785	-4.07864	0.528327
H	0.165161	-5.0049	0.51978
H	0.231285	-3.34359	1.148456
H	1.701684	-4.29059	1.056152
C	2.173933	-1.60864	1.367097
C	3.447668	-2.23831	1.277154
C	4.135554	-2.63006	2.433498
H	5.110596	-3.11643	2.326016
C	3.619809	-2.41848	3.715716
C	2.390024	-1.76427	3.803497
H	1.970679	-1.55663	4.792815
C	1.672649	-1.34869	2.668469
C	0.371658	-0.62211	2.933693
H	-0.50385	-1.17857	2.566052
H	0.344838	0.371491	2.463087
H	0.230196	-0.47566	4.013889
C	4.121704	-2.55292	-0.04286
H	5.206366	-2.67073	0.099493
H	3.958669	-1.78408	-0.80677
H	3.737683	-3.49165	-0.47279
C	4.362212	-2.88104	4.944146
H	3.977924	-2.39701	5.85451
H	5.440141	-2.66587	4.869119
H	4.263264	-3.97184	5.083271
N	4.399501	4.466958	-0.56099
N	5.392942	5.312829	-0.65665
C	6.773733	4.886507	-0.76545
H	7.417996	5.771378	-0.82164
H	6.930636	4.273225	-1.67071
H	7.070694	4.285488	0.111551
C	5.064785	6.72497	-0.7173
H	5.350066	7.158891	-1.69125
H	5.58661	7.283104	0.077507
H	3.982117	6.827386	-0.58101

Cartesian coordinates of 3 (B3LYP-D3(BJ)/def2-SVP, EtOH, PCM model) in S_0 state

E = -2604.378948 Hartrees

Symbol	X	Y	Z
C	2.179982	0.326253	-0.28648
N	-0.04592	-0.56817	0.050874
S	-1.69067	1.428186	-0.0107

O	-4.28787	2.393008	0.072153
B	1.48682	-1.13534	-0.04808
C	-5.11043	1.489695	0.134784
S	1.82609	2.958837	-0.28634
O	-6.36039	-1.86838	0.310622
C	-6.59479	1.662288	0.179887
C	-7.34423	2.835656	0.160094
H	-6.85286	3.809023	0.104729
C	-8.73943	2.722685	0.21316
H	-9.35664	3.62371	0.199274
C	-9.3605	1.464377	0.284257
H	-10.4505	1.40753	0.324493
C	-8.60211	0.286709	0.304111
H	-9.07571	-0.69543	0.359318
C	-7.21588	0.403895	0.251033
C	-6.17049	-0.6647	0.256143
C	-4.86124	0.036039	0.181298
C	-1.26406	-1.12953	0.0968
H	-1.37098	-2.21203	0.138051
C	-2.32859	-0.23462	0.093791
C	-3.68175	-0.65495	0.163256
H	-3.79808	-1.74513	0.210352
C	-0.08669	0.789426	-0.0504
C	1.199016	1.333951	-0.19697
C	3.462275	0.904702	-0.39042
H	4.396282	0.346139	-0.45583
C	3.453057	2.301348	-0.41938
C	4.585549	3.173741	-0.54201
H	5.57079	2.697731	-0.61254
C	1.378913	-2.27326	-1.21909
C	1.543118	-1.97079	-2.5983
C	1.410336	-2.972	-3.57077
H	1.550552	-2.7063	-4.62335
C	1.088804	-4.29299	-3.2424
C	0.876733	-4.57891	-1.89321
H	0.593812	-5.59617	-1.60488
C	1.010759	-3.60469	-0.88867
C	1.85013	-0.57644	-3.09758
H	2.835071	-0.22295	-2.75896
H	1.117289	0.16162	-2.74054
H	1.844659	-0.54983	-4.19703
C	0.977309	-5.35659	-4.30563
H	1.974519	-5.67455	-4.65663
H	0.431112	-4.98854	-5.18882
H	0.459427	-6.25138	-3.9292
C	0.758396	-4.07871	0.529095

H	0.164304	-5.00467	0.520805
H	0.231384	-3.34331	1.149212
H	1.701357	-4.29114	1.056642
C	2.173884	-1.60843	1.367342
C	3.44769	-2.23803	1.277524
C	4.135927	-2.62898	2.433949
H	5.111022	-3.11525	2.326534
C	3.620468	-2.41666	3.716186
C	2.390532	-1.76269	3.803839
H	1.971336	-1.5546	4.793126
C	1.672779	-1.34794	2.668703
C	0.371571	-0.6217	2.933861
H	-0.50376	-1.17863	2.566533
H	0.344268	0.371712	2.462894
H	0.230218	-0.47493	4.014027
C	4.121507	-2.55332	-0.04244
H	5.206204	-2.67094	0.099752
H	3.95823	-1.78494	-0.80675
H	3.737499	-3.49239	-0.47166
C	4.363357	-2.87818	4.944721
H	3.978196	-2.39477	5.855038
H	5.440981	-2.66142	4.869855
H	4.266058	-3.96914	5.083741
N	4.399848	4.466269	-0.56251
N	5.393068	5.311976	-0.6597
C	6.773965	4.885712	-0.76801
H	7.417932	5.7706	-0.82692
H	6.930293	4.27024	-1.67179
H	7.071564	4.286943	0.110351
C	5.065566	6.72457	-0.71379
H	5.356615	7.163727	-1.68356
H	5.583029	7.278109	0.087143
H	3.982181	6.826497	-0.58298

Cartesian coordinates of 3 (B3LYP-D3(BJ)/def2-SVP, MeCN, PCM model) in S_0 state

E = -2604.37937 Hartrees

Symbol	X	Y	Z
C	2.180026	0.32573	-0.28699
N	-0.04601	-0.56821	0.051136
S	-1.69046	1.42837	-0.01092
O	-4.28805	2.393504	0.071952
B	1.486556	-1.13566	-0.04789
C	-5.11059	1.490113	0.134809
S	1.826675	2.958366	-0.28769
O	-6.36034	-1.86792	0.311764
C	-6.59493	1.662694	0.179688

C	-7.34443	2.836004	0.159354
H	-6.85325	3.809441	0.10368
C	-8.73966	2.72298	0.212287
H	-9.35691	3.62396	0.197976
C	-9.36067	1.464667	0.283782
H	-10.4507	1.407766	0.323898
C	-8.6022	0.287031	0.304188
H	-9.07591	-0.69504	0.359712
C	-7.21596	0.404272	0.251246
C	-6.17049	-0.66416	0.256879
C	-4.8613	0.036536	0.181881
C	-1.2642	-1.12938	0.097559
H	-1.37138	-2.21182	0.13949
C	-2.32863	-0.23431	0.094339
C	-3.68176	-0.65449	0.164135
H	-3.79801	-1.74464	0.211774
C	-0.08653	0.789419	-0.05059
C	1.199202	1.333635	-0.1975
C	3.462398	0.903758	-0.39164
H	4.396271	0.345024	-0.45755
C	3.453575	2.300495	-0.42108
C	4.586077	3.172576	-0.54439
H	5.571212	2.696577	-0.61594
C	1.378411	-2.27389	-1.21861
C	1.542488	-1.97177	-2.59795
C	1.409388	-2.9732	-3.5702
H	1.54952	-2.70778	-4.62286
C	1.087662	-4.29409	-3.24149
C	0.875686	-4.57965	-1.89218
H	0.592556	-5.59677	-1.60356
C	1.010034	-3.60519	-0.88788
C	1.849696	-0.57761	-3.09767
H	2.834879	-0.22437	-2.7595
H	1.117185	0.160715	-2.7405
H	1.843835	-0.55127	-4.19712
C	0.975887	-5.35795	-4.30443
H	1.97304	-5.67683	-4.65477
H	0.430562	-4.98972	-5.18808
H	0.457094	-6.25221	-3.92799
C	0.757726	-4.07883	0.530015
H	0.162831	-5.00427	0.522026
H	0.231532	-3.34291	1.150187
H	1.700756	-4.29214	1.057123
C	2.173803	-1.60822	1.367688
C	3.447708	-2.23773	1.278024
C	4.136469	-2.62753	2.434555

H	5.111643	-3.11365	2.327216
C	3.621439	-2.41416	3.716827
C	2.39128	-1.76054	3.804322
H	1.972307	-1.55182	4.79357
C	1.672962	-1.34698	2.669045
C	0.371433	-0.62127	2.934135
H	-0.50363	-1.17888	2.567231
H	0.343418	0.371893	2.462686
H	0.23023	-0.47409	4.014262
C	4.121205	-2.55397	-0.04188
H	5.205946	-2.67134	0.100081
H	3.957618	-1.78619	-0.80672
H	3.737177	-3.49348	-0.47013
C	4.365038	-2.8742	4.945498
H	3.978831	-2.39147	5.855724
H	5.442266	-2.65545	4.870724
H	4.26985	-3.96533	5.084527
N	4.400348	4.465276	-0.56451
N	5.393254	5.310783	-0.66371
C	6.774299	4.884609	-0.77142
H	7.417863	5.769519	-0.83385
H	6.929863	4.266318	-1.67328
H	7.072726	4.288758	0.10869
C	5.066567	6.72391	-0.70956
H	5.365027	7.169604	-1.67393
H	5.5784	7.271571	0.099102
H	3.982308	6.825323	-0.58583

Cartesian coordinates of 2 (B3LYP-D3(BJ)/def2-SVP, *n*-hexane, PCM model) in S_1 state

E = -2986.026246 Hartrees

Symbol	X	Y	Z
C	0.73994	-1.07703	-0.18631
N	-1.66447	-0.98082	0.069095
S	-2.33733	1.515586	0.057942
O	-4.29001	3.461249	0.119988
B	-0.49826	-2.1302	-0.0305
C	-5.41515	2.986371	0.131397
S	1.502386	1.463178	-0.05613
O	-7.96006	0.455207	0.148687
C	-6.69356	3.76143	0.155793
C	-6.88194	5.141211	0.169514
H	-6.02387	5.816257	0.1625
C	-8.19661	5.621673	0.192203
H	-8.37984	6.698621	0.203396
C	-9.28933	4.737983	0.200788
H	-10.3039	5.142747	0.218482

C	-9.09496	3.351541	0.186918
H	-9.93504	2.654157	0.19321
C	-7.78577	2.878114	0.164371
C	-7.28644	1.468174	0.145913
C	-5.79884	1.559381	0.124124
C	-3.00887	-0.98975	0.062571
H	-3.5509	-1.93362	0.048657
C	-3.60607	0.264283	0.076814
C	-5.01706	0.442332	0.100544
H	-5.58007	-0.50008	0.099347
C	-1.14261	0.271585	0.026459
C	0.263252	0.240372	-0.06902
C	2.153227	-1.07524	-0.22623
H	2.775177	-1.96862	-0.28866
C	2.714554	0.198545	-0.18226
C	4.116015	0.524741	-0.22527
H	4.819252	-0.31642	-0.28792
C	-1.03451	-3.07556	-1.25113
C	-0.71026	-2.82803	-2.61201
C	-1.22574	-3.64325	-3.6283
H	-0.95052	-3.42944	-4.6659
C	-2.09086	-4.70873	-3.36245
C	-2.4467	-4.92248	-2.03094
H	-3.14334	-5.73201	-1.79165
C	-1.94287	-4.13362	-0.98303
C	0.182527	-1.68599	-3.04315
C	-2.61178	-5.58551	-4.47297
C	-2.41264	-4.5055	0.409911
C	-0.11271	-2.89488	1.369624
C	0.779791	-3.99804	1.265259
C	1.202496	-4.68424	2.411221
H	1.884506	-5.53267	2.29406
C	0.784727	-4.32129	3.694174
C	-0.05466	-3.2114	3.797364
H	-0.37499	-2.88029	4.790027
C	-0.49381	-2.4914	2.674661
C	-1.36486	-1.286	2.953995
C	1.299473	-4.51783	-0.05959
C	1.218857	-5.10064	4.909668
N	4.491066	1.7625	-0.18575
N	5.778511	2.110296	-0.21448
C	6.814021	1.118825	-0.28264
C	8.786748	-0.84538	-0.41576
C	7.323627	0.56813	0.897532
C	7.28087	0.689674	-1.52904
C	8.271125	-0.29341	-1.59278

C	8.313034	-0.41531	0.827866
C	6.093633	3.486655	-0.17775
C	6.735838	6.225292	-0.10495
C	7.437102	3.901602	-0.18786
C	5.072218	4.453192	-0.13064
C	5.400434	5.806951	-0.09458
C	7.74649	5.26244	-0.1518
H	8.238472	3.164298	-0.22327
H	8.79551	5.568321	-0.16012
H	6.983923	7.288309	-0.07683
H	4.596522	6.546407	-0.05817
H	4.03438	4.1262	-0.12285
H	6.942475	0.917768	1.858846
H	8.716147	-0.84574	1.74716
H	9.561255	-1.61387	-0.46778
H	8.641731	-0.62854	-2.56401
H	6.867176	1.133505	-2.43653
H	0.238366	-1.63171	-4.14017
H	1.20862	-1.79469	-2.6617
H	-0.18099	-0.71408	-2.67886
H	1.506887	-3.72348	-0.78627
H	0.567911	-5.18285	-0.54511
H	-3.34245	-5.09136	0.354421
H	-2.59483	-3.63976	1.058722
H	-3.46355	-6.19697	-4.13945
H	-1.83011	-6.27587	-4.83502
H	-2.93749	-4.98811	-5.33965
H	-1.66464	-5.11566	0.939682
H	2.224887	-5.09367	0.092041
H	0.601547	-6.00631	5.044336
H	2.26479	-5.43504	4.822785
H	1.125686	-4.50182	5.82837
H	-1.4674	-1.13204	4.037514
H	-2.38149	-1.39488	2.546363
H	-0.94289	-0.36171	2.532517

Cartesian coordinates of 2 (B3LYP-D3(BJ)/def2-SVP, MCH, PCM model) in S_1 state

E = -2986.027227 Hartrees

Symbol	X	Y	Z
C	0.740035	-1.07704	-0.18593
N	-1.66449	-0.98085	0.069054
S	-2.33731	1.515506	0.058283
O	-4.29052	3.461427	0.119837
B	-0.4983	-2.13017	-0.03044
C	-5.41565	2.986318	0.131326
S	1.502499	1.46312	-0.05596

O	-7.95995	0.45461	0.149075
C	-6.69414	3.761136	0.155543
C	-6.88283	5.140874	0.168956
H	-6.02507	5.816294	0.1618
C	-8.19764	5.621062	0.191525
H	-8.38108	6.697961	0.202474
C	-9.29016	4.737136	0.200294
H	-10.3048	5.141658	0.217885
C	-9.09548	3.350707	0.18673
H	-9.93558	2.653358	0.193166
C	-7.78618	2.87755	0.164301
C	-7.28647	1.467855	0.146123
C	-5.79899	1.559258	0.124319
C	-3.00889	-0.98979	0.062658
H	-3.55109	-1.93356	0.048889
C	-3.60606	0.264265	0.077029
C	-5.01701	0.442265	0.100838
H	-5.57974	-0.50028	0.099763
C	-1.14256	0.271581	0.026634
C	0.263296	0.24036	-0.0687
C	2.153315	-1.07534	-0.22589
H	2.775368	-1.96863	-0.28852
C	2.714709	0.198461	-0.18199
C	4.116132	0.524686	-0.22503
H	4.819472	-0.31635	-0.28762
C	-1.03389	-3.07541	-1.25155
C	-0.70926	-2.82735	-2.61229
C	-1.22395	-3.64258	-3.62903
H	-0.94846	-3.42834	-4.66647
C	-2.08863	-4.70863	-3.36383
C	-2.44492	-4.92286	-2.03247
H	-3.14131	-5.73274	-1.79366
C	-1.94189	-4.13398	-0.98416
C	0.183044	-1.68473	-3.04291
C	-2.60859	-5.5855	-4.47474
C	-2.41221	-4.50634	0.408456
C	-0.11323	-2.89506	1.369782
C	0.779655	-3.99798	1.265734
C	1.202229	-4.68412	2.411812
H	1.884575	-5.53231	2.294834
C	0.783917	-4.32137	3.694684
C	-0.05594	-3.21177	3.797596
H	-0.37677	-2.88084	4.79016
C	-0.49499	-2.49184	2.674739
C	-1.36672	-1.28689	2.953955
C	1.299923	-4.51761	-0.05895

C	1.218067	-5.10055	4.910293
N	4.490956	1.762571	-0.18558
N	5.778312	2.110476	-0.2143
C	6.814042	1.119093	-0.28224
C	8.787209	-0.84459	-0.41489
C	7.323684	0.568821	0.898109
C	7.280964	0.689862	-1.52859
C	8.271478	-0.29299	-1.59207
C	8.313354	-0.41439	0.828653
C	6.093225	3.486835	-0.17767
C	6.735202	6.225647	-0.1051
C	7.43668	3.901948	-0.18773
C	5.071733	4.453358	-0.13074
C	5.399808	5.807188	-0.0948
C	7.745953	5.262831	-0.15179
H	8.238121	3.164723	-0.22303
H	8.794952	5.568778	-0.16007
H	6.983199	7.288689	-0.07708
H	4.595821	6.54657	-0.05852
H	4.033883	4.126396	-0.123
H	6.942451	0.918498	1.859373
H	8.716547	-0.84451	1.748046
H	9.561924	-1.61287	-0.46673
H	8.64219	-0.62823	-2.56322
H	6.867215	1.133332	-2.43623
H	0.23895	-1.63002	-4.13991
H	1.20914	-1.79312	-2.66139
H	-0.18098	-0.71315	-2.67825
H	1.507133	-3.72319	-0.78559
H	0.568726	-5.18303	-0.54447
H	-3.34178	-5.09251	0.352375
H	-2.59508	-3.64077	1.057268
H	-3.4613	-6.19618	-4.1422
H	-1.8269	-6.27663	-4.83529
H	-2.93246	-4.98825	-5.3422
H	-1.6642	-5.1164	0.938359
H	2.225554	-5.09302	0.092928
H	0.602521	-6.00762	5.043546
H	2.264804	-5.43274	4.824565
H	1.122398	-4.5025	5.829222
H	-1.46956	-1.13307	4.037465
H	-2.38319	-1.39634	2.546106
H	-0.9452	-0.36235	2.532601

Cartesian coordinates of 2 (B3LYP-D3(BJ)/def2-SVP, toluene, PCM model) in S_1 state

E = -2986.029243 Hartrees

Symbol	X	Y	Z
C	0.740218	-1.07706	-0.18511
N	-1.66454	-0.98093	0.068945
S	-2.33729	1.515322	0.058879
O	-4.29158	3.461775	0.119503
B	-0.49838	-2.1301	-0.03026
C	-5.41671	2.986191	0.131118
S	1.502713	1.462989	-0.05561
O	-7.95975	0.453382	0.149629
C	-6.69532	3.76052	0.154996
C	-6.88468	5.140168	0.167856
H	-6.02755	5.816366	0.160458
C	-8.19976	5.619792	0.190189
H	-8.38365	6.696588	0.200697
C	-9.29188	4.735383	0.199272
H	-10.3067	5.139406	0.216665
C	-9.09656	3.34898	0.18626
H	-9.9367	2.651702	0.192943
C	-7.78704	2.876381	0.164061
C	-7.28654	1.467188	0.146388
C	-5.79929	1.558993	0.124588
C	-3.00894	-0.98991	0.062774
H	-3.55149	-1.93347	0.049321
C	-3.60607	0.264209	0.077363
C	-5.01693	0.442109	0.101304
H	-5.57911	-0.5007	0.10046
C	-1.14248	0.271562	0.026957
C	0.263373	0.240322	-0.06803
C	2.153489	-1.07555	-0.22514
H	2.775758	-1.96867	-0.28821
C	2.715014	0.198276	-0.18141
C	4.116365	0.524566	-0.22453
H	4.819912	-0.31622	-0.28702
C	-1.03259	-3.07517	-1.25235
C	-0.70715	-2.8261	-2.61279
C	-1.22021	-3.64139	-3.63044
H	-0.94411	-3.4263	-4.66755
C	-2.08403	-4.70854	-3.36653
C	-2.44126	-4.9237	-2.03551
H	-3.13717	-5.7343	-1.79765
C	-1.93989	-4.13474	-0.98638
C	0.184208	-1.68231	-3.04237
C	-2.60204	-5.58558	-4.47826
C	-2.41134	-4.50806	0.405574

C	-0.11431	-2.89536	1.37019
C	0.779426	-3.99775	1.266851
C	1.201768	-4.68368	2.413192
H	1.884863	-5.53134	2.296634
C	0.782303	-4.3213	3.69589
C	-0.05864	-3.21241	3.798172
H	-0.38055	-2.88184	4.790509
C	-0.4975	-2.49268	2.674949
C	-1.37076	-1.28874	2.953873
C	1.300927	-4.51709	-0.05746
C	1.216701	-5.10003	4.911743
N	4.490722	1.762709	-0.18524
N	5.7779	2.11084	-0.21396
C	6.81408	1.119644	-0.28154
C	8.78817	-0.84293	-0.41334
C	7.323968	0.570344	0.899156
C	7.280993	0.690151	-1.52779
C	8.272044	-0.29223	-1.59081
C	8.314188	-0.41238	0.83008
C	6.092383	3.487199	-0.17752
C	6.733886	6.226375	-0.10533
C	7.435806	3.902665	-0.18764
C	5.070733	4.453686	-0.13075
C	5.398518	5.807663	-0.09499
C	7.74484	5.263637	-0.15188
H	8.237395	3.165612	-0.22284
H	8.793794	5.569726	-0.1602
H	6.981703	7.289466	-0.07745
H	4.594377	6.546891	-0.05884
H	4.032858	4.126787	-0.123
H	6.942693	0.920173	1.860341
H	8.717678	-0.8418	1.749658
H	9.563316	-1.61079	-0.46485
H	8.642841	-0.62776	-2.56181
H	6.866999	1.132794	-2.43572
H	0.240291	-1.62679	-4.13931
H	1.210292	-1.7901	-2.66068
H	-0.18085	-0.7114	-2.677
H	1.507601	-3.72258	-0.7841
H	0.570538	-5.18348	-0.54291
H	-3.34039	-5.09488	0.348286
H	-2.59563	-3.6428	1.05435
H	-3.4561	-6.1953	-4.14747
H	-1.82008	-6.27767	-4.8364
H	-2.92299	-4.98855	-5.34694
H	-1.66333	-5.11788	0.935772

H	2.227072	-5.0915	0.094934
H	0.606871	-6.01157	5.040531
H	2.265985	-5.42505	4.829581
H	1.113215	-4.50449	5.831426
H	-1.47426	-1.13519	4.037357
H	-2.38688	-1.39946	2.54555
H	-0.95027	-0.36364	2.532767

Cartesian coordinates of 2 (B3LYP-D3(BJ)/def2-SVP, $CHCl_3$, PCM model) in S_1 state

E = -2986.035951 Hartrees

Symbol	X	Y	Z
C	0.740706	-1.07728	-0.18163
N	-1.66488	-0.98127	0.068899
S	-2.33745	1.514518	0.06015
O	-4.29514	3.462786	0.118138
B	-0.49883	-2.13003	-0.02885
C	-5.42029	2.985671	0.129639
S	1.503316	1.46237	-0.0538
O	-7.95933	0.449399	0.148654
C	-6.69929	3.758452	0.152333
C	-6.89084	5.137803	0.163952
H	-6.03586	5.816654	0.156381
C	-8.20684	5.61556	0.185297
H	-8.39221	6.692002	0.194802
C	-9.29765	4.72957	0.194649
H	-10.313	5.131972	0.211254
C	-9.10019	3.343267	0.182885
H	-9.94043	2.646184	0.18981
C	-7.78995	2.872522	0.161655
C	-7.28696	1.464984	0.145214
C	-5.80045	1.558039	0.124042
C	-3.00935	-0.99045	0.062946
H	-3.55295	-1.93338	0.050718
C	-3.60628	0.263845	0.077685
C	-5.01689	0.441477	0.101594
H	-5.57735	-0.50212	0.101343
C	-1.14244	0.271375	0.028219
C	0.263523	0.240025	-0.06516
C	2.154015	-1.07643	-0.22194
H	2.776998	-1.96891	-0.28682
C	2.71596	0.197485	-0.17888
C	4.117091	0.524053	-0.22271
H	4.821313	-0.31585	-0.28564
C	-1.02818	-3.07484	-1.254
C	-0.69934	-2.82317	-2.61346
C	-1.2068	-3.63901	-3.63391

H	-0.92808	-3.42182	-4.66989
C	-2.06836	-4.70935	-3.37414
C	-2.42936	-4.92685	-2.04428
H	-3.124	-5.73944	-1.80948
C	-1.93352	-4.13734	-0.99259
C	0.189547	-1.67612	-3.03974
C	-2.58024	-5.58669	-4.48859
C	-2.40913	-4.51328	0.39716
C	-0.11815	-2.89609	1.37262
C	0.778992	-3.9965	1.272009
C	1.200886	-4.68112	2.419376
H	1.886874	-5.52668	2.304527
C	0.777356	-4.31968	3.701472
C	-0.06806	-3.21386	3.801251
H	-0.39418	-2.88472	4.792682
C	-0.50654	-2.49522	2.676485
C	-1.38605	-1.29539	2.954109
C	1.304654	-4.51517	-0.05091
C	1.214904	-5.09557	4.918151
N	4.489828	1.763045	-0.18371
N	5.776454	2.112082	-0.2132
C	6.814152	1.121695	-0.28125
C	8.79158	-0.83678	-0.41353
C	7.327446	0.576189	0.89972
C	7.27861	0.69092	-1.52797
C	8.271536	-0.28969	-1.59113
C	8.319641	-0.40465	0.830267
C	6.08938	3.488417	-0.17625
C	6.729079	6.228863	-0.10302
C	7.432615	3.905302	-0.18879
C	5.067172	4.454612	-0.1266
C	5.393877	5.809099	-0.0904
C	7.740744	5.266574	-0.1525
H	8.234764	3.169037	-0.22641
H	8.789494	5.573255	-0.16275
H	6.976213	7.292109	-0.07473
H	4.589206	6.547655	-0.05203
H	4.02926	4.127818	-0.11712
H	6.947936	0.926822	1.861283
H	8.726155	-0.83123	1.749788
H	9.568242	-1.60306	-0.46528
H	8.640649	-0.6265	-2.56228
H	6.861806	1.130295	-2.43616
H	0.246628	-1.6184	-4.13651
H	1.215412	-1.78232	-2.6571
H	-0.17863	-0.70713	-2.67253

H	1.508974	-3.7206	-0.778
H	0.577198	-5.18547	-0.53553
H	-3.33679	-5.10173	0.335866
H	-2.59781	-3.6489	1.045658
H	-3.43364	-6.19914	-4.16122
H	-1.79512	-6.27615	-4.84481
H	-2.89929	-4.98968	-5.35794
H	-1.66145	-5.12274	0.92835
H	2.232839	-5.08564	0.103272
H	0.647155	-6.03744	5.016291
H	2.280518	-5.36925	4.860647
H	1.0581	-4.51961	5.842565
H	-1.4921	-1.14274	4.037462
H	-2.40078	-1.41136	2.544009
H	-0.96985	-0.36806	2.53379

Cartesian coordinates of 2 (B3LYP-D3(BJ)/def2-SVP, THF, PCM model) in S_1 state

E = -2986.038906 Hartrees

Symbol	X	Y	Z
C	0.740948	-1.07743	-0.17938
N	-1.66504	-0.98136	0.069156
S	-2.33746	1.514198	0.060277
O	-4.2965	3.463345	0.116945
B	-0.49907	-2.13	-0.02771
C	-5.42169	2.985635	0.127942
S	1.503733	1.461955	-0.05207
O	-7.95918	0.448046	0.14575
C	-6.70081	3.757827	0.149821
C	-6.89327	5.137056	0.161111
H	-6.03924	5.817077	0.153931
C	-8.20968	5.61404	0.181636
H	-8.39566	6.690326	0.190862
C	-9.29995	4.727406	0.190518
H	-10.3155	5.129149	0.206491
C	-9.1016	3.341147	0.179092
H	-9.94189	2.64415	0.18568
C	-7.79105	2.871175	0.158682
C	-7.28707	1.464333	0.142709
C	-5.80087	1.557839	0.122405
C	-3.00957	-0.9906	0.062819
H	-3.55364	-1.93324	0.051156
C	-3.60634	0.263787	0.077197
C	-5.01688	0.441375	0.100609
H	-5.57668	-0.50251	0.100474
C	-1.14241	0.27131	0.029056
C	0.263682	0.239825	-0.06318

C	2.154316	-1.07696	-0.21974
H	2.777569	-1.96917	-0.28565
C	2.716502	0.196946	-0.17688
C	4.117563	0.523634	-0.22123
H	4.822058	-0.31586	-0.2851
C	-1.02613	-3.07469	-1.25425
C	-0.69502	-2.82241	-2.61324
C	-1.19979	-3.63859	-3.63489
H	-0.91926	-3.42099	-4.67029
C	-2.06103	-4.70988	-3.37701
C	-2.42437	-4.92791	-2.04779
H	-3.11881	-5.74106	-1.81443
C	-1.93109	-4.13804	-0.99495
C	0.193578	-1.6745	-3.03793
C	-2.57064	-5.58675	-4.49294
C	-2.40912	-4.51481	0.393714
C	-0.12018	-2.89656	1.374237
C	0.778346	-3.99617	1.274923
C	1.1998	-4.68033	2.422806
H	1.887041	-5.52499	2.308798
C	0.77424	-4.31939	3.704562
C	-0.07314	-3.21487	3.803163
H	-0.40131	-2.88644	4.794149
C	-0.51109	-2.49653	2.677699
C	-1.39337	-1.29851	2.9546
C	1.306081	-4.51455	-0.04729
C	1.212686	-5.09411	4.921717
N	4.48956	1.762981	-0.18188
N	5.775946	2.11253	-0.2122
C	6.814325	1.122649	-0.28209
C	8.79336	-0.83369	-0.41778
C	7.331015	0.578778	0.898146
C	7.275892	0.691475	-1.52975
C	8.269686	-0.2882	-1.59458
C	8.324158	-0.40104	0.826934
C	6.088099	3.488836	-0.17387
C	6.726834	6.229852	-0.09787
C	7.43118	3.906529	-0.18916
C	5.06563	4.454745	-0.12011
C	5.391776	5.809457	-0.0826
C	7.738829	5.267923	-0.15146
H	8.233608	3.170773	-0.23009
H	8.787439	5.574972	-0.16392
H	6.973606	7.293153	-0.0685
H	4.586863	6.547585	-0.04105
H	4.027745	4.127899	-0.10857

H	6.953703	0.929629	1.860482
H	8.733492	-0.82637	1.745764
H	9.570733	-1.59914	-0.47091
H	8.636605	-0.62544	-2.56638
H	6.856389	1.129395	-2.43739
H	0.251564	-1.61605	-4.1346
H	1.219139	-1.78054	-2.65446
H	-0.17559	-0.70609	-2.67021
H	1.509363	-3.72001	-0.77462
H	0.580061	-5.18676	-0.53149
H	-3.33672	-5.10306	0.330457
H	-2.59897	-3.65076	1.042203
H	-3.41629	-6.20889	-4.16385
H	-1.78087	-6.26657	-4.85717
H	-2.9001	-4.98853	-5.35761
H	-1.66222	-5.12499	0.925247
H	2.235218	-5.08313	0.107834
H	0.660213	-6.04599	5.009335
H	2.283253	-5.34981	4.873224
H	1.037316	-4.5255	5.847282
H	-1.5007	-1.14623	4.037872
H	-2.40742	-1.41683	2.543588
H	-0.97907	-0.37021	2.534611

Cartesian coordinates of 2 (B3LYP-D3(BJ)/def2-SVP, CH_2Cl_2, PCM model) in S_1 state

E = -2986.039833 Hartrees

Symbol	X	Y	Z
C	0.741013	-1.0775	-0.1786
N	-1.66507	-0.98136	0.069548
S	-2.33743	1.514119	0.060453
O	-4.29686	3.46356	0.116793
B	-0.49915	-2.12998	-0.0271
C	-5.42206	2.985682	0.127769
S	1.503896	1.461804	-0.05163
O	-7.95911	0.447725	0.145544
C	-6.70121	3.75771	0.149486
C	-6.89394	5.136905	0.160638
H	-6.0402	5.817287	0.153474
C	-8.21046	5.613661	0.181014
H	-8.39663	6.689899	0.19013
C	-9.30058	4.726839	0.189887
H	-10.3162	5.12839	0.205744
C	-9.10196	3.340592	0.178603
H	-9.94227	2.643624	0.185191
C	-7.79133	2.870848	0.158344
C	-7.28707	1.464216	0.142529

C	-5.80096	1.557839	0.122357
C	-3.00963	-0.99062	0.063194
H	-3.55385	-1.93316	0.05184
C	-3.60634	0.263807	0.077399
C	-5.01685	0.441397	0.10077
H	-5.57645	-0.50257	0.100794
C	-1.14239	0.271304	0.029494
C	0.26375	0.239754	-0.06248
C	2.1544	-1.07716	-0.21915
H	2.777721	-1.96929	-0.28546
C	2.71668	0.196738	-0.17648
C	4.117719	0.523454	-0.22122
H	4.822283	-0.31592	-0.2856
C	-1.02564	-3.07469	-1.25397
C	-0.69386	-2.82235	-2.61287
C	-1.1979	-3.63871	-3.63478
H	-0.91681	-3.42111	-4.67004
C	-2.05913	-4.71019	-3.37735
C	-2.42314	-4.92824	-2.04831
H	-3.11757	-5.7415	-1.81528
C	-1.93055	-4.1382	-0.9952
C	0.194735	-1.6743	-3.0372
C	-2.56827	-5.58686	-4.49368
C	-2.40925	-4.51511	0.39319
C	-0.12071	-2.89668	1.374974
C	0.778191	-3.99607	1.276009
C	1.199578	-4.6801	2.424035
H	1.887168	-5.5245	2.310249
C	0.7735	-4.31928	3.705711
C	-0.07443	-3.21511	3.803999
H	-0.40315	-2.88687	4.794866
C	-0.51227	-2.49686	2.678343
C	-1.39533	-1.29936	2.955059
C	1.306447	-4.51442	-0.04602
C	1.212217	-5.09366	4.923002
N	4.489492	1.762909	-0.18176
N	5.775798	2.112631	-0.21261
C	6.814376	1.122927	-0.28351
C	8.793898	-0.83271	-0.42109
C	7.332647	0.579593	0.896282
C	7.274515	0.691619	-1.53165
C	8.268569	-0.28774	-1.59741
C	8.32608	-0.39988	0.824108
C	6.087708	3.488922	-0.17369
C	6.72614	6.230111	-0.09651
C	7.430726	3.906894	-0.19037

C	5.065172	4.454716	-0.11797
C	5.391142	5.809496	-0.0799
C	7.738224	5.268322	-0.15206
H	8.233232	3.171323	-0.23288
H	8.786779	5.575501	-0.16563
H	6.972799	7.293425	-0.06668
H	4.586165	6.547469	-0.03683
H	4.027305	4.127841	-0.10539
H	6.95643	0.930519	1.859016
H	8.736703	-0.82479	1.742549
H	9.571486	-1.59788	-0.47497
H	8.634388	-0.62513	-2.56958
H	6.853771	1.129058	-2.43894
H	0.252942	-1.61572	-4.13385
H	1.220215	-1.78038	-2.65353
H	-0.17463	-0.70601	-2.66938
H	1.509355	-3.71992	-0.77347
H	0.580822	-5.18725	-0.52999
H	-3.33695	-5.10312	0.329428
H	-2.59922	-3.65112	1.041691
H	-3.41024	-6.21332	-4.16343
H	-1.77663	-6.26232	-4.86188
H	-2.90286	-4.98815	-5.35606
H	-1.66264	-5.12567	0.924737
H	2.235886	-5.08242	0.10934
H	0.663645	-6.04801	5.008034
H	2.283963	-5.34485	4.876753
H	1.032219	-4.52692	5.848808
H	-1.50293	-1.14713	4.038309
H	-2.40922	-1.4184	2.543889
H	-0.98165	-0.37079	2.535065

Cartesian coordinates of 2 (B3LYP-D3(BJ)/def2-SVP, acetone, PCM model) in S_1 state

E = -2986.043563 Hartrees

Symbol	X	Y	Z
C	0.741299	-1.07774	-0.17573
N	-1.665162	-0.9813	0.070546
S	-2.337203	1.513959	0.06026
O	-4.297673	3.464355	0.115039
B	-0.499399	-2.12993	-0.02514
C	-5.422959	2.986052	0.125467
S	1.504581	1.46122	-0.04938
O	-7.958876	0.447166	0.141797
C	-6.702147	3.75767	0.146262
C	-6.895631	5.13677	0.157085
H	-6.04273	5.8182	0.150415

C	-8.212491	5.612904	0.176515
H	-8.399152	6.689008	0.185356
C	-9.302192	4.725576	0.18479
H	-10.317999	5.126605	0.199921
C	-9.102835	3.339361	0.173848
H	-9.94321	2.642497	0.180003
C	-7.791962	2.870242	0.154547
C	-7.28695	1.464205	0.139274
C	-5.801093	1.55809	0.12015
C	-3.009815	-0.99053	0.063559
H	-3.554515	-1.93278	0.052995
C	-3.606252	0.264016	0.076871
C	-5.016708	0.441676	0.099519
H	-5.575799	-0.5025	0.0998
C	-1.142241	0.271311	0.030762
C	0.264092	0.239482	-0.0599
C	2.154773	-1.0779	-0.21654
H	2.778266	-1.96979	-0.28409
C	2.717389	0.195937	-0.1742
C	4.118387	0.522724	-0.21996
H	4.823153	-0.31624	-0.28629
C	-1.02391	-3.0746	-1.25315
C	-0.68946	-2.82243	-2.61162
C	-1.191136	-3.63927	-3.63445
H	-0.907856	-3.42194	-4.66917
C	-2.052839	-4.71098	-3.37858
C	-2.419552	-4.92876	-2.05016
H	-3.114351	-5.74204	-1.81834
C	-1.929128	-4.13827	-0.99615
C	0.19975	-1.67433	-3.03465
C	-2.560378	-5.58705	-4.49615
C	-2.410493	-4.51534	0.391252
C	-0.122771	-2.8972	1.377374
C	0.77707	-3.99612	1.279617
C	1.197787	-4.67992	2.428148
H	1.886324	-5.52365	2.315166
C	0.769776	-4.31953	3.70948
C	-0.079674	-3.2163	3.80668
H	-0.410337	-2.88872	4.79712
C	-0.516634	-2.49808	2.680392
C	-1.401987	-1.30205	2.95635
C	1.307222	-4.51433	-0.04172
C	1.208672	-5.09316	4.927244
N	4.489462	1.762487	-0.17963
N	5.775514	2.112824	-0.2121
C	6.814715	1.123777	-0.28716

C	8.795788	-0.82948	-0.43266
C	7.338865	0.581843	0.890671
C	7.269486	0.692391	-1.53724
C	8.264349	-0.28592	-1.60695
C	8.333221	-0.39645	0.814482
C	6.086628	3.489054	-0.17029
C	6.724015	6.230733	-0.08728
C	7.429385	3.908014	-0.1922
C	5.063894	4.454342	-0.10642
C	5.389268	5.80931	-0.06559
C	7.736363	5.269524	-0.15094
H	8.232129	3.173136	-0.2412
H	8.784701	5.577201	-0.16871
H	6.970285	7.294069	-0.05518
H	4.584105	6.546688	-0.0162
H	4.026133	4.127271	-0.08984
H	6.966787	0.932745	1.855009
H	8.748603	-0.82026	1.731272
H	9.57405	-1.59372	-0.4897
H	8.626004	-0.62348	-2.58058
H	6.844145	1.128538	-2.44299
H	0.259091	-1.61549	-4.13122
H	1.224788	-1.78092	-2.64996
H	-0.169967	-0.70623	-2.66675
H	1.509401	-3.71994	-0.76942
H	0.58282	-5.18886	-0.52526
H	-3.338889	-5.10197	0.325714
H	-2.600404	-3.65147	1.039823
H	-3.392425	-6.22514	-4.16315
H	-1.763753	-6.25061	-4.87491
H	-2.908552	-4.98713	-5.35235
H	-1.665332	-5.12761	0.922939
H	2.237414	-5.08075	0.114587
H	0.670599	-6.05403	5.005014
H	2.283458	-5.33228	4.887455
H	1.015909	-4.53164	5.853615
H	-1.510672	-1.14998	4.039508
H	-2.415295	-1.42318	2.544445
H	-0.990022	-0.37271	2.536438

Cartesian coordinates of 2 (B3LYP-D3(BJ)/def2-SVP, EtOH, PCM model) in S_1 state

E = -2986.042985 Hartrees

Symbol	X	Y	Z
C	0.741348	-1.07779	-0.17526
N	-1.66517	-0.98128	0.070738
S	-2.33715	1.513952	0.060175

O	-4.29775	3.464499	0.114675
B	-0.49944	-2.12992	-0.02479
C	-5.42305	2.986146	0.125023
S	1.504709	1.461121	-0.04902
O	-7.95883	0.447152	0.141146
C	-6.70224	3.757717	0.145668
C	-6.89582	5.136806	0.156429
H	-6.04303	5.818382	0.149833
C	-8.21272	5.61286	0.175706
H	-8.39945	6.688946	0.184496
C	-9.30237	4.725468	0.183892
H	-10.3182	5.126431	0.198904
C	-9.10292	3.339257	0.173013
H	-9.94331	2.64241	0.179103
C	-7.79201	2.870218	0.153868
C	-7.28691	1.464261	0.138694
C	-5.80109	1.558169	0.119736
C	-3.00984	-0.9905	0.063633
H	-3.55461	-1.9327	0.053208
C	-3.60622	0.264074	0.076762
C	-5.01667	0.441754	0.09928
H	-5.5757	-0.50244	0.099616
C	-1.14221	0.27132	0.030964
C	0.264161	0.239437	-0.05948
C	2.154838	-1.07802	-0.21611
H	2.778349	-1.96988	-0.28385
C	2.717512	0.195796	-0.17383
C	4.118506	0.522588	-0.21977
H	4.823296	-0.31631	-0.28647
C	-1.02367	-3.0746	-1.25295
C	-0.68878	-2.82252	-2.61136
C	-1.19013	-3.63944	-3.6343
H	-0.90649	-3.42221	-4.66894
C	-2.05198	-4.71113	-3.37863
C	-2.41913	-4.9288	-2.05031
H	-3.11403	-5.74203	-1.81865
C	-1.929	-4.13823	-0.99618
C	0.200596	-1.67448	-3.03422
C	-2.55934	-5.58709	-4.49638
C	-2.41079	-4.51526	0.391075
C	-0.12309	-2.89728	1.377792
C	0.776849	-3.99615	1.280216
C	1.197434	-4.67994	2.428826
H	1.886085	-5.52359	2.315967
C	0.769134	-4.31961	3.710101
C	-0.0805	-3.21648	3.807138

H	-0.41144	-2.88899	4.797513
C	-0.51729	-2.49824	2.680759
C	-1.40293	-1.3024	2.956591
C	1.30728	-4.51437	-0.04101
C	1.207959	-5.09317	4.927941
N	4.489482	1.762392	-0.17926
N	5.775495	2.112827	-0.21203
C	6.814781	1.123889	-0.28789
C	8.796076	-0.829	-0.43494
C	7.340014	0.582154	0.889546
C	7.268543	0.692508	-1.53834
C	8.263519	-0.28563	-1.60883
C	8.334504	-0.39595	0.812577
C	6.086492	3.489044	-0.16964
C	6.723724	6.230784	-0.08547
C	7.429199	3.908165	-0.19255
C	5.063739	4.454236	-0.10418
C	5.389026	5.809227	-0.0628
C	7.736101	5.269681	-0.1507
H	8.231971	3.173407	-0.24281
H	8.784399	5.577444	-0.16928
H	6.969938	7.29412	-0.05292
H	4.583843	6.546502	-0.01218
H	4.026001	4.127124	-0.08683
H	6.968717	0.93304	1.854189
H	8.750757	-0.81961	1.729042
H	9.574434	-1.59309	-0.49259
H	8.624388	-0.62321	-2.58274
H	6.842359	1.12847	-2.44378
H	0.260133	-1.61564	-4.13077
H	1.225554	-1.7812	-2.64935
H	-0.16912	-0.70637	-2.66634
H	1.509388	-3.72	-0.76875
H	0.583042	-5.18912	-0.5245
H	-3.33935	-5.10158	0.325285
H	-2.60059	-3.65139	1.039659
H	-3.38964	-6.22715	-4.16285
H	-1.76191	-6.2486	-4.87701
H	-2.90991	-4.98697	-5.35148
H	-1.6659	-5.12786	0.922773
H	2.237562	-5.08058	0.115444
H	0.670795	-6.0546	5.00504
H	2.282996	-5.33126	4.888778
H	1.014021	-4.53212	5.854341
H	-1.51177	-1.15033	4.039734
H	-2.41616	-1.42381	2.544588

H	-0.9912	-0.37296	2.536669

Cartesian coordinates of 2 (B3LYP-D3(BJ)/def2-SVP, MeCN, PCM model) in S_1 state

E = -2986.043559 Hartrees

Symbol	X	Y	Z
C	0.74142	-1.07786	-0.17454
N	-1.66518	-0.98124	0.071045
S	-2.33706	1.513946	0.060022
O	-4.29784	3.464714	0.114086
B	-0.49949	-2.1299	-0.02424
C	-5.42317	2.986295	0.124322
S	1.504901	1.460968	-0.04848
O	-7.95876	0.447155	0.140153
C	-6.70235	3.757803	0.144737
C	-6.89607	5.136875	0.155398
H	-6.04345	5.818661	0.148908
C	-8.21304	5.612817	0.174444
H	-8.39986	6.688877	0.183153
C	-9.30262	4.725336	0.182502
H	-10.3185	5.126205	0.197337
C	-9.10303	3.339129	0.171727
H	-9.94343	2.642309	0.177724
C	-7.79208	2.870204	0.152816
C	-7.28685	1.464361	0.137798
C	-5.80107	1.558298	0.119092
C	-3.00987	-0.99044	0.063756
H	-3.55476	-1.93257	0.053548
C	-3.60618	0.264165	0.076591
C	-5.01662	0.441879	0.098909
H	-5.57555	-0.50235	0.099338
C	-1.14215	0.271335	0.031271
C	0.264265	0.239366	-0.05885
C	2.154933	-1.07822	-0.21548
H	2.778469	-1.97003	-0.2835
C	2.717695	0.19558	-0.17328
C	4.118685	0.522378	-0.21951
H	4.823508	-0.31644	-0.28678
C	-1.02334	-3.0746	-1.25261
C	-0.6878	-2.82269	-2.61093
C	-1.18869	-3.63975	-3.63402
H	-0.90451	-3.4227	-4.66855
C	-2.05076	-4.71137	-3.37863
C	-2.41856	-4.92884	-2.05044
H	-3.11363	-5.74199	-1.819
C	-1.92884	-4.13815	-0.99616
C	0.201853	-1.67478	-3.03356

C	-2.55788	-5.58719	-4.49661
C	-2.41126	-4.5151	0.390899
C	-0.12356	-2.89739	1.378441
C	0.776526	-3.99621	1.281131
C	1.196917	-4.67997	2.429856
H	1.885725	-5.52351	2.317179
C	0.7682	-4.31972	3.711051
C	-0.0817	-3.21674	3.807847
H	-0.41307	-2.88938	4.798125
C	-0.51823	-2.49847	2.681328
C	-1.40433	-1.30291	2.956974
C	1.307346	-4.51443	-0.03994
C	1.206996	-5.09314	4.928996
N	4.489516	1.762241	-0.17871
N	5.775471	2.112823	-0.21193
C	6.81488	1.124053	-0.28908
C	8.796495	-0.82828	-0.43858
C	7.341803	0.582597	0.887731
C	7.26706	0.692696	-1.54011
C	8.262198	-0.2852	-1.61182
C	8.336485	-0.39522	0.809523
C	6.086297	3.489018	-0.16862
C	6.723303	6.230844	-0.08259
C	7.428925	3.908384	-0.19311
C	5.063523	4.454059	-0.10065
C	5.388681	5.809081	-0.05838
C	7.735715	5.269908	-0.15032
H	8.231732	3.17381	-0.24537
H	8.78395	5.577801	-0.17017
H	6.969433	7.294176	-0.04932
H	4.583475	6.546195	-0.00581
H	4.025824	4.126882	-0.08207
H	6.971729	0.933448	1.852855
H	8.754094	-0.81866	1.725472
H	9.574991	-1.59215	-0.4972
H	8.621833	-0.62279	-2.58618
H	6.839563	1.128394	-2.44506
H	0.261679	-1.61596	-4.13009
H	1.22669	-1.7817	-2.64843
H	-0.16785	-0.70663	-2.66574
H	1.509363	-3.72011	-0.76774
H	0.583334	-5.18951	-0.52334
H	-3.34008	-5.10094	0.324755
H	-2.60086	-3.65122	1.039496
H	-3.38569	-6.23007	-4.16232
H	-1.75933	-6.24576	-4.87993

H	-2.9119	-4.98681	-5.35013
H	-1.66679	-5.12821	0.922613
H	2.237751	-5.08036	0.116733
H	0.672052	-6.05592	5.004527
H	2.282646	-5.3287	4.891245
H	1.010342	-4.53323	5.855502
H	-1.51338	-1.15084	4.040092
H	-2.41745	-1.42476	2.544831
H	-0.99296	-0.37333	2.537028

Cartesian coordinates of 3 (B3LYP-D3(BJ)/def2-SVP, *n*-hexane, PCM model) in S_1 state

E = -2603.033566 Hartrees

Symbol	X	Y	Z
C	2.177937	0.329197	-0.28587
N	-0.044671	-0.56881	0.051604
S	-1.692732	1.424749	-0.00661
O	-4.278371	2.387365	0.075838
B	1.490311	-1.13438	-0.05201
C	-5.102765	1.488105	0.138042
S	1.819404	2.961261	-0.26644
O	-6.364718	-1.86741	0.307712
C	-6.587522	1.663905	0.186577
C	-7.332596	2.840276	0.171538
H	-6.833762	3.810012	0.117783
C	-8.727398	2.732139	0.227231
H	-9.341796	3.635463	0.217173
C	-9.352889	1.475727	0.296207
H	-10.443311	1.423006	0.338619
C	-8.599231	0.295737	0.311178
H	-9.072091	-0.68689	0.364602
C	-7.212721	0.408154	0.255448
C	-6.171885	-0.66696	0.255776
C	-4.858986	0.032175	0.180503
C	-1.263075	-1.13235	0.092517
H	-1.36525	-2.21566	0.124766
C	-2.327789	-0.23903	0.092046
C	-3.682815	-0.66029	0.159326
H	-3.803599	-1.75058	0.202059
C	-0.089002	0.786682	-0.04632
C	1.197685	1.334924	-0.19202
C	3.460107	0.913543	-0.38127
H	4.394704	0.355272	-0.44069
C	3.44513	2.30758	-0.40015
C	4.57926	3.185113	-0.5141
H	5.564469	2.705411	-0.57515
C	1.374882	-2.27122	-1.22124

C	1.537935	-1.97115	-2.60032
C	1.394766	-2.97167	-3.5707
H	1.534251	-2.70813	-4.62393
C	1.063884	-4.289	-3.23985
C	0.855329	-4.57272	-1.89034
H	0.567813	-5.58817	-1.60028
C	0.999936	-3.59953	-0.88716
C	1.85592	-0.57952	-3.09966
H	2.840813	-0.23033	-2.75612
H	1.125583	0.164081	-2.7488
H	1.856952	-0.55403	-4.19921
C	0.939163	-5.35165	-4.30225
H	1.931194	-5.65969	-4.67599
H	0.368561	-4.98773	-5.17186
H	0.437431	-6.25176	-3.9166
C	0.753186	-4.07112	0.53263
H	0.159406	-4.99756	0.528293
H	0.229419	-3.33515	1.155325
H	1.696875	-4.28117	1.059396
C	2.18347	-1.60924	1.358248
C	3.453219	-2.24418	1.259316
C	4.142519	-2.64449	2.411147
H	5.114227	-3.13625	2.297905
C	3.632543	-2.4362	3.695413
C	2.408251	-1.77424	3.791458
H	1.994769	-1.56724	4.783345
C	1.690056	-1.34912	2.661818
C	0.398079	-0.60934	2.932781
H	-0.485513	-1.1531	2.564974
H	0.383416	0.386844	2.466824
H	0.260319	-0.46527	4.013804
C	4.119955	-2.55564	-0.06517
H	5.204157	-2.68392	0.073372
H	3.961747	-1.78002	-0.8236
H	3.72694	-3.48652	-0.50354
C	4.374603	-2.91169	4.918798
H	4.016016	-2.40906	5.829744
H	5.457563	-2.72913	4.83066
H	4.243323	-3.9978	5.068482
N	4.398597	4.473294	-0.53287
N	5.400566	5.323308	-0.59938
C	6.773182	4.890518	-0.7521
H	7.428196	5.770143	-0.75901
H	6.921292	4.329055	-1.69443
H	7.073813	4.236455	0.084155
C	5.057394	6.716865	-0.80408

H	5.199915	7.025879	-1.85674
H	5.677049	7.365416	-0.16462
H	4.001759	6.847807	-0.53818

Cartesian coordinates of 3 (B3LYP-D3(BJ)/def2-SVP, MCH, PCM model) in S_1 state

E = -2603.034694 Hartrees

Symbol	X	Y	Z
C	2.178053	0.329467	-0.28532
N	-0.0447	-0.56873	0.051447
S	-1.69277	1.424846	-0.00653
O	-4.27906	2.387298	0.075762
B	1.490249	-1.13415	-0.0518
C	-5.10332	1.487761	0.137784
S	1.819395	2.961517	-0.26664
O	-6.36439	-1.86803	0.306976
C	-6.58807	1.663279	0.18616
C	-7.33348	2.839431	0.171145
H	-6.83515	3.80943	0.117531
C	-8.72829	2.730901	0.226682
H	-9.34291	3.634056	0.216634
C	-9.35344	1.474321	0.295487
H	-10.4438	1.421274	0.337782
C	-8.59943	0.294516	0.310434
H	-9.07225	-0.68813	0.363733
C	-7.21294	0.407316	0.254857
C	-6.17179	-0.66736	0.255178
C	-4.85914	0.031957	0.180119
C	-1.26303	-1.13227	0.09231
H	-1.36539	-2.21556	0.124662
C	-2.32783	-0.23897	0.091849
C	-3.68272	-0.66032	0.158972
H	-3.80314	-1.75063	0.201563
C	-0.08896	0.786916	-0.04627
C	1.197602	1.335152	-0.19172
C	3.46015	0.913705	-0.38085
H	4.39482	0.355582	-0.44042
C	3.445289	2.307883	-0.40011
C	4.57923	3.185304	-0.51429
H	5.564541	2.70596	-0.57542
C	1.375687	-2.2707	-1.22154
C	1.538976	-1.96999	-2.6005
C	1.397091	-2.97029	-3.57134
H	1.536745	-2.70623	-4.62442
C	1.067249	-4.28809	-3.24114
C	0.858258	-4.57243	-1.8918
H	0.571367	-5.58821	-1.60222

C	1.001565	-3.59944	-0.88819
C	1.855772	-0.57788	-3.09932
H	2.840451	-0.22809	-2.7558
H	1.124907	0.164945	-2.74794
H	1.856554	-0.55194	-4.19885
C	0.94397	-5.35049	-4.30397
H	1.936471	-5.65781	-4.67706
H	0.37387	-4.98661	-5.17393
H	0.442507	-6.25102	-3.91897
C	0.754221	-4.0717	0.531256
H	0.16103	-4.9985	0.5262
H	0.229512	-3.33626	1.153756
H	1.697751	-4.28134	1.058471
C	2.182778	-1.60947	1.3587
C	3.452798	-2.24401	1.260312
C	4.141614	-2.64457	2.412382
H	5.113552	-3.13596	2.299488
C	3.630853	-2.43694	3.696481
C	2.406249	-1.77543	3.792051
H	1.992112	-1.56898	4.783781
C	1.688514	-1.35011	2.66213
C	0.395996	-0.61112	2.932774
H	-0.4871	-1.15544	2.564625
H	0.380788	0.385082	2.466898
H	0.257829	-0.46728	4.013776
C	4.120381	-2.55486	-0.06389
H	5.204589	-2.68249	0.07513
H	3.962022	-1.77922	-0.82226
H	3.728116	-3.48597	-0.50244
C	4.372454	-2.91258	4.920112
H	4.012362	-2.41126	5.831184
H	5.455251	-2.72853	4.833081
H	4.24252	-3.999	5.068652
N	4.39809	4.47369	-0.53339
N	5.399487	5.323608	-0.60086
C	6.772698	4.891383	-0.75085
H	7.427026	5.771454	-0.75915
H	6.921918	4.327963	-1.69172
H	7.072705	4.239368	0.087232
C	5.056833	6.718185	-0.79994
H	5.204713	7.032229	-1.85026
H	5.673163	7.363708	-0.15422
H	3.999883	6.847433	-0.5386

Cartesian coordinates of 3 (B3LYP-D3(BJ)/def2-SVP, toluene, PCM model) in S_1 state

E = -2603.037023 Hartrees

Symbol	X	Y	Z
C	2.178319	0.329955	-0.28419
N	-0.044751	-0.56857	0.05108
S	-1.692823	1.42506	-0.00651
O	-4.280475	2.387241	0.075576
B	1.490113	-1.13375	-0.05134
C	-5.104439	1.487132	0.137239
S	1.819459	2.961977	-0.26717
O	-6.36372	-1.86922	0.305457
C	-6.589158	1.662079	0.185332
C	-7.33525	2.837787	0.170401
H	-6.837958	3.80832	0.11707
C	-8.730095	2.728462	0.225651
H	-9.345165	3.631276	0.215651
C	-9.354553	1.471542	0.294105
H	-10.444894	1.417834	0.336186
C	-8.599822	0.292109	0.308972
H	-9.072569	-0.69057	0.362011
C	-7.213389	0.405685	0.253675
C	-6.171583	-0.6681	0.253952
C	-4.859453	0.031588	0.179306
C	-1.262959	-1.1321	0.091864
H	-1.365706	-2.21534	0.124508
C	-2.327919	-0.23882	0.091376
C	-3.682526	-0.66032	0.158217
H	-3.802235	-1.75066	0.200561
C	-0.088856	0.787385	-0.04629
C	1.197475	1.335574	-0.19118
C	3.46029	0.913931	-0.37993
H	4.3951	0.356091	-0.43977
C	3.445699	2.308403	-0.40001
C	4.579271	3.185567	-0.51459
H	5.5648	2.706949	-0.57568
C	1.377318	-2.26968	-1.22214
C	1.54116	-1.96768	-2.60084
C	1.401808	-2.96755	-3.57264
H	1.541858	-2.70243	-4.6254
C	1.073947	-4.28624	-3.24375
C	0.864017	-4.57184	-1.89476
H	0.578298	-5.58824	-1.60619
C	1.004753	-3.59925	-0.89029
C	1.855704	-0.57466	-3.0986
H	2.839959	-0.22373	-2.75507
H	1.123814	0.166675	-2.74624

H	1.856062	-0.54782	-4.19811
C	0.953544	-5.34814	-4.30747
H	1.947007	-5.65437	-4.67887
H	0.384913	-4.98417	-5.17833
H	0.452165	-6.24932	-3.92391
C	0.756158	-4.07289	0.528456
H	0.16399	-5.00031	0.521933
H	0.229708	-3.33844	1.150601
H	1.699358	-4.28192	1.056517
C	2.181307	-1.60996	1.359662
C	3.451876	-2.2437	1.262438
C	4.13971	-2.64468	2.415029
H	5.112116	-3.13532	2.302887
C	3.627351	-2.4383	3.698772
C	2.402104	-1.77768	3.793317
H	1.986633	-1.57226	4.784711
C	1.6853	-1.35205	2.662789
C	0.391668	-0.61467	2.932727
H	-0.490403	-1.16017	2.5639
H	0.375331	0.381533	2.466959
H	0.252655	-0.47125	4.013675
C	4.121194	-2.55337	-0.06117
H	5.205404	-2.67973	0.078859
H	3.962577	-1.77775	-0.81944
H	3.730419	-3.48499	-0.50002
C	4.368002	-2.91412	4.922946
H	4.005154	-2.41509	5.834166
H	5.450516	-2.72749	4.837969
H	4.24042	-4.00108	5.069513
N	4.39715	4.474374	-0.53461
N	5.397394	5.324096	-0.60401
C	6.771851	4.893001	-0.74794
H	7.424791	5.773964	-0.75902
H	6.923624	4.325436	-1.68563
H	7.070279	4.245329	0.094091
C	5.055833	6.720649	-0.79193
H	5.215276	7.044781	-1.83724
H	5.664896	7.359962	-0.13324
H	3.996116	6.846558	-0.54059

Cartesian coordinates of 3 (B3LYP-D3(BJ)/def2-SVP, $CHCl_3$, PCM model) in S_1 state

E = -2603.044898 Hartrees

Symbol	X	Y	Z
C	2.179324	0.330092	-0.28244
N	-0.04508	-0.56817	0.049625
S	-1.69244	1.426121	-0.00872

O	-4.28474	2.38846	0.073649
B	1.489235	-1.13331	-0.04988
C	-5.10781	1.486555	0.135079
S	1.821036	2.962179	-0.27302
O	-6.36166	-1.87121	0.303237
C	-6.59239	1.659924	0.182551
C	-7.34047	2.834298	0.167148
H	-6.84641	3.806444	0.11384
C	-8.73543	2.722707	0.221911
H	-9.3518	3.624499	0.211508
C	-9.35784	1.464846	0.290362
H	-10.448	1.409228	0.332057
C	-8.60097	0.286482	0.305724
H	-9.07376	-0.69615	0.358808
C	-7.2147	0.402288	0.250926
C	-6.17089	-0.66871	0.251693
C	-4.86039	0.031913	0.177439
C	-1.26302	-1.13122	0.091285
H	-1.36738	-2.21421	0.126564
C	-2.32821	-0.23764	0.089919
C	-3.68197	-0.65914	0.156925
H	-3.79966	-1.74951	0.199897
C	-0.08816	0.788705	-0.04822
C	1.197607	1.336017	-0.19214
C	3.461175	0.912412	-0.37954
H	4.396146	0.35497	-0.44024
C	3.448215	2.307912	-0.40345
C	4.58091	3.183581	-0.51967
H	5.566977	2.706984	-0.58055
C	1.381237	-2.26855	-1.22273
C	1.546789	-1.96411	-2.60099
C	1.413784	-2.96343	-3.57475
H	1.555047	-2.69626	-4.62683
C	1.090661	-4.28423	-3.24853
C	0.878009	-4.5722	-1.9002
H	0.594844	-5.58989	-1.61364
C	1.012272	-3.60004	-0.89408
C	1.855959	-0.56918	-3.09702
H	2.83941	-0.21569	-2.75397
H	1.121831	0.168763	-2.74231
H	1.854917	-0.54081	-4.19647
C	0.977799	-5.34554	-4.31378
H	1.974005	-5.65285	-4.67686
H	0.417605	-4.98025	-5.18946
H	0.47226	-6.2463	-3.93475
C	0.760057	-4.0763	0.52306

H	0.169284	-5.00449	0.513233
H	0.230166	-3.34345	1.143969
H	1.702426	-4.28504	1.052837
C	2.176448	-1.61053	1.363293
C	3.44872	-2.24198	1.270357
C	4.134246	-2.64178	2.425024
H	5.108101	-3.13017	2.315711
C	3.617674	-2.43651	3.707558
C	2.39029	-1.77878	3.798208
H	1.971145	-1.57468	4.788345
C	1.675584	-1.35472	2.665259
C	0.378285	-0.62249	2.932295
H	-0.50041	-1.17255	2.562367
H	0.357809	0.373039	2.465395
H	0.23689	-0.47888	4.012904
C	4.122787	-2.55063	-0.05107
H	5.207012	-2.67292	0.091766
H	3.963153	-1.77646	-0.81045
H	3.736265	-3.48465	-0.48869
C	4.356376	-2.90986	4.933995
H	3.983825	-2.41782	5.845034
H	5.437521	-2.71297	4.855189
H	4.238667	-3.99855	5.075617
N	4.396006	4.473953	-0.54336
N	5.392669	5.322452	-0.62088
C	6.77121	4.894662	-0.74145
H	7.419646	5.778301	-0.76606
H	6.931774	4.308914	-1.6654
H	7.063702	4.2657	0.116847
C	5.056141	6.726154	-0.76319
H	5.268526	7.092323	-1.78396
H	5.629933	7.337813	-0.04856
H	3.98503	6.839757	-0.55969

Cartesian coordinates of 3 (B3LYP-D3(BJ)/def2-SVP, THF, PCM model) in S_1 state

E = -2603.048431 Hartrees

Symbol	X	Y	Z
C	2.179688	0.329128	-0.28316
N	-0.04534	-0.56809	0.049663
S	-1.691898	1.426869	-0.00958
O	-4.286277	2.389906	0.073095
B	1.488466	-1.13376	-0.04917
C	-5.10905	1.487313	0.134705
S	1.822653	2.961362	-0.27741
O	-6.360957	-1.87079	0.304549
C	-6.593553	1.660207	0.181454

C	-7.342324	2.834094	0.164923
H	-6.849509	3.806835	0.111178
C	-8.737372	2.721756	0.219129
H	-9.354183	3.623177	0.207823
C	-9.359084	1.463612	0.288144
H	-10.449177	1.407347	0.329369
C	-8.601456	0.285608	0.304665
H	-9.074466	-0.6969	0.358212
C	-7.215223	0.402157	0.250434
C	-6.170655	-0.66774	0.252339
C	-4.860754	0.03308	0.178074
C	-1.263285	-1.13061	0.092546
H	-1.368587	-2.21344	0.129777
C	-2.328348	-0.23663	0.090631
C	-3.681803	-0.65782	0.158267
H	-3.798813	-1.74815	0.202349
C	-0.087641	0.789142	-0.04911
C	1.198015	1.335572	-0.19353
C	3.461635	0.910152	-0.38223
H	4.396385	0.352486	-0.44429
C	3.449942	2.306133	-0.40809
C	4.582455	3.180656	-0.52622
H	5.568396	2.704523	-0.5895
C	1.381324	-2.26955	-1.22193
C	1.546769	-1.96516	-2.60036
C	1.41485	-2.96484	-3.57415
H	1.555998	-2.69766	-4.62624
C	1.092957	-4.28605	-3.24786
C	0.880046	-4.57394	-1.89942
H	0.597378	-5.59175	-1.61277
C	1.013198	-3.60137	-0.8934
C	1.854439	-0.57003	-3.0969
H	2.83814	-0.2161	-2.75507
H	1.120256	0.167337	-2.74115
H	1.851998	-0.54183	-4.19634
C	0.981754	-5.34784	-4.31285
H	1.978747	-5.65892	-4.67055
H	0.427631	-4.98123	-5.19178
H	0.471308	-6.24657	-3.9356
C	0.760383	-4.07754	0.523625
H	0.169126	-5.00537	0.513624
H	0.230533	-3.34438	1.144111
H	1.702795	-4.28703	1.053122
C	2.174914	-1.61011	1.364959
C	3.447885	-2.24067	1.273588
C	4.133648	-2.63807	2.429076

H	5.108077	-3.12552	2.32076
C	3.616565	-2.43127	3.711318
C	2.388158	-1.775	3.800502
H	1.968316	-1.57017	4.790196
C	1.673094	-1.35353	2.666553
C	0.374153	-0.62372	2.932585
H	-0.503091	-1.17632	2.563089
H	0.351235	0.371162	2.464473
H	0.232327	-0.47931	4.013022
C	4.122755	-2.55079	-0.04708
H	5.207112	-2.67107	0.096094
H	3.961944	-1.77838	-0.80793
H	3.737702	-3.48657	-0.4823
C	4.355988	-2.9012	4.93869
H	3.977764	-2.4134	5.849637
H	5.435857	-2.69642	4.862467
H	4.246166	-3.99096	5.078135
N	4.396914	4.471875	-0.5498
N	5.392152	5.319343	-0.63377
C	6.771958	4.892447	-0.74696
H	7.418574	5.776863	-0.78282
H	6.93296	4.295759	-1.66328
H	7.064579	4.274558	0.119447
C	5.058782	6.726807	-0.74563
H	5.300786	7.119253	-1.74934
H	5.611891	7.319106	0.00124
H	3.982378	6.834831	-0.56928

Cartesian coordinates of 3 (B3LYP-D3(BJ)/def2-SVP, CH_2Cl_2, PCM model) in S_1 state

E = -2603.049545 Hartrees

Symbol	X	Y	Z
C	2.179776	0.328674	-0.28358
N	-0.04544	-0.56808	0.049848
S	-1.69167	1.427145	-0.00969
O	-4.2867	2.390484	0.073068
B	1.488159	-1.13399	-0.04894
C	-5.10941	1.487694	0.13474
S	1.823302	2.960972	-0.27889
O	-6.36078	-1.87047	0.3053
C	-6.59388	1.660471	0.181143
C	-7.34284	2.834222	0.164098
H	-6.85039	3.807131	0.110173
C	-8.73792	2.721687	0.218027
H	-9.35485	3.623002	0.206309
C	-9.35945	1.463473	0.287274
H	-10.4495	1.407035	0.328265

C	-8.60161	0.285566	0.304322
H	-9.07472	-0.69688	0.358057
C	-7.21538	0.402312	0.250372
C	-6.1706	-0.66726	0.252801
C	-4.86087	0.033589	0.17855
C	-1.26341	-1.13039	0.093231
H	-1.36904	-2.21316	0.131157
C	-2.32839	-0.23624	0.091153
C	-3.68177	-0.6573	0.159046
H	-3.79859	-1.7476	0.203579
C	-0.08745	0.789256	-0.04927
C	1.198194	1.33534	-0.19399
C	3.461776	0.909206	-0.38353
H	4.396404	0.351406	-0.44625
C	3.450565	2.30534	-0.40997
C	4.583048	3.179436	-0.52894
H	5.568877	2.703375	-0.59353
C	1.381059	-2.27006	-1.22153
C	1.546313	-1.96583	-2.60006
C	1.414465	-2.96569	-3.57376
H	1.555463	-2.69862	-4.6259
C	1.092838	-4.28696	-3.24727
C	0.88001	-4.57469	-1.89874
H	0.597412	-5.59247	-1.61193
C	1.013088	-3.60191	-0.89287
C	1.853633	-0.57072	-3.09693
H	2.837517	-0.21678	-2.75567
H	1.11959	0.1666	-2.74081
H	1.850595	-0.54267	-4.19637
C	0.981871	-5.34899	-4.31208
H	1.979017	-5.6615	-4.6681
H	0.429693	-4.98194	-5.19202
H	0.469724	-6.24693	-3.93527
C	0.760256	-4.07789	0.524208
H	0.168642	-5.00547	0.514338
H	0.230704	-3.34448	1.144617
H	1.702748	-4.28781	1.053428
C	2.174568	-1.6099	1.365466
C	3.447744	-2.2402	1.274482
C	4.133831	-2.63655	2.430186
H	5.108431	-3.1237	2.322101
C	3.616849	-2.42897	3.712392
C	2.388105	-1.77319	3.801209
H	1.968231	-1.56793	4.790799
C	1.672679	-1.35283	2.666994
C	0.373223	-0.62382	2.932807

H	-0.50358	-1.17728	2.563587
H	0.34943	0.370823	2.464238
H	0.23138	-0.47907	4.013194
C	4.122626	-2.55106	-0.046
H	5.207049	-2.67067	0.0971
H	3.961304	-1.77937	-0.80746
H	3.737967	-3.4875	-0.48019
C	4.356731	-2.89752	4.940029
H	3.977507	-2.41018	5.850795
H	5.436298	-2.69112	4.863967
H	4.24864	-3.98743	5.079591
N	4.39741	4.470941	-0.55205
N	5.392221	5.318032	-0.63847
C	6.772303	4.891309	-0.75036
H	7.418385	5.775882	-0.79043
H	6.932647	4.291044	-1.66428
H	7.065621	4.277041	0.118438
C	5.059961	6.726661	-0.73947
H	5.311311	7.12806	-1.73713
H	5.606491	7.311923	0.017839
H	3.982068	6.833194	-0.57167

Cartesian coordinates of 3 (B3LYP-D3(BJ)/def2-SVP, acetone, PCM model) in S_1 state

E = -2603.054009 Hartrees

Symbol	X	Y	Z
C	2.214658	0.316038	-0.27255
N	-0.02398	-0.55341	0.058058
S	-1.67091	1.451245	0.009026
O	-4.27817	2.426941	0.067494
B	1.512743	-1.14201	-0.02941
C	-5.08683	1.51257	0.130584
S	1.830004	2.951554	-0.31456
O	-6.30425	-1.83378	0.311147
C	-6.57371	1.680591	0.174444
C	-7.32676	2.842772	0.152408
H	-6.84239	3.81926	0.095742
C	-8.71932	2.720061	0.204765
H	-9.34238	3.6166	0.188981
C	-9.32729	1.464209	0.277183
H	-10.4164	1.397804	0.316965
C	-8.55983	0.294713	0.299319
H	-9.02484	-0.69114	0.355913
C	-7.18216	0.424167	0.247005
C	-6.12103	-0.63051	0.254204
C	-4.83163	0.074753	0.17937

C	-1.22064	-1.1055	0.103333
H	-1.33735	-2.18702	0.142111
C	-2.30264	-0.19682	0.099756
C	-3.63717	-0.62238	0.165692
H	-3.75572	-1.71032	0.216304
C	-0.05901	0.809922	-0.03395
C	1.219221	1.33841	-0.19568
C	3.471303	0.889583	-0.38033
H	4.411189	0.339546	-0.43438
C	3.462965	2.299173	-0.43496
C	4.569752	3.159349	-0.56684
H	5.563658	2.7059	-0.63603
C	1.39465	-2.26797	-1.21086
C	1.552812	-1.95559	-2.5836
C	1.404769	-2.94174	-3.56293
H	1.53872	-2.66476	-4.6127
C	1.076973	-4.25807	-3.24917
C	0.871919	-4.55528	-1.90633
H	0.580337	-5.57204	-1.62726
C	1.019411	-3.59725	-0.89633
C	1.870942	-0.56496	-3.07891
H	2.880274	-0.24238	-2.78848
H	1.175812	0.187532	-2.68206
H	1.811179	-0.52698	-4.17523
C	0.953639	-5.31031	-4.31774
H	1.939968	-5.72958	-4.57626
H	0.526721	-4.89646	-5.243
H	0.319416	-6.14573	-3.98877
C	0.764796	-4.08751	0.512616
H	0.177841	-5.01619	0.489576
H	0.227772	-3.36421	1.13672
H	1.705198	-4.29975	1.041914
C	2.172101	-1.63255	1.389618
C	3.449249	-2.24618	1.309366
C	4.131067	-2.6351	2.463322
H	5.113431	-3.10623	2.360171
C	3.60344	-2.44119	3.738084
C	2.365946	-1.81375	3.818234
H	1.932191	-1.62566	4.804535
C	1.653916	-1.40158	2.684044
C	0.330288	-0.72144	2.952278
H	-0.52478	-1.32248	2.609815
H	0.253149	0.261606	2.469115
H	0.19817	-0.56405	4.030884
C	4.135745	-2.54796	-0.00421
H	5.218552	-2.66011	0.14685

H	3.975208	-1.7771	-0.76539
H	3.76131	-3.48619	-0.44073
C	4.345996	-2.89222	4.966902
H	3.889074	-2.4913	5.882489
H	5.398449	-2.57116	4.940998
H	4.34864	-3.99115	5.050005
N	4.364412	4.460843	-0.60115
N	5.340769	5.299201	-0.71968
C	6.725212	4.892003	-0.82306
H	7.351386	5.785315	-0.91358
H	6.880533	4.254478	-1.70901
H	7.034244	4.326616	0.071571
C	5.008565	6.708306	-0.74616
H	5.337575	7.16521	-1.69291
H	5.500771	7.236439	0.085776
H	3.922297	6.802582	-0.65038

Cartesian coordinates of 3 (B3LYP-D3(BJ)/def2-SVP, EtOH, PCM model) in S_1 state

E = -2603.053361 Hartrees

Symbol	X	Y	Z
C	2.179982	0.326253	-0.28648
N	-0.04592	-0.56817	0.050874
S	-1.69067	1.428186	-0.0107
O	-4.28787	2.393008	0.072153
B	1.48682	-1.13534	-0.04808
C	-5.11043	1.489695	0.134784
S	1.826091	2.958837	-0.28634
O	-6.36039	-1.86838	0.310622
C	-6.59479	1.662288	0.179887
C	-7.34423	2.835656	0.160094
H	-6.85286	3.809023	0.104729
C	-8.73943	2.722686	0.213161
H	-9.35664	3.62371	0.199274
C	-9.3605	1.464377	0.284257
H	-10.4505	1.40753	0.324493
C	-8.60211	0.286709	0.304111
H	-9.07571	-0.69543	0.359318
C	-7.21588	0.403895	0.251033
C	-6.17049	-0.6647	0.256144
C	-4.86124	0.036039	0.181298
C	-1.26406	-1.12953	0.0968
H	-1.37098	-2.21203	0.138051
C	-2.32859	-0.23462	0.093791
C	-3.68175	-0.65495	0.163256
H	-3.79808	-1.74513	0.210352

C	-0.08669	0.789426	-0.0504
C	1.199016	1.333951	-0.19697
C	3.462275	0.904702	-0.39042
H	4.396282	0.346139	-0.45583
C	3.453058	2.301348	-0.41938
C	4.585549	3.173741	-0.54201
H	5.570791	2.697731	-0.61254
C	1.378913	-2.27326	-1.21909
C	1.543118	-1.97079	-2.5983
C	1.410336	-2.972	-3.57077
H	1.550552	-2.7063	-4.62336
C	1.088805	-4.29299	-3.2424
C	0.876733	-4.57891	-1.89321
H	0.593812	-5.59617	-1.60488
C	1.010759	-3.60469	-0.88867
C	1.85013	-0.57644	-3.09758
H	2.835071	-0.22295	-2.75896
H	1.117289	0.16162	-2.74054
H	1.844659	-0.54983	-4.19703
C	0.977309	-5.35659	-4.30563
H	1.974519	-5.67455	-4.65663
H	0.431112	-4.98854	-5.18882
H	0.459427	-6.25138	-3.9292
C	0.758396	-4.07871	0.529095
H	0.164304	-5.00467	0.520805
H	0.231384	-3.34331	1.149212
H	1.701357	-4.29114	1.056642
C	2.173885	-1.60843	1.367342
C	3.44769	-2.23803	1.277524
C	4.135927	-2.62898	2.433949
H	5.111023	-3.11525	2.326534
C	3.620468	-2.41666	3.716186
C	2.390533	-1.76269	3.80384
H	1.971336	-1.5546	4.793126
C	1.672779	-1.34794	2.668703
C	0.371571	-0.6217	2.933862
H	-0.50376	-1.17863	2.566533
H	0.344268	0.371712	2.462895
H	0.230218	-0.47493	4.014027
C	4.121507	-2.55332	-0.04244
H	5.206205	-2.67094	0.099752
H	3.95823	-1.78494	-0.80675
H	3.737499	-3.49239	-0.47166
C	4.363358	-2.87818	4.944721
H	3.978196	-2.39477	5.855038
H	5.440982	-2.66142	4.869855

H	4.266058	-3.96914	5.083741
N	4.399848	4.466269	-0.56251
N	5.393069	5.311976	-0.6597
C	6.773966	4.885712	-0.76801
H	7.417933	5.7706	-0.82692
H	6.930294	4.27024	-1.67179
H	7.071564	4.286943	0.110351
C	5.065566	6.72457	-0.71379
H	5.356615	7.163728	-1.68356
H	5.583029	7.27811	0.087143
H	3.982181	6.826498	-0.58298

Cartesian coordinates of 3 (B3LYP-D3(BJ)/def2-SVP, MeCN, PCM model) in S_1 state

E = -2603.054059 Hartrees

Symbol	X	Y	Z
C	2.180026	0.32573	-0.28699
N	-0.04601	-0.56821	0.051136
S	-1.69046	1.42837	-0.01092
O	-4.28805	2.393504	0.071952
B	1.486556	-1.13566	-0.04789
C	-5.11059	1.490113	0.134809
S	1.826675	2.958366	-0.28769
O	-6.36034	-1.86792	0.311764
C	-6.59493	1.662694	0.179688
C	-7.34443	2.836004	0.159354
H	-6.85325	3.809441	0.10368
C	-8.73966	2.72298	0.212287
H	-9.35691	3.62396	0.197976
C	-9.36067	1.464667	0.283782
H	-10.4507	1.407766	0.323898
C	-8.6022	0.287031	0.304188
H	-9.07591	-0.69504	0.359712
C	-7.21596	0.404272	0.251246
C	-6.17049	-0.66416	0.256879
C	-4.8613	0.036536	0.181881
C	-1.2642	-1.12938	0.097559
H	-1.37138	-2.21182	0.13949
C	-2.32863	-0.23431	0.094339
C	-3.68176	-0.65449	0.164135
H	-3.79801	-1.74464	0.211774
C	-0.08653	0.789419	-0.05059
C	1.199202	1.333635	-0.1975
C	3.462398	0.903758	-0.39164
H	4.396271	0.345024	-0.45755
C	3.453575	2.300495	-0.42108
C	4.586077	3.172576	-0.54439

H	5.571212	2.696577	-0.61594
C	1.378411	-2.27389	-1.21861
C	1.542488	-1.97177	-2.59795
C	1.409388	-2.9732	-3.5702
H	1.54952	-2.70778	-4.62286
C	1.087662	-4.29409	-3.24149
C	0.875686	-4.57965	-1.89218
H	0.592556	-5.59677	-1.60356
C	1.010034	-3.60519	-0.88788
C	1.849696	-0.57761	-3.09767
H	2.834879	-0.22437	-2.7595
H	1.117185	0.160715	-2.7405
H	1.843835	-0.55127	-4.19712
C	0.975887	-5.35795	-4.30443
H	1.97304	-5.67683	-4.65477
H	0.430562	-4.98972	-5.18808
H	0.457094	-6.25221	-3.92799
C	0.757726	-4.07883	0.530015
H	0.162831	-5.00427	0.522026
H	0.231532	-3.34291	1.150187
H	1.700756	-4.29214	1.057123
C	2.173803	-1.60822	1.367688
C	3.447708	-2.23773	1.278024
C	4.136469	-2.62753	2.434555
H	5.111643	-3.11365	2.327216
C	3.621439	-2.41416	3.716827
C	2.39128	-1.76054	3.804322
H	1.972307	-1.55182	4.79357
C	1.672962	-1.34698	2.669045
C	0.371433	-0.62127	2.934135
H	-0.50363	-1.17888	2.567231
H	0.343418	0.371893	2.462686
H	0.23023	-0.47409	4.014262
C	4.121205	-2.55397	-0.04188
H	5.205946	-2.67134	0.100081
H	3.957618	-1.78619	-0.80672
H	3.737177	-3.49348	-0.47013
C	4.365038	-2.8742	4.945498
H	3.978831	-2.39147	5.855724
H	5.442266	-2.65545	4.870724
H	4.26985	-3.96533	5.084527
N	4.400348	4.465276	-0.56451
N	5.393254	5.310783	-0.66371
C	6.774299	4.884609	-0.77142
H	7.417863	5.769519	-0.83385
H	6.929863	4.266318	-1.67328

H	7.072726	4.288758	0.10869
C	5.066567	6.72391	-0.70956
H	5.365027	7.169604	-1.67393
H	5.5784	7.271571	0.099102
H	3.982308	6.825323	-0.58583

Cartesian coordinates of 2 (B3LYP/def2-SVP, volume) in S_0 state.

E = -2987.350817 Hartrees

Symbol	X	Y	Z
C	-0.739979	1.069559	-0.174509
N	1.670706	0.98563	0.11471
S	2.340121	-1.517514	0.089845
O	4.314195	-3.47916	0.146677
B	0.486381	2.15082	-0.02444
C	5.436701	-3.000308	0.164803
S	-1.486323	-1.475204	-0.033622
O	7.971994	-0.456406	0.207092
C	6.721145	-3.769748	0.187434
C	6.917938	-5.149819	0.192132
H	6.063044	-5.829647	0.178334
C	8.235119	-5.623405	0.214859
H	8.424171	-6.699948	0.219064
C	9.323524	-4.733634	0.232341
H	10.340765	-5.133194	0.249784
C	9.121238	-3.34831	0.227661
H	9.956786	-2.644859	0.241017
C	7.808612	-2.881099	0.20503
C	7.303252	-1.470954	0.195292
C	5.81393	-1.569489	0.168936
C	3.017138	0.980356	0.116488
H	3.567742	1.920007	0.110092
C	3.613366	-0.274095	0.124948
C	5.027315	-0.454662	0.152064
H	5.591576	0.487885	0.159623
C	1.14982	-0.265252	0.063091
C	-0.257114	-0.241952	-0.045866
C	-2.154666	1.051365	-0.225111
H	-2.784393	1.939391	-0.296224
C	-2.708854	-0.224989	-0.175935
C	-4.11326	-0.553246	-0.223949
H	-4.810163	0.294212	-0.288361
C	1.048635	3.064524	-1.279867
C	0.724904	2.80231	-2.643328
C	1.273605	3.580063	-3.673327
H	0.993669	3.353352	-4.707381
C	2.170537	4.62419	-3.429642

C	2.513662	4.86305	-2.099446
H	3.223659	5.664725	-1.871576
C	1.980666	4.113772	-1.034952
C	-0.21064	1.691238	-3.075044
C	2.757681	5.430944	-4.562304
C	2.44686	4.529939	0.349819
C	0.077413	2.961894	1.361032
C	-0.821803	4.062375	1.221414
C	-1.268535	4.770043	2.346232
H	-1.952782	5.612226	2.196735
C	-0.874025	4.440193	3.645971
C	-0.02543	3.342379	3.785767
H	0.28468	3.038524	4.790664
C	0.440386	2.598977	2.686855
C	1.324244	1.414284	3.023509
C	-1.328719	4.56128	-0.119558
C	-1.351445	5.237054	4.835689
N	-4.500479	-1.787295	-0.187291
N	-5.792013	-2.132738	-0.220164
C	-6.830061	-1.138506	-0.283677
C	-8.810462	0.828472	-0.405652
C	-7.3491	-0.596976	0.899211
C	-7.296161	-0.694224	-1.5275
C	-8.288141	0.2886	-1.585461
C	-8.340607	0.385761	0.835187
C	-6.105644	-3.5148	-0.184878
C	-6.739186	-6.260553	-0.115707
C	-7.44752	-3.938232	-0.202393
C	-5.082336	-4.480692	-0.13245
C	-5.405847	-5.83613	-0.098296
C	-7.752331	-5.300872	-0.167999
H	-8.255167	-3.207418	-0.24235
H	-8.800797	-5.609991	-0.182228
H	-6.983572	-7.324841	-0.08894
H	-4.598415	-6.572108	-0.05781
H	-4.044567	-4.153151	-0.118999
H	-6.974492	-0.953497	1.861254
H	-8.748251	0.805924	1.757839
H	-9.586364	1.596469	-0.453227
H	-8.654976	0.632575	-2.555595
H	-6.881025	-1.126463	-2.440646
H	-0.266669	1.64005	-4.172616
H	-1.234934	1.837608	-2.699705
H	0.114434	0.703582	-2.715717
H	-1.55793	3.755626	-0.828688
H	-0.584327	5.199256	-0.622004

H	3.380397	5.10916	0.279498
H	2.624219	3.68812	1.031773
H	3.316281	6.303455	-4.191231
H	1.973476	5.796304	-5.245759
H	3.452936	4.824926	-5.169133
H	1.702125	5.164561	0.855732
H	-2.241244	5.161973	0.015984
H	-0.913175	6.25033	4.842716
H	-2.446814	5.364064	4.824565
H	-1.077427	4.751692	5.784638
H	1.409756	1.299257	4.113845
H	2.348478	1.523441	2.633064
H	0.924671	0.467118	2.630192

Cartesian coordinates of 3 (B3LYP/def2-SVP, volume) in S_0 state.

E = -2604.140351 Hartrees

Symbol	X	Y	Z
C	2.174044	0.336514	-0.309766
N	-0.049179	-0.567209	0.071852
S	-1.698472	1.42887	-0.050464
O	-4.311775	2.398637	0.017052
B	1.509645	-1.142198	-0.045014
C	-5.130252	1.498674	0.117813
S	1.79501	2.965803	-0.349774
O	-6.373332	-1.858992	0.397937
C	-6.61709	1.66739	0.184193
C	-7.369561	2.840238	0.150025
H	-6.875561	3.810523	0.062115
C	-8.762571	2.727367	0.232274
H	-9.382012	3.627655	0.208702
C	-9.380584	1.469738	0.3453
H	-10.470399	1.413063	0.407693
C	-8.620684	0.294222	0.37882
H	-9.087001	-0.689678	0.466706
C	-7.234566	0.411197	0.296815
C	-6.18789	-0.660944	0.310885
C	-4.877384	0.04371	0.199411
C	-1.275018	-1.11874	0.130259
H	-1.385648	-2.200282	0.195426
C	-2.339411	-0.225972	0.102323
C	-3.698178	-0.644626	0.189307
H	-3.819328	-1.733836	0.265505
C	-0.095307	0.782413	-0.066976
C	1.189696	1.335623	-0.234651
C	3.450602	0.935508	-0.427048

H	4.391481	0.385729	-0.478905
C	3.424617	2.327249	-0.474831
C	4.559054	3.209698	-0.602483
H	5.542737	2.725043	-0.669887
C	1.372409	-2.295886	-1.21861
C	1.543092	-2.020871	-2.607199
C	1.370071	-3.030285	-3.565531
H	1.515244	-2.780605	-4.621709
C	1.003772	-4.335185	-3.223953
C	0.796	-4.597395	-1.870227
H	0.485477	-5.602811	-1.567576
C	0.970072	-3.61874	-0.875104
C	1.904769	-0.650622	-3.143584
H	2.896886	-0.315882	-2.804828
H	1.191165	0.125694	-2.829839
H	1.918446	-0.659643	-4.243701
C	0.844245	-5.405132	-4.276577
H	1.824869	-5.744945	-4.653889
H	0.27799	-5.035369	-5.146985
H	0.320137	-6.288272	-3.880719
C	0.722616	-4.090067	0.547925
H	0.104873	-5.001382	0.545102
H	0.222509	-3.347503	1.18298
H	1.663934	-4.33256	1.066566
C	2.230124	-1.612127	1.371413
C	3.505689	-2.242678	1.266892
C	4.215064	-2.623669	2.415757
H	5.188818	-3.109526	2.2921
C	3.726963	-2.403649	3.705819
C	2.498044	-1.750684	3.809644
H	2.096975	-1.534496	4.805156
C	1.75693	-1.345265	2.686635
C	0.461419	-0.616436	2.985099
H	-0.427269	-1.17216	2.645991
H	0.421464	0.379242	2.518114
H	0.348867	-0.46803	4.068955
C	4.170919	-2.568714	-0.058113
H	5.256338	-2.692311	0.079375
H	4.013047	-1.802324	-0.826965
H	3.784275	-3.507564	-0.48649
C	4.489423	-2.858858	4.926362
H	4.212009	-2.276103	5.81841
H	5.577752	-2.765225	4.7829
H	4.285289	-3.920394	5.154227
N	4.386176	4.496527	-0.633536
N	5.388781	5.34483	-0.778149

C	6.770896	4.913749	-0.789075
H	7.418365	5.785782	-0.946627
H	6.95355	4.195503	-1.607007
H	7.060569	4.425506	0.162486
C	5.076055	6.745147	-0.565109
H	5.622348	7.376725	-1.28405
H	5.333195	7.080539	0.458637
H	3.997645	6.878573	-0.714995

5. References

1. Lakowicz, J.R. *Principles of Fluorescence Spectroscopy*; Kluwer Academic/Plenum Publishers: New York, 1999
2. a) N. G. Connelly, W. E. Geiger *Chem. Rev.*, **1996**, *96*, 877-910; b) W. N. Hansen, G. J. Hansen *Phys. Rev. A* **1987**, *36*, 1396-1402; c) C. M. Cardona, W. Li, A. E. Kaifer, D. Stockdale, G. C. Bazan, *Adv. Mater.* **2011**, *23*, 2367-2371.
3. M. W. Wong, K. B. Wiberg, M. J. Frisch, *J. Comp. Chem.* **1995**, *16*, 385-394.
4. Becke, A. D. Density-Functional Exchange-Energy Approximation with Correct Asymptotic Behaviour. *Phys. Rev. A* **1988**, *38*, 3098–3100
5. Lee, C.; Yang, W.; Parr, R.G. Development of the Colle-Salvetti Correlatin-Energy Formula into a Functional of the Electron Density. *Phys. Rev. B* **1988**, *37*, 785–789.
6. Becke, A. D. Density-Functional Thermochemistry. III. The Role of Exact Exchange. *J. Chem. Phys.* **1993**, *98*, 5648–5652.
7. Weigend, F.; Ahlrichs, R. Balanced Basis Sets of Split Valence, Triple Zeta Valence and Quadruple Zeta Valence Quality for H to Rn: Design and Assessment of Accuracy. *Phys. Chem. Chem. Phys.* **2005**, *7*, 3297–3305.
